# Ice2 promotes ER membrane biogenesis in yeast by inhibiting the conserved lipin phosphatase complex

**DOI:** 10.15252/embj.2021107958

**Published:** 2021-10-06

**Authors:** Dimitrios Papagiannidis, Peter W Bircham, Christian Lüchtenborg, Oliver Pajonk, Giulia Ruffini, Britta Brügger, Sebastian Schuck

**Affiliations:** ^1^ Center for Molecular Biology of Heidelberg University (ZMBH) DKFZ‐ZMBH Alliance and Cell Networks Cluster of Excellence Heidelberg Germany; ^2^ Heidelberg University Biochemistry Center (BZH) Heidelberg Germany; ^3^ Present address: Laboratory of Systems Biology VIB Center for Microbiology / Laboratory of Genetics and Genomics CMPG KU Leuven Leuven Belgium

**Keywords:** endoplasmic reticulum, lipid droplets, lipin, Opi1, organelle biogenesis, Membranes & Trafficking

## Abstract

Cells dynamically adapt organelle size to current physiological demand. Organelle growth requires membrane biogenesis and therefore needs to be coordinated with lipid metabolism. The endoplasmic reticulum (ER) can undergo massive expansion, but the underlying regulatory mechanisms are largely unclear. Here, we describe a genetic screen for factors involved in ER membrane expansion in budding yeast and identify the ER transmembrane protein Ice2 as a strong hit. We show that Ice2 promotes ER membrane biogenesis by opposing the phosphatidic acid phosphatase Pah1, called lipin in metazoa. Specifically, Ice2 inhibits the conserved Nem1‐Spo7 complex and thus suppresses the dephosphorylation and activation of Pah1. Furthermore, Ice2 cooperates with the transcriptional regulation of lipid synthesis genes and helps to maintain cell homeostasis during ER stress. These findings establish the control of the lipin phosphatase complex as an important mechanism for regulating ER membrane biogenesis.

## Graphical abstract

A screen for factors involved in ER membrane expansion identifies a role for ER transmembrane protein Ice2 via inhibition of the Nem1‐Spo7/Pah1‐lipin phosphatase cascade.

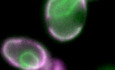

## Introduction

Cells reshape and resize their organelles when they undergo differentiation or adapt to changing conditions. An increase in organelle size typically involves enhanced membrane biogenesis, which in turn requires an adequate supply of lipids. Thus, organelle biogenesis depends on lipid synthesis and on the cellular decision whether to consume available lipids for energy production, employ them as building blocks for new membranes, or store them for future use. Accordingly, the regulatory mechanisms that control lipid synthesis and utilization are fundamental for organelle biogenesis.

The ER is a morphologically complex organelle with essential functions in protein folding and lipid synthesis (Westrate *et al*, 2015). It forms the nuclear envelope and extends into the cytoplasm as an intricate network. The principal structural elements of the ER are tubules and sheets (Shibata *et al*, 2010). In addition, intermediate structures exist, including tubular matrices and fenestrated sheets (Puhka *et al*, 2012; Nixon‐Abell *et al*, 2016; Schroeder *et al*, 2019). A variety of ER morphologies can arise according to physiological demand, ranging from mainly tubular ER, for example, in lipid hormone‐producing cells of the testes, to mainly sheet‐like ER, for example, in secretory cells of the pancreas (Fawcett, 1981). Adaptation to changing cellular need can also profoundly impact ER size. For instance, the ER expands several‐fold when B lymphocytes differentiate into antibody‐secreting plasma cells or when cells face protein folding stress in the ER (Wiest *et al*, 1990; Bernales *et al*, 2006). Such stress‐induced ER expansion is mediated by the unfolded protein response (UPR), which induces genes encoding ER‐resident protein folding enzymes to restore homeostasis (Walter & Ron, 2011). Besides raising the abundance of protein folding enzymes, the UPR also drives the biogenesis of the ER membrane. It does so, at least in part, by inducing genes that encode lipid synthesis enzymes (Sriburi *et al*, 2007; Bommiasamy *et al*, 2009; Schuck *et al*, 2009).

Yeast synthesize membrane phospholipids primarily from phosphatidic acid (PA) through the CDP‐DAG pathway (Henry *et al*, 2012). Many enzymes of this pathway are controlled transcriptionally by the activators Ino2/4 and the repressor Opi1. Ino2 and Ino4 form a heterodimer that binds to promoter elements of lipid synthesis genes. Opi1 inhibits Ino2/4 by binding to Ino2 (Heyken *et al*, 2005). Repression of Ino2/4 by Opi1 is relieved when accumulating PA tethers Opi1 to the ER membrane, sequestering it away from the nucleoplasm (Loewen *et al*, 2004). Thus, the PA‐Opi1‐Ino2/4 system forms a feedback loop that matches PA availability to the cellular capacity for converting PA into other phospholipids. Removal of Opi1 results in activation of lipid synthesis and ER membrane expansion, even in cells lacking the UPR. This membrane expansion without a corresponding upregulation of the protein folding machinery increases cellular resistance to ER stress, highlighting the physiological importance of ER membrane biogenesis (Schuck *et al*, 2009). However, it is unknown whether activation of Ino2/4 is the only mechanism regulating the production of ER membrane. Furthermore, neither Ino2/4 nor Opi1 is conserved in metazoa. Therefore, yeast could regulate ER membrane biogenesis in unique ways. Alternatively, conserved regulators of lipid metabolism distinct from Ino2/4 and Opi1 could determine ER size in both yeast and higher eukaryotes.

Here, we systematically search for genes involved in ER membrane biogenesis in budding yeast, *Saccharomyces cerevisiae* and define Ice2 as an important element in the regulatory circuitry that connects lipid metabolism and organelle biogenesis.

## Results

### An inducible system for ER membrane biogenesis

**Figure 1:**
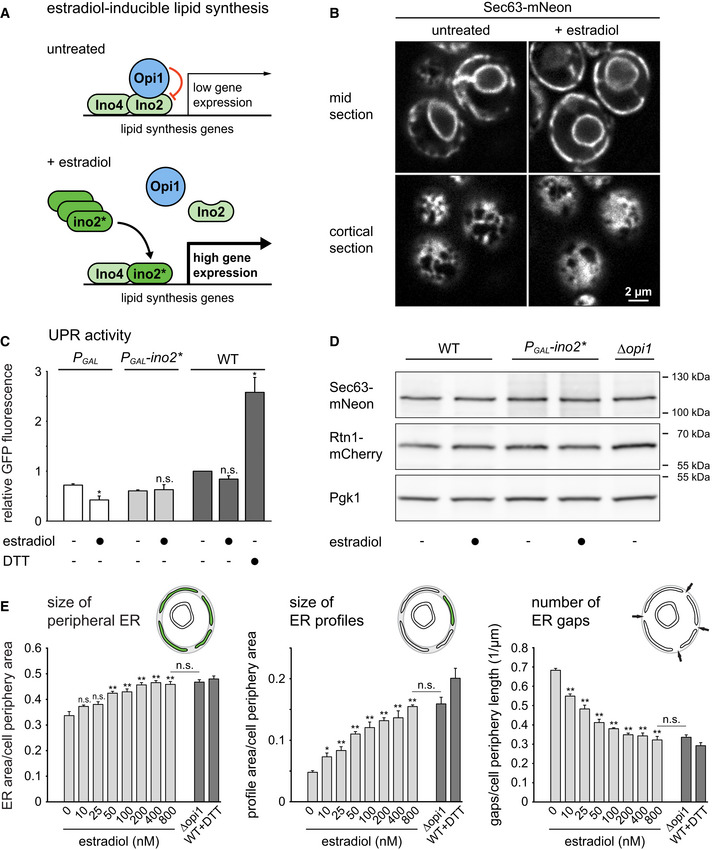
An inducible system for ER membrane biogenesis Schematic of the control of lipid synthesis by estradiol‐inducible expression of ino2*.Sec63‐mNeon images of mid and cortical sections of cells harboring the estradiol‐inducible system (SSY1405). Cells were untreated or treated with 800 nM estradiol for 6 h.Flow cytometric measurements of GFP levels in cells containing the transcriptional UPR reporter. WT cells containing the UPR reporter (SSY2306), cells additionally harboring an estradiol‐inducible *GAL* promoter (SSY2307), and cells additionally harboring the system for estradiol‐inducible expression of ino2* under the *GAL* promoter (SSY2308) were untreated, treated with 800 nM estradiol for 6 h, or treated with 8 mM DTT for 1 h. Data were normalized to untreated WT cells. Bars represent the mean of three biological replicates (*n* = 3), and error bars are the standard error of the mean (s.e.m.). Asterisks indicate statistical significance compared with the corresponding untreated sample, as judged by a two‐tailed Student’s *t*‐test assuming equal variance. Exceptions were the tests against the normalized value for wild‐type cells, for which a two‐tailed Student's *t*‐test with unequal variance was applied. *P < 0.05; n.s., not significant. WT, wild‐type.Western blot of Sec63, mCherry, and Pgk1 from WT cells (SSY1404), cells harboring the estradiol‐inducible system (SSY1405) or *∆opi1* cells (SSY1607), all of which expressed Sec63‐mNeon and Rtn1‐mCherry. Cells were untreated or treated with 800 nM estradiol for 6 h. Pgk1 served as a loading control.Quantification of ER size in estradiol‐treated cells harboring the inducible system (SSY1405), untreated *∆opi1* cells (SSY1607) and WT cells (SSY1404) treated with 8 mM DTT for 1 h. Bars represent mean + s.e.m., *n* = 3 biological replicates. Asterisks indicate statistical significance compared with 0 nM estradiol or *∆opi1* cells, as judged by a two‐tailed Student’s *t*‐test assuming equal variance. *P < 0.05; **P < 0.01; n.s., not significant. Source data are available online for this figure.

**Figure EV1:**
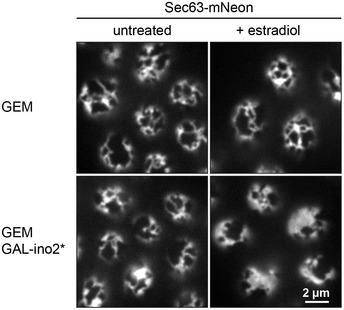
An inducible system for ER membrane biogenesis Sec63‐mNeon images of cortical sections of cells containing the estradiol‐inducible artificial transcription factor GEM (SSY2328) and cells additionally containing ino2* under the control of the *GAL* promoter (SSY1405). Cells were untreated or treated with 800 nM estradiol for 6 h. Source data are available online for this figure.

Removal of Opi1 induces Ino2/4‐controlled lipid synthesis genes and thereby leads to expansion of the ER (Schuck *et al*, 2009). To improve experimental control over ER membrane biogenesis, we developed an inducible system using ino2(L119A), an Ino2 variant that cannot be inhibited by Opi1 (Heyken *et al*, 2005). We placed ino2(L119A), here termed ino2*, under the control of the *GAL* promoter and employed an expression system that activates this promoter upon addition of the metabolically inert sterol ß‐estradiol (Pincus *et al*, 2014). High‐level expression of ino2* is expected to displace endogenous Ino2 from the promoters of its target genes, stimulate lipid synthesis, and drive ER membrane biogenesis (Fig 1A; Schuck *et al*, 2009). Fluorescence microscopy confirmed that the expression of ino2* triggered pronounced ER expansion. In untreated cells, the peripheral ER at the cell cortex mostly consisted of tubules, which appeared as short membrane profiles in optical mid sections and as a network in cortical sections. In contrast, estradiol‐treated cells had a peripheral ER that predominantly consisted of ER sheets, as evident from long membrane profiles in mid sections and solid membrane areas in cortical sections (Fig 1B). Cells not expressing ino2* showed no change in ER morphology upon estradiol treatment (Fig EV1).

To test whether ino2* expression causes ER stress and may in this way indirectly cause ER expansion, we measured UPR activity by means of a transcriptional reporter. This reporter is based on UPR response elements controlling expression of GFP (Jonikas *et al*, 2009). Cell treatment with the ER stressor DTT activated the UPR reporter, as expected, whereas expression of ino2* did not (Fig 1C). Furthermore, neither expression of ino2* nor removal of Opi1 altered the abundance of the chromosomally tagged ER proteins Sec63‐mNeon or Rtn1‐mCherry, even though the *SEC63* gene is regulated by the UPR (Fig 1D; Pincus *et al*, 2014). These observations indicate that ino2* expression does not cause ER stress but induces ER membrane expansion as a direct result of enhanced lipid synthesis.

To assess ER membrane biogenesis quantitatively, we developed three metrics for the size of the peripheral ER at the cell cortex as visualized in mid sections: (i) total size of the peripheral ER, (ii) size of individual ER profiles, and (iii) number of gaps between ER profiles (Fig 1E). These metrics are less sensitive to uneven image quality than the index of expansion we had used previously (Schuck *et al*, 2009). The expression of ino2* with different concentrations of estradiol resulted in a dose‐dependent increase in peripheral ER size and ER profile size and a decrease in the number of ER gaps (Fig 1E). The ER of cells treated with 800 nM estradiol was indistinguishable from that in *∆opi1* cells, and we used this concentration in subsequent experiments.

These results show that the inducible system allows titratable control of ER membrane biogenesis without causing ER stress.

### A genetic screen for regulators of ER membrane biogenesis

**Figure 2:**
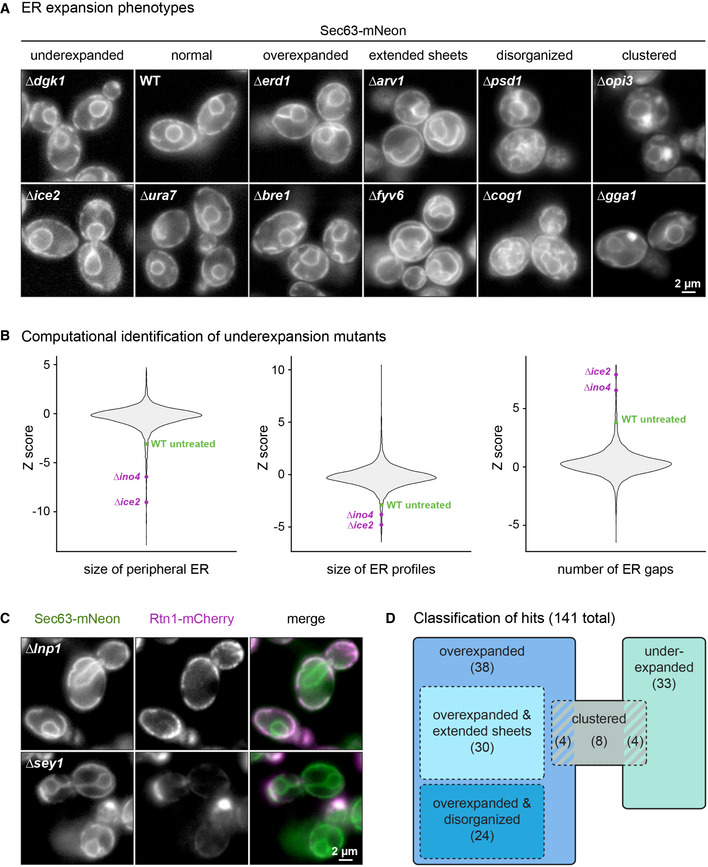
A genetic screen for factors involved in ER membrane biogenesis Sec63‐mNeon images of cells of the indicated genotypes harboring the inducible system. Cells were treated with 800 nM estradiol for 6 h. Two examples of each phenotypic class are shown.Violin plots of Z scores from the three metrics for ER size determined for each of the 4,800 mutant strains. The untreated WT is shown for reference.Sec63‐mNeon and Rtn1‐mCherry images of *∆lnp1* and *∆sey1* cells harboring the inducible system and treated with 800 nM estradiol for 6 h.Classification of hits. Numbers in brackets indicate the number of mutants in each class. Striped areas indicate mutants belonging to two classes. Source data are available online for this figure.

To identify genes involved in ER expansion, we introduced the inducible ER biogenesis system and the ER marker proteins Sec63‐mNeon and Rtn1‐mCherry into a knockout strain collection. This collection consisted of single gene deletion mutants for most of the approximately 4800 non‐essential genes in yeast (Giaever *et al*, 2002). We induced ER expansion by ino2* expression and acquired images by automated microscopy. Based on inspection of Sec63‐mNeon in mid sections, we defined six phenotypic classes. Mutants were grouped according to whether their ER was (i) underexpanded, (ii) properly expanded and hence morphologically normal, (iii) overexpanded, (iv) overexpanded with extended cytosolic sheets, (v) overexpanded with disorganized cytosolic structures, or (vi) clustered. Fig 2A shows two examples of each class. To refine the search for mutants with an underexpanded ER, we applied the three ER size metrics described above in an automated fashion (Fig 2B and Dataset EV1A). This analysis confirmed the underexpansion mutants identified visually and retrieved a number of additional, weaker hits.

In total, we found 141 mutants that fell into at least one phenotypic class other than morphologically normal (Dataset EV1B). Hits included mutants lacking the ER‐shaping gene *LNP1*, which had an overexpanded peripheral ER with large gaps, and mutants lacking the homotypic ER fusion gene *SEY1*, which displayed ER clusters (Fig 2C; Hu *et al*, 2009; Chen *et al*, 2012). The identification of these known ER morphogenesis genes validated our approach. About two‐thirds of the identified mutants had an overexpanded ER, one‐third had an underexpanded ER, and a small number of mutants showed ER clusters (Fig 2D). Overexpansion mutants were enriched in gene deletions that activate the UPR (Dataset EV1C; Jonikas *et al*, 2009). This enrichment suggested that ER expansion in these mutants resulted from ER stress rather than enforced lipid synthesis. Indeed, re‐imaging of the overexpansion mutants revealed that their ER was expanded already without ino2* expression. Underexpansion mutants included those lacking *INO4* or the lipid synthesis genes *OPI3*, *CHO2,* and *DGK1*. In addition, mutants lacking *ICE2* showed a particularly strong underexpansion phenotype (Fig 2A and B).

Overall, our screen indicated that a large number of genes impinge on ER membrane biogenesis, as might be expected for a complex biological process. The functions of many of these genes in ER biogenesis remain to be uncovered. Here, we follow up on *ICE2* because of its critical role in building an expanded ER. Ice2 is a polytopic ER membrane protein (Estrada de Martin *et al*, 2005) but does not possess obvious domains or sequence motifs that provide clues to its molecular function.

### Ice2 promotes ER membrane biogenesis

**Figure 3:**
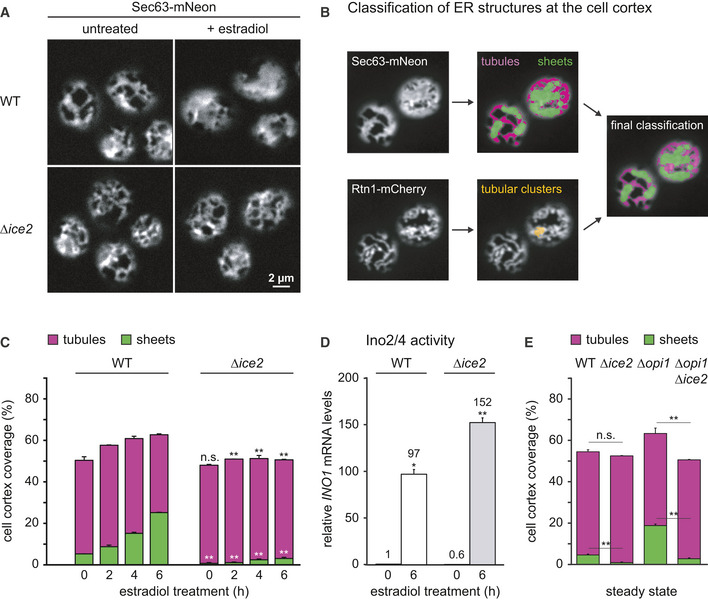
Ice2 is required for ER membrane biogenesis upon activation of Ino2/4 Sec63‐mNeon images of the cortical ER of WT and *Δice2* cells harboring the inducible system (SSY1405, 1603). Cells were untreated or treated with 800 nM estradiol for 6 h.Classification of peripheral ER structures from cortical sections of cells expressing Sec63‐mNeon and Rtn1‐mCherry as tubules (purple), sheets (green), or tubular clusters (yellow). Tubular clusters are combined with tubules in the final classification, as illustrated by the overlay.Quantification of peripheral ER structures in WT and *∆ice2* cells harboring the inducible system (SSY1405, 1603) and treated with 800 nM estradiol for the times indicated. Bars are the mean percentage of cell cortex covered by tubules (purple) or sheets (green), *n* = 3 biological replicates. Upper error bars are s.e.m. for the sum of tubules and sheets, and lower error bars are s.e.m. for sheets. Asterisks indicate statistical significance compared with the corresponding value in WT cells, as judged by a two‐tailed Student’s *t*‐test assuming equal variance. **P < 0.01; n.s., not significant.mRNA levels of the Ino2/4 target gene *INO1* upon ino2* expression in WT and *Δice2* cells harboring the inducible system (SSY1405, 1603) as measured by quantitative real‐time PCR. Data were normalized to untreated WT cells. Mean + s.e.m., *n* = 3 biological replicates. Asterisks indicate statistical significance compared with the corresponding untreated cells, as judged by a two‐tailed Student’s *t*‐test assuming equal variance. An exception was the test against the normalized value for WT cells, for which a two‐tailed Student's *t*‐test with unequal variance was applied. *P < 0.05; **P < 0.01.Quantification of peripheral ER structures in untreated WT, *Δice2*, *Δopi1,* and *Δice2 Δopi1* cells (SSY1404, 2356, 2595, 2811). Bars are the mean percentage of cell cortex covered by tubules (purple) or sheets (green), *n* = 3 biological replicates. Upper error bars are s.e.m. for the sum of tubules and sheets, and lower error bars are s.e.m. for sheets. Asterisks indicate statistical significance compared with the corresponding value in WT cells, as judged by a two‐tailed Student’s *t*‐test assuming equal variance. **P < 0.01; n.s., not significant. Source data are available online for this figure.

To more precisely define the contribution of Ice2 to ER membrane biogenesis, we analyzed optical sections of the cell cortex. Well‐focused cortical sections are more difficult to acquire than mid sections but provide more morphological information. Qualitatively, deletion of *ICE2* had little effect on ER structure at steady state but severely impaired ER expansion upon ino2* expression (Fig 3A).

To describe ER morphology quantitatively, we developed a semi‐automated algorithm that classifies ER structures as tubules or sheets based on images of Sec63‐mNeon and Rtn1‐mCherry in cortical sections (Fig 3B). First, the image of the general ER marker Sec63‐mNeon is used to segment the entire ER. Second, morphological opening, that is the operation of erosion followed by dilation, is applied to the segmented image to remove narrow structures. The structures removed by this step are defined as tubules, and the remaining structures are provisionally classified as sheets. Third, the same procedure is applied to the image of Rtn1‐mCherry, which marks high‐curvature ER (Westrate *et al*, 2015). Rtn1 structures that remain after morphological opening and overlap with persistent Sec63 structures are termed tubular clusters. These structures appear as sheets in the Sec63 image but the overlap with Rtn1 identifies them as tubules. Tubular clusters may correspond to so‐called tubular matrices observed in mammalian cells (Nixon‐Abell *et al*, 2016) and made up only a minor fraction of the total ER. Last, for a simple two‐way classification, tubular clusters are added to the tubules and any remaining Sec63 structures are defined as sheets. This analysis using a general and a high‐curvature ER marker allows to distinguish densely packed tubules from sheets.

The algorithm described above showed that the ER covered approximately 50% of the cell cortex in untreated wild‐type cells and consisted mostly of tubules, as reported (Fig 3C; Hu *et al*, 2008; Schuck *et al*, 2009; West *et al*, 2011). The expression of ino2* triggered ER expansion by stimulating the formation of sheets. *∆ice2* cells had a defect in sheet formation already at steady state, and membrane expansion upon ino2* expression failed almost completely. Importantly, ino2* still activated the prototypic Ino2/4 target gene *INO1* in *∆ice2* cells, ruling out that *ICE2* deletion disrupted the inducible ER biogenesis system (Fig 3D). In addition, *ICE2* deletion abolished the constitutive ER expansion in *∆opi1* cells, excluding that the expansion defect in *∆ice2* cells merely reflected a delay (Fig 3E).

**Figure 4:**
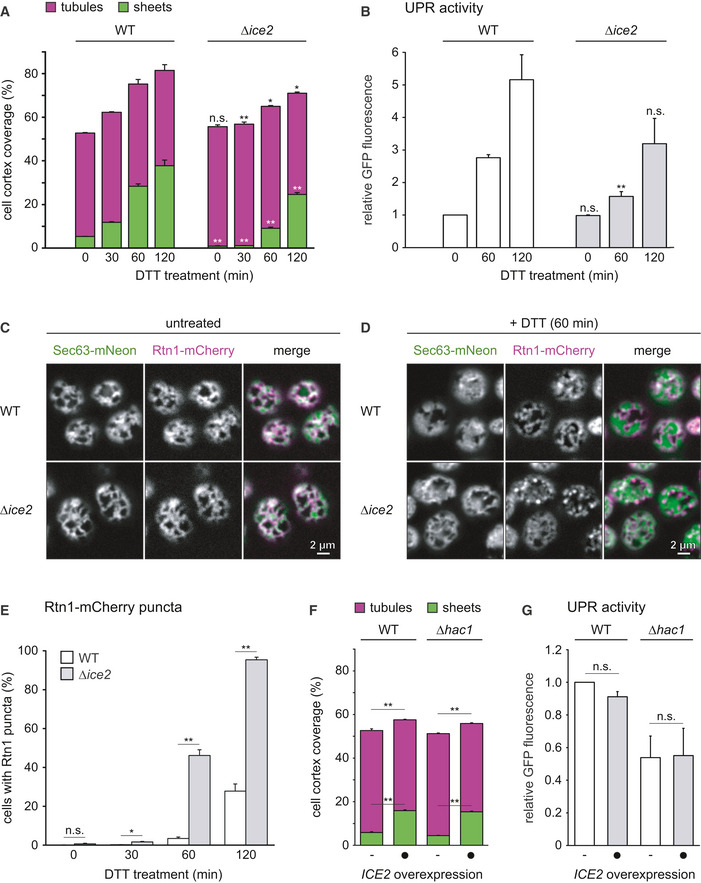
Ice2 is required for ER membrane biogenesis upon ER stress, and *ICE2* overexpression is sufficient to induce ER expansion AQuantification of peripheral ER structures in WT and *∆ice2* cells (SSY1405, 1603) treated with 8 mM DTT for the times indicated. Bars are the mean percentage of cell cortex covered by tubules (purple) or sheets (green), *n* = 3 biological replicates. Upper error bars are s.e.m. for the sum of tubules and sheets, and lower error bars are s.e.m. for sheets. Asterisks indicate statistical significance compared with the corresponding value in WT cells, as judged by a two‐tailed Student’s *t*‐test assuming equal variance. *P < 0.05; **P < 0.01; n.s., not significant.BFlow cytometric measurements of GFP levels of WT and *Δice2* cells containing the transcriptional UPR reporter (SSY2306, 2312). Cells were treated with 8 mM DTT for the times indicated. Data were normalized to untreated WT cells. Mean + s.e.m., *n* = 3 biological replicates. Asterisks indicate statistical significance compared with the corresponding value in WT cells, as judged by a two‐tailed Student’s *t*‐test assuming equal variance. **P < 0.01; n.s., not significant.C, DFluorescence images of cortical sections of WT and *Δice2* cells expressing Sec63‐mNeon and Rtn1‐mCherry (SSY1405, 1603) that were untreated (C) or treated with 8 mM DTT for 1 h (D).EQuantification of WT and *∆ice2* cells with Rtn1‐mCherry puncta after treatment with 8 mM DTT for the times indicated. Mean + s.e.m., *n* = 3 biological replicates. Asterisks indicate statistical significance compared with the corresponding value in WT cells, as judged by a two‐tailed Student’s *t*‐test assuming equal variance. *P < 0.05; **P < 0.01; n.s., not significant.FQuantification of peripheral ER structures in untreated WT and UPR‐deficient *∆hac1* cells (SSY2228, 2331), overexpressing *ICE2* from plasmid pSS761 where indicated. Bars are the mean percentage of cell cortex covered by tubules (purple) or sheets (green), *n* = 3 biological replicates. Upper error bars are s.e.m. for the sum of tubules and sheets, and lower error bars are s.e.m. for sheets. Asterisks indicate statistical significance compared with the corresponding value in WT cells, as judged by a two‐tailed Student’s *t*‐test assuming equal variance. **P < 0.01.GFlow cytometric measurements of GFP levels of WT and *Δhac1* cells containing the UPR reporter (SSY2306, 2314) and overexpressing *ICE2* from plasmid pSS761 where indicated. Data were normalized to untreated WT cells. Mean + s.e.m., *n* = 3 biological replicates. Asterisks indicate statistical significance compared with the corresponding untreated sample, as judged by a two‐tailed Student’s *t*‐test assuming equal variance. An exception was the test against the normalized value for WT cells, for which a two‐tailed Student's *t*‐test with unequal variance was applied. n.s., not significant. Source data are available online for this figure.

**Figure EV2:**
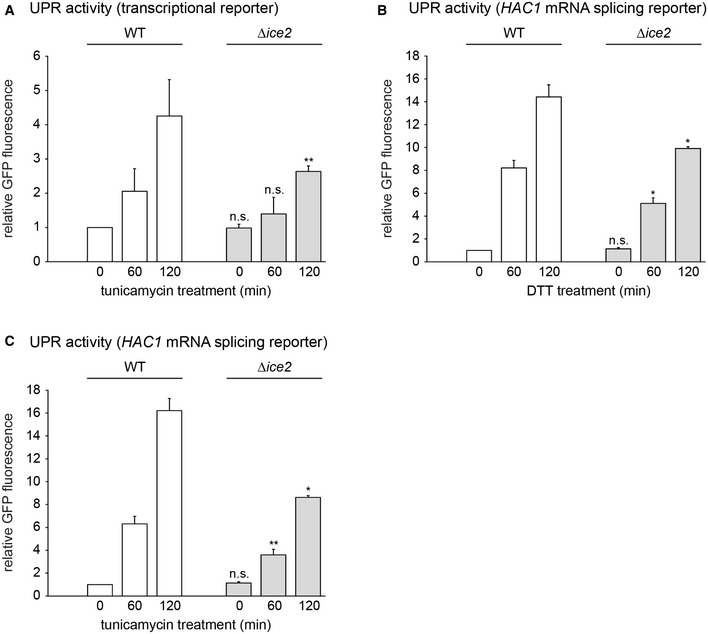
Deletion of *ICE2* impairs UPR signaling during ER stress AFlow cytometric measurements of GFP levels of WT and *Δice2* cells containing the transcriptional UPR reporter (SSY2306, 2312). Cells were treated with 1 μg/ml tunicamycin for the times indicated. Data were normalized to untreated WT cells. Mean + s.e.m., *n* = 3 biological replicates. Asterisks indicate statistical significance compared with the corresponding value in WT cells, as judged by a two‐tailed Student’s *t*‐test assuming equal variance. An exception was the test against the normalized value for WT cells, for which a two‐tailed Student's *t*‐test with unequal variance was applied. **P < 0.01; n.s., not significant.B, CFlow cytometric measurements of GFP levels of WT and *Δice2* cells containing the *HAC1* mRNA splicing reporter (SSY2309, 2313). Cells were treated with 8 mM DTT (B) or 1 μg/ml tunicamycin (C) for the times indicated. Data were normalized to untreated WT cells. Mean + s.e.m., *n* = 3 biological replicates. Asterisks indicate statistical significance compared with the corresponding value in WT cells, as judged by a two‐tailed Student’s *t*‐test assuming equal variance. Exceptions were the tests against the normalized values for WT cells, for which a two‐tailed Student's *t*‐test with unequal variance was applied. *P < 0.05; **P < 0.01; n.s., not significant. Source data are available online for this figure.

Next, we tested whether Ice2 was required for ER expansion induced by ER stress. DTT treatment of wild‐type cells triggered rapid ER expansion, which was again driven by the formation of sheets (Fig 4A). Image quantification suggested that ER expansion was retarded in *∆ice2* cells. Furthermore, loss of Ice2 diminished UPR induction by the ER stressors DTT and tunicamycin, as judged by the transcriptional reporter as well as an alternative UPR reporter based on *HAC1* mRNA splicing (Figs 4B and EV2A–C; Pincus *et al*, 2010). This reduced activation of the UPR may be explained by defective clustering of the UPR signal transducer Ire1 in the absence of Ice2 (Cohen *et al*, 2017). However, closer inspection of images of wild‐type and *∆ice2* cells revealed that ER expansion in *∆ice2* mutants was not simply retarded but aberrant. Specifically, DTT induced striking puncta positive for Rtn1‐mCherry but not Sec63‐mNeon, and these puncta were much more abundant in *∆ice2* than in wild‐type cells (Fig 4C–E). It remains to be determined whether these puncta are aberrant membrane structures or Rtn1‐mCherry molecules not associated with the ER membrane. In any case, these data show that removal of Ice2 impairs ER expansion also during ER stress.

Finally, we asked whether raising Ice2 levels leads to ER expansion. Indeed, overexpression of *ICE2* caused ER expansion, and this still occurred in cells that cannot activate the UPR due to deletion of *HAC1* (Fig 4F; Emmerstorfer *et al*, 2015). In addition, *ICE2* overexpression did not activate the UPR (Fig 4G). Hence, Ice2 can drive ER membrane biogenesis independently of the UPR.

Collectively, these data show that Ice2 is required for and promotes ER membrane biogenesis. This impact of Ice2 is neither the result of disrupted Ino2/4 target gene induction in the absence of Ice2 nor of UPR activation upon *ICE2* overexpression.

### Ice2 is functionally linked to Nem1, Spo7, and Pah1

**Figure EV3:**
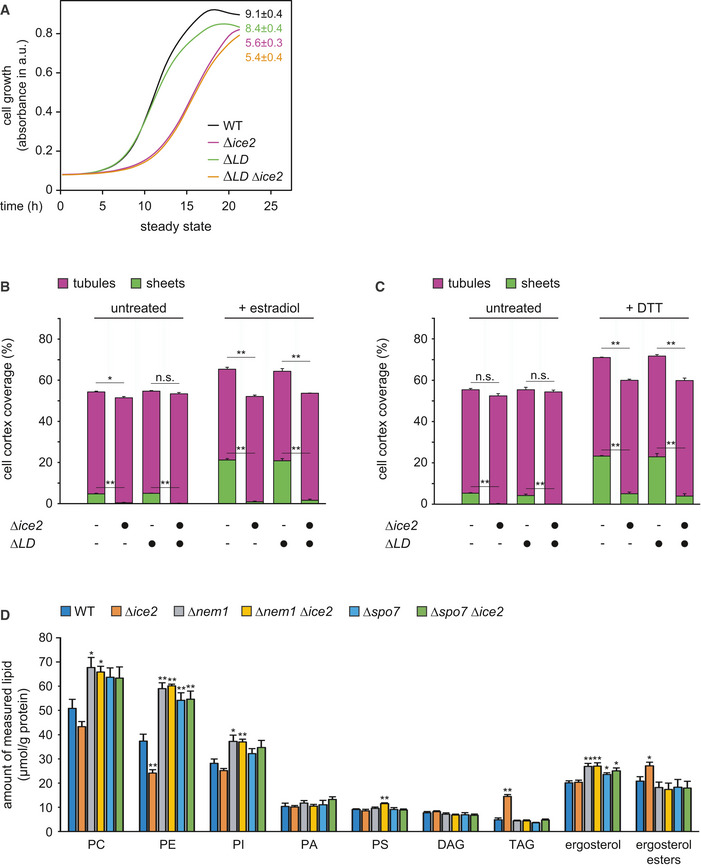
Absence of lipid droplets has no effect on ER expansion in WT or *∆ice2* cells AGrowth assays of untreated WT, *Δice2*, *ΔLD,* and *ΔLD Δice2* cells (SSY2228, 2229, 2230, 2256). Numbers represent areas under the curves and serve as growth indices. Mean + s.e.m., *n* = 3 biological replicates. *∆LD*, *∆lipid droplet*.B, CQuantification of peripheral ER structures in WT, *Δice2*, *ΔLD,* and *ΔLD Δice2* cells harboring the inducible system (SSY2598, 2599, 2600, 2601), which were untreated or treated with either 800 nM estradiol for 6 h (B) or 8 mM DTT for 1 h (C). Bars are the mean percentage of cell cortex covered by tubules (purple) or sheets (green), *n* = 3 biological replicates. Upper error bars are s.e.m. for the sum of tubules and sheets, and lower error bars are s.e.m. for sheets. Asterisks indicate statistical significance, as judged by a two‐tailed Student’s *t*‐test assuming equal variance. *P < 0.05; **P < 0.01; n.s., not significant.DLipidomic analysis of WT, *Δice2*, *Δnem1*, *Δice2 Δnem1*, *Δspo7,* and *Δice2 Δspo7* cells (SSY1404, 2356, 2482, 2484, 2481, 2483). Mean + s.e.m., *n* = 4 biological replicates. Asterisks indicate statistical significance compared with WT cells, as judged by a two‐tailed Student’s *t*‐test assuming equal variance. *P < 0.05; **P < 0.01. The data are the same as in Fig 5C and D but are shown as lipid‐to‐protein ratios in µg measured lipid per g total protein. Source data are available online for this figure.

Ice2 has been implicated in ER morphogenesis and lipid metabolism, yet its function has not been defined in molecular terms (Estrada de Martin *et al*, 2005; Loewen *et al*, 2007; Tavassoli *et al*, 2013; Markgraf *et al*, 2014; Quon *et al*, 2018). One proposal is that Ice2 channels diacylglycerol (DAG) from lipid droplets (LDs) to the ER for phospholipid synthesis (Markgraf *et al*, 2014). We therefore first asked whether defective ER membrane biogenesis in *∆ice2* cells resulted from an insufficient supply of lipids from LDs. Deletion of *ICE2* impairs cell growth (Markgraf *et al*, 2014). Abolishing LD formation by combined deletion of *ARE1*, *ARE2*, *LRO1,* and *DGA1* (Sandager *et al*, 2002) did not affect growth, and deletion of *ICE2* still impaired growth in the absence of LDs (Fig EV3A). Therefore, Ice2 must have functions independent of LDs. Moreover, lack of LDs had no effect on ER expansion after ino2* expression or DTT treatment, and deletion of *ICE2* still impaired ER expansion in the absence of LDs (Fig EV3B and C). Hence, the role of Ice2 in ER membrane biogenesis cannot be explained by LD‐dependent functions. These results additionally show that ER expansion can occur without lipid mobilization from LDs.

**Figure 5:**
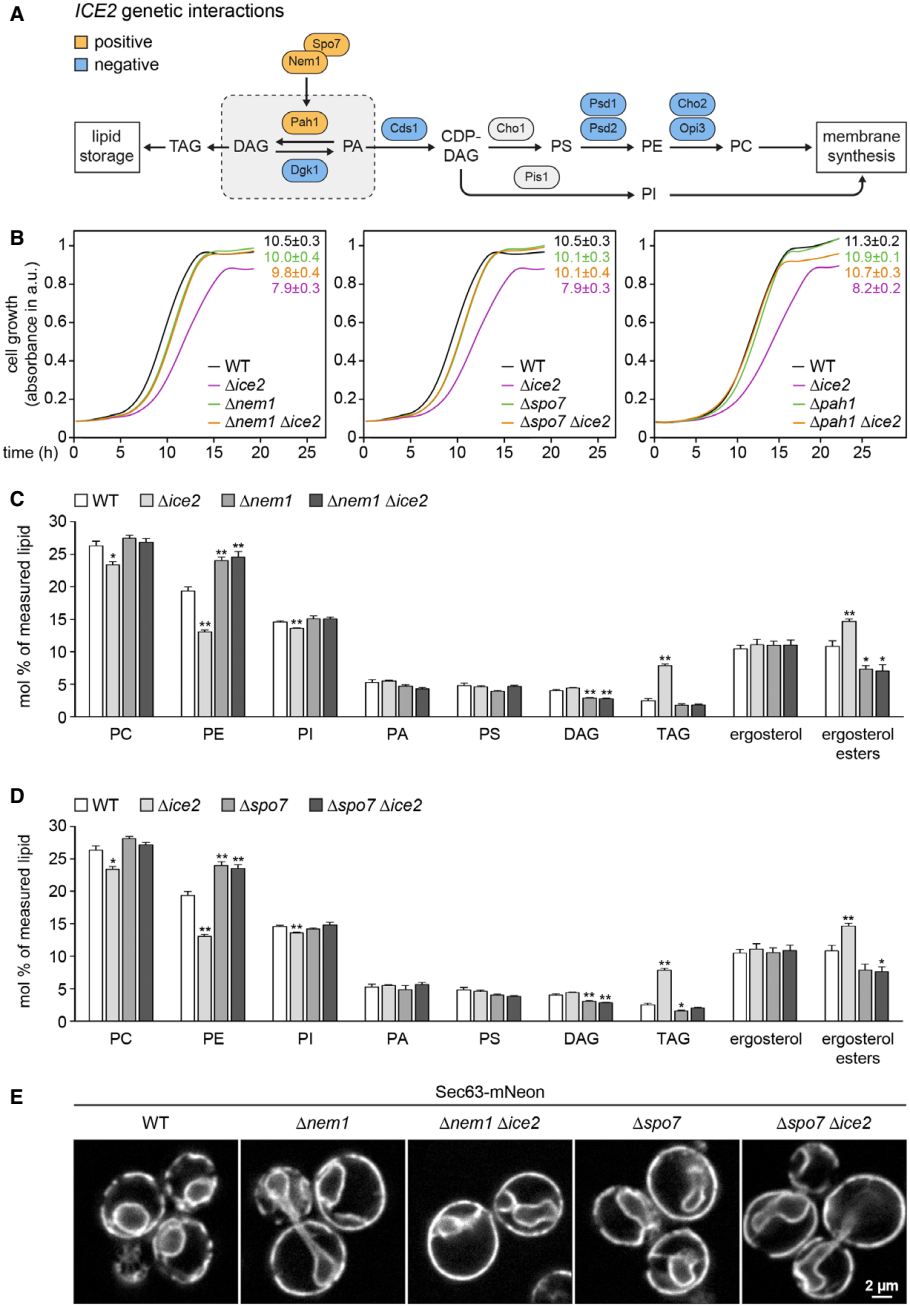
Ice2 is functionally linked to Nem1, Spo7, and Pah1 AGenetic interactions of *ICE2* with selected lipid synthesis genes. CDP, cytidine diphosphate; DAG, diacylglycerol; PA, phosphatidic acid; PI/PS/PE/PC, phosphatidylinositol/serine/ethanolamine/choline; TAG, triacylglycerol.BGrowth assays of untreated WT, *Δice2*, *Δnem1*, *Δnem1 Δice2*, *Δspo7*, *Δspo7 Δice2*, *Δpah1,* and *Δpah1 Δice2* cells (SSY1404, 2356, 2482, 2484, 2481, 2483, 2807, 2808). Numbers represent areas under the curves and serve as growth indices. Mean + s.e.m., *n* = 3 biological replicates. Data for WT and *∆ice2* cells are the same in the left and middle panels.C, DLipidomic analysis of WT, *Δice2*, *Δnem1*, *Δice2 Δnem1*, *Δspo7,* and *Δice2 Δspo7* cells (SSY1404, 2356, 2482, 2484, 2481, 2483). Mean + s.e.m., *n* = 4 biological replicates. Asterisks indicate statistical significance compared with WT cells, as judged by a two‐tailed Student’s *t*‐test assuming equal variance. *P < 0.05; **P < 0.01. Data for WT and *Δice2* cells are the same as in both panels.ESec63‐mNeon images of untreated WT, *Δnem1*, *Δnem1Δice2*, *Δspo7,* and *Δspo7 Δice2* cells (SSY1404, 2482, 2484, 2481, 2483). Source data are available online for this figure.

Genome‐scale studies have identified many genetic interactions of *ICE2* with lipid synthesis genes (Schuldiner *et al*, 2005; Costanzo *et al*, 2010; Surma *et al*, 2013). An interesting pattern emerged from mapping these data onto the biochemical pathways for membrane biogenesis and lipid storage (Fig 5A and Table EV1). *ICE2* displays negative interactions with genes for membrane lipid synthesis but positive interactions with *NEM1*, *SPO7,* and *PAH1*, which promote lipid storage. Positive genetic interactions, which cause the phenotype of a double mutant to be less severe than expected from the phenotypes of the respective single mutants, frequently reflect involvement of interacting genes in the same pathway (Costanzo *et al*, 2019). We therefore focused on the relationship of *ICE2* with *NEM1*, *SPO7,* and *PAH1*.

**Figure 6:**
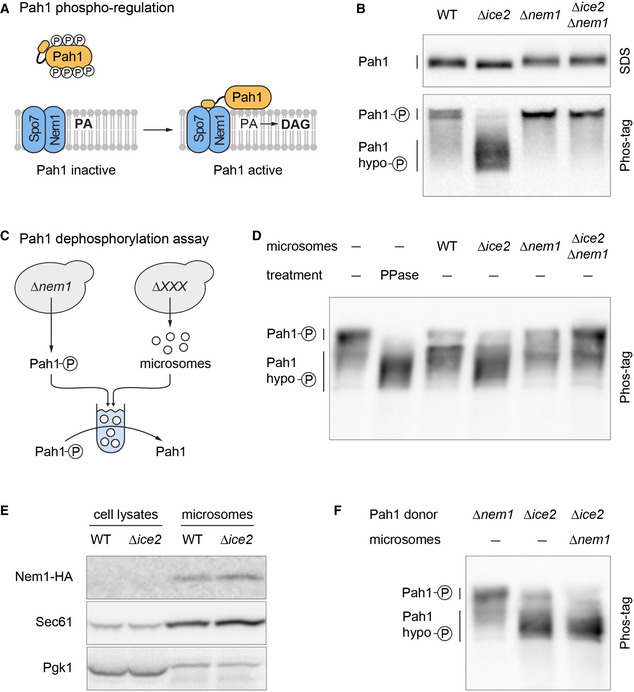
Ice2 opposes Pah1 by inhibiting the Nem1‐Spo7 complex Schematic of Pah1 phospho‐regulation. Phosphorylated Pah1 is cytosolic and inactive. Interaction of Pah1 and the ER‐localized Nem1‐Spo7 complex results in Pah1 dephosphorylation and activation, promoting conversion of PA into DAG.Western blot of HA from WT, *Δice2*, *Δnem1,* and *Δnem1 Δice2* cells expressing endogenously tagged Pah1‐HA (SSY2592, 2593, 2594, 2718). Blots of SDS‐PAGE and Phos‐tag PAGE gels are shown.Schematic of Pah1 dephosphorylation assay with phosphorylated Pah1 from *∆nem1* mutants and microsomes from different strains.Western blot of HA from Pah1 dephosphorylation reactions that contained phosphorylated Pah1‐HA from *∆nem1* mutants (SSY3065) and microsomes from cells of the indicated genotypes (SSY3053, 3074, 3075, 3095). Phosphorylated Pah1 and dephosphorylated Pah1 resulting from treatment with recombinant alkaline phosphatase (PPase) are shown for reference.Western blot of HA, Sec61, and Pgk1 from cell lysates and microsomes prepared from WT and *∆ice2* cells expressing Nem1‐HA (SSY3140, 3141). Nem1 is undetectable in cell lysates due to its low abundance.Western blot of HA from Pah1 phosphorylation reaction that contained hypophosphorylated Pah1‐HA from *∆ice2* cells (SSY3096) and microsomes from *∆nem1* cells (SSY3075). Phosphorylated Pah1 from *∆nem1* cells (SSY3065) and hypophosphorylated Pah1 from *∆ice2* cells are shown for reference. Source data are available online for this figure.

Pah1 is a phosphatidic acid phosphatase that converts PA into DAG (Fig 5A; Han *et al,* 2006). In yeast, PA is a precursor of all phospholipids, and DAG is a precursor of the storage lipid triacylglycerol (Henry *et al*, 2012). Pah1 thus promotes LD biogenesis (Adeyo *et al*, 2011). Pah1 is regulated by phosphorylation (Fig 6A). Phosphorylated Pah1 is cytosolic and inactive. Activation of Pah1 requires that it binds to and is dephosphorylated by the ER‐localized Nem1‐Spo7 complex, which consists of the phosphatase Nem1 and its binding partner Spo7 (Siniossoglou *et al*, 1998; Santos‐Rosa *et al*, 2005; Karanasios *et al*, 2013). Dephosphorylated Pah1 associates with the ER and thus gains access to its substrate, PA (O'Hara *et al*, 2006; Karanasios *et al*, 2010). Thus, the Nem1‐Spo7 complex is an immediate upstream activator of Pah1.

Growth assays confirmed the positive genetic interaction of *ICE2* with *NEM1*, *SPO7,* and *PAH1*. Remarkably, the growth defect caused by deletion of *ICE2* was completely prevented by deletion of any of the other genes (Fig 5B). This type of strong positive genetic interaction is called suppression (Costanzo *et al*, 2019). An explanation for this relationship could be that Ice2 inhibits the Nem1‐Spo7 complex and hence the activation of Pah1. In this scenario, removal of Ice2 would result in Pah1 overactivity, which is known to impair cell growth (Santos‐Rosa *et al*, 2005). Additional removal of Nem1, Spo7, or Pah1 would suppress this phenotype. Removal of Ice2 would also result in an accumulation of triacylglycerol at the expense of phospholipids, and this effect would require the Nem1‐Spo7 complex. We therefore analyzed the lipidomes of wild‐type, *∆ice2*, *∆nem1,* and *∆spo7* cells. Compared with wild‐type cells, *∆ice2* mutants had decreased levels of phosphatidylethanolamine and phosphatidylcholine and increased levels of triacylglycerol and ergosterol esters, the two lipid classes that make up LDs (Fig 5C and D; see Fig EV3D for an alternative plot showing lipid‐to‐protein ratios instead of mol % lipid). These changes agree with earlier data and confirm that *ICE2* deletion enhances lipid storage (Markgraf *et al*, 2014). The lipidomes of *∆nem1* and *∆spo7* cells showed changes in the opposite direction, confirming that *NEM1* or *SPO7* deletion enhances membrane biogenesis (Siniossoglou *et al*, 1998). Furthermore, the lipidomes of *∆nem1 ∆ice2* and *∆spo7 ∆ice2* double mutants were indistinguishable from those of *∆nem1* and *∆spo7* single mutants, confirming that loss of *ICE2* is irrelevant in the absence of *NEM1* or *SPO7*. In agreement with the lipidomic data, deletion of *NEM1* or *SPO7* drastically expanded the ER and caused nuclear morphology defects, as reported (Siniossoglou *et al*, 1998; Campbell *et al*, 2006), and additional deletion of *ICE2* had no morphological effect (Fig 5E).

Overall, these results show that Ice2, Nem1, Spo7, and Pah1 are functionally linked. Furthermore, they support the idea that Ice2 acts as an inhibitor of Nem1, Spo7, and Pah1 in a pathway that controls lipid utilization and ER membrane biogenesis.

### Ice2 opposes Pah1 by inhibiting the Nem1‐Spo7 complex

To test whether Ice2 indeed inhibits the Nem1‐Spo7/Pah1 pathway, we made use of the fact that the phosphorylation status of Pah1 is a well‐established indicator of its activity (O'Hara *et al*, 2006). When we separated different phosphoforms of Pah1 on Phos‐tag gels (Dubots *et al*, 2014), we found that removal of Ice2 caused dephosphorylation of Pah1, and this effect was dependent on Nem1 (Fig 6B). These results show that Ice2 opposes Pah1 dephosphorylation, which it could achieve by inhibiting the Nem1‐Spo7 complex. Alternatively, Ice2 could activate a Pah1 kinase. To distinguish between these possibilities, we reconstituted Pah1 dephosphorylation *in vitro* (Fig 6C). As substrate, we used phosphorylated Pah1 immunoisolated from *∆nem1* cells. As source for the transmembrane proteins Nem1, Spo7, and Ice2, we used microsomes prepared from wild‐type, *∆ice2*, *∆nem1,* or *∆ice2 ∆nem1* cells. Phosphorylated Pah1 ran as a low‐mobility band on Phos‐tag gels and shifted to higher mobility when dephosphorylated with alkaline phosphatase (Fig 6D). Incubation of phosphorylated Pah1 with microsomes from wild‐type cells caused partial Pah1 dephosphorylation. In comparison, microsomes from *∆ice2* mutants had more Pah1 phosphatase activity. In contrast, microsomes from *∆nem1* cells showed reduced phosphatase activity, and, importantly, this residual activity was not enhanced by deletion of *ICE2*. Hence, removal of Ice2 stimulated Pah1 dephosphorylation by Nem1. The levels of Nem1 in microsomes prepared from wild‐type and *∆ice2* cells were similar, ruling out that the high Nem1 activity in the absence of Ice2 resulted from increased Nem1 abundance (Fig 6E). The residual Pah1 dephosphorylation by *∆nem1* microsomes is unexpected because there is no evidence for another genuine Pah1 phosphatase besides Nem1. The activity may be an artifact of the *in vitro* assay and could stem from a phosphatase that never encounters Pah1 in cells. Next, we modified the *in vitro* assay to test whether the Pah1 phosphorylation status was affected by a kinase that could be activated by Ice2. Hypophosphorylated Pah1 immunoisolated from *∆ice2* cells was incubated with microsomes from *∆nem1* cells so that any kinase activity targeting Pah1 could manifest itself without being masked by Nem1‐mediated dephosphorylation. No phosphorylation of Pah1 was apparent (Fig 6F), indicating that our assay exclusively reconstituted Pah1 dephosphorylation. Hence, Ice2 is an inhibitor of Nem1‐mediated dephosphorylation of Pah1.

**Figure 7:**
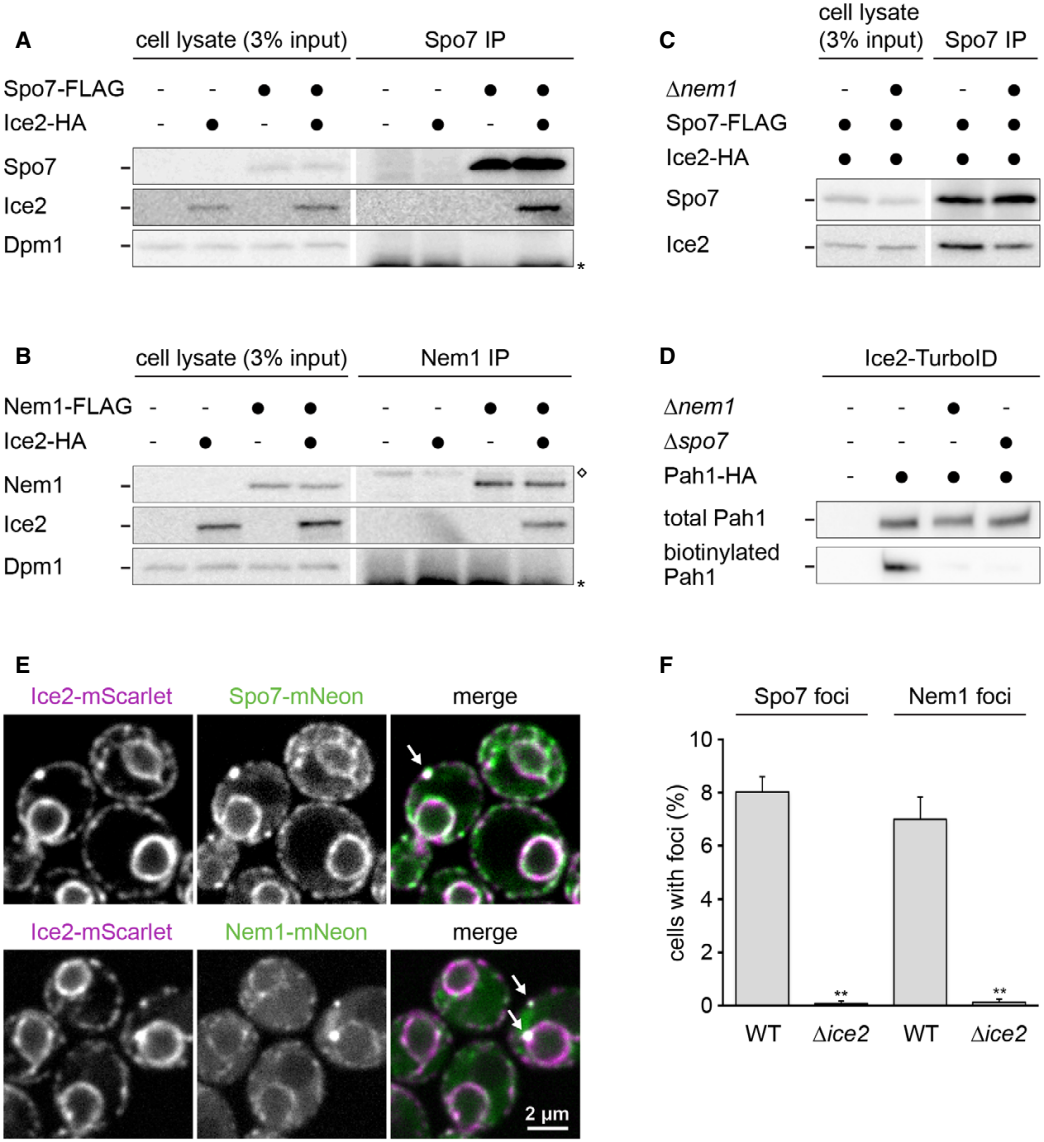
Ice2 interacts and co‐localizes with the Nem1‐Spo7 complex A–CWestern blots of FLAG, HA, and Dpm1 from cell lysates or anti‐FLAG immunoprecipitates of WT or *∆nem1* cells containing Spo7‐FLAG, Nem1‐FLAG, or Ice2‐HA as indicated (SSY122, 2421, 3183, 3184, 3195, 3196, 3197). The asterisk marks the position of the light chain from the antibody used for immunoprecipitation. The diamond marks a protein that is non‐specifically precipitated by the anti‐FLAG antibody. IP, immunoprecipitate.DWestern blot of HA from total cell lysates or biotin pulldowns of cells containing Ice2‐TurboID, Pah1‐HA, and deletions of *NEM1* or *SPO7* as indicated (SSY2978, 2979, 3117, 3118).EImages of cells with endogenously tagged Ice2‐mScarlet and Spo7‐mNeon or Nem1‐mNeon (SSY3244, 3245). Arrows indicate foci containing both Ice2 and either Spo7 or Nem1.FQuantification of Spo7 and Nem1 foci in WT and *∆ice2* cells (SSY2916, 3238, 2917, 3239). Mean + s.e.m., *n* = 3 biological replicates. Asterisks indicate statistical significance compared with the respective WT cells, as judged by a two‐tailed Student’s *t*‐test assuming equal variance. **P < 0.01. Source data are available online for this figure.

**Figure EV4:**
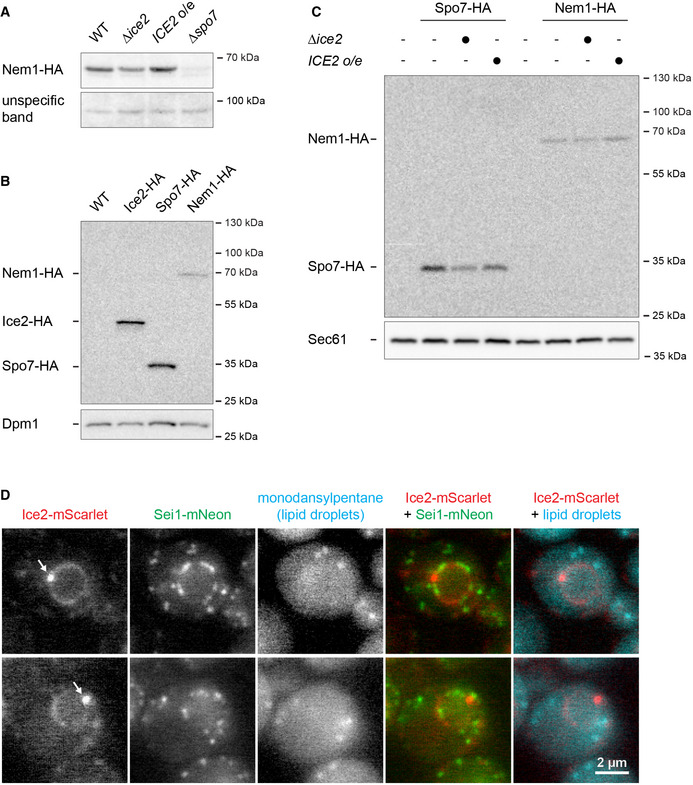
Abundance of Ice2, Spo7, and Nem1 Western blot of HA from total membranes prepared from WT, *∆ice2,* and *ICE2*‐overexpressing and *∆spo7* cells containing Nem1‐HA (SSY2913, 2914, 2915, 2945). An unspecific band served as a loading control.Western blot of HA from total cell membranes from WT cells (SSY122) and cells expressing Ice2‐HA, Spo7‐HA, or Nem1‐HA (SSY2421, 2910, 2913). Dpm1 served as a loading control.Western blot of HA from total membranes prepared from WT cells (SSY122), WT, *∆ice2,* and *ICE2*‐overexpressing cells containing Spo7‐HA (SSY2910, 2911, 2912), and WT, *∆ice2,* and *ICE2*‐overexpressing cells containing Nem1‐HA (SSY2913, 2914, 2915). Sec61 served as a loading control. o/e, overexpression.Images of cells expressing endogenously tagged Ice2‐mScarlet and Sei1‐mNeon (SSY3318) and stained with monodansylpentane to highlight lipid droplets. Arrows indicate foci containing Ice2. Source data are available online for this figure.

We next used co‐immunoprecipitation to determine whether Ice2 physically associates with the Nem1‐Spo7 complex. We chromosomally fused *SPO7* or *NEM1* with a FLAG tag and *ICE2* with an HA tag, solubilized the proteins with detergent, and retrieved Spo7‐FLAG or Nem1‐FLAG with anti‐FLAG antibodies. Ice2 co‐precipitated with both Spo7 and Nem1, but not with the abundant ER transmembrane protein Dpm1 (Fig 7A and B). We were unable to test whether the association of Ice2 and Nem1 depends on Spo7 because Nem1 is unstable in the absence of Spo7 (Fig EV4A; Mirheydari *et al*, 2020). However, Ice2 still co‐precipitated with Spo7 in the absence of Nem1, albeit less efficiently (Fig 7C). Since Nem1 and Spo7 form a stable complex (Siniossoglou *et al*, 1998), these results suggest that Ice2, Nem1, and Spo7 can form a ternary complex. This notion together with the fact that the Nem1‐Spo7 complex physically interacts with Pah1 (Karanasios *et al*, 2013) implies that Ice2 is in the vicinity of a pool of Pah1. Indeed, fusion of Ice2 to the non‐specific biotin ligase TurboID (Branon *et al*, 2018) resulted in biotinylation of Pah1. This proximity‐dependent biotinylation was strongly reduced when ER recruitment of Pah1 was blocked by *NEM1* or *SPO7* deletion (Fig 7D).

To better understand the relationship of Ice2, Spo7, and Nem1, we analyzed their relative abundance and localization. Consistent with high‐throughput studies (Ho *et al*, 2018), Ice2 and Spo7 endogenously tagged with HA were similarly abundant, whereas the abundance of Nem1‐HA was much lower (Fig EV4B). Spo7 and Nem1 levels were mildly reduced by *ICE2* deletion and essentially unchanged by *ICE2* overexpression (Fig EV4C), further supporting the notion that Ice2 controls the activity of the Nem1‐Spo7 complex rather than its abundance. Ice2, Spo7, and Nem1 have been reported to distribute over the entire ER and form ER‐associated foci in the proximity of LDs (Siniossoglou *et al*, 1998; Estrada de Martin *et al*, 2005; Adeyo *et al*, 2011; Markgraf *et al*, 2014). Ice2, Nem1, and Spo7 endogenously tagged with mScarlet or mNeon indeed showed a typical ER pattern. Furthermore, we occasionally observed Ice2 foci that also contained Spo7 and Nem1 (Fig 7E). These foci did not obviously co‐localize with the LD biogenesis factor Sei1 or LDs stained with monodansylpentane (Fig EV4D). It therefore remains unclear whether these Ice2 foci are related to Nem1‐containing sites of LD biogenesis (Adeyo *et al*, 2011; Choudhary *et al*, 2020). Strikingly, however, Spo7 and Nem1 did not form foci when Ice2 was absent (Fig 7F). Hence, Ice2 clusters Spo7 and Nem1, which may help to prevent uncontrolled Pah1 activation and PA‐to‐DAG conversion across the whole ER.

Together, these findings show that Ice2 interacts with and restrains the Nem1‐Spo7 phosphatase complex, thus opposing dephosphorylation and activation of Pah1.

### Ice2 promotes ER membrane biogenesis through inhibition of Pah1

**Figure EV5:**
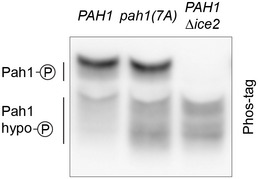
Phosphorylation status of pah1(7A) Western blot of HA from WT and *∆ice2* cells in which *PAH1* was replaced with *PAH1‐HA* or *pah1(7A)‐HA* as indicated (SSY2841, SSY2842, SSY2970). Source data are available online for this figure.

**Figure 8:**
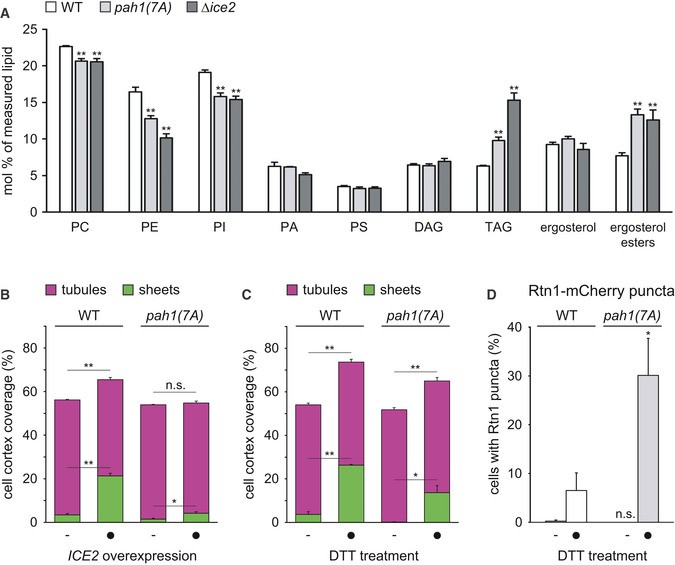
Ice2 promotes ER membrane biogenesis through inhibition of Pah1 Lipidomic analysis of WT and *∆ice2* cells in which *PAH1* was replaced with *PAH1‐HA* or *pah1(7A)‐HA* as indicated (SSY2841, SSY2842, SSY2970). Mean + s.e.m., *n* = 4 biological replicates. Asterisks indicate statistical significance compared with WT cells, as judged by a two‐tailed Student’s *t*‐test assuming equal variance. **P < 0.01.Quantification of peripheral ER structures in WT cells in which *PAH1* was replaced by *PAH1‐HA* or *pah1(7A)‐HA*, without and with overexpression of *ICE2* (SSY2841, 2842, 2843, 2844). Bars are the mean percentage of cell cortex covered by tubules (purple) or sheets (green), *n* = 3 biological replicates. Upper error bars are s.e.m. for the sum of tubules and sheets, and lower error bars are s.e.m. for sheets. Asterisks indicate statistical significance compared with the respective control cells not overexpressing *ICE2*, as judged by a two‐tailed Student’s *t*‐test assuming equal variance. *P < 0.05; **P < 0.01; n.s., not significant.Quantification of peripheral ER structures in WT cells in which *PAH1* was replaced by *PAH1‐HA* or *pah1(7A)‐HA* (SSY2841, 2842), treated with 8 mM DTT for 1 h. Mean + s.e.m., *n* = 4 biological replicates. Asterisks indicate statistical significance compared with WT cells, as judged by a two‐tailed Student’s *t*‐test assuming equal variance. *P < 0.05; **P < 0.01.Quantification of WT and *∆ice2* cells with Rtn1‐mCherry puncta after treatment with 8 mM DTT for 1 h. Mean + s.e.m., *n* = 3 biological replicates. Asterisks indicate statistical significance compared with WT cells, as judged by a two‐tailed Student’s *t*‐test assuming equal variance. *P < 0.05; n.s., not significant. Source data are available online for this figure.

We next tested whether Ice2‐mediated ER membrane biogenesis requires inhibition of Pah1. We employed pah1(7A), which carries mutations in seven of the residues that are dephosphorylated by Nem1 (O'Hara *et al*, 2006; Carman & Han, 2019). As a result, pah1(7A) is constitutively active, although some regulation by Nem1 through additional phosphorylation sites remains (Su *et al*, 2014). Accordingly, pah1(7A) was hypophosphorylated compared with wild‐type Pah1, but the activation of Nem1 by deletion of *ICE2* yielded Pah1 that carried even fewer phosphate residues (Fig EV5). In addition, replacing Pah1 with pah1(7A) shifted the levels of phospholipids, triacylglycerol, and ergosterol esters into the same direction as deletion of *ICE2*, but the shifts were less pronounced (Fig 8A). Hence, pah1(7A) is constitutively but not maximally active. If Ice2 needs to inhibit Pah1 to promote ER membrane biogenesis, then the non‐inhibitable pah1(7A) should interfere with ER expansion upon *ICE2* overexpression. Overexpression of *ICE2* expanded the ER in wild‐type cells, as before (Fig 8B, also see Fig 4F). Replacing Pah1 with pah1(7A) caused a slight shrinkage of the ER at steady state, consistent with reduced membrane biogenesis. Moreover, pah1(7A) almost completely blocked ER expansion after *ICE2* overexpression. Similarly, pah1(7A) impaired ER expansion upon DTT treatment, thus phenocopying the effects of *ICE2* deletion (Fig 8C and D, also see Fig 4A and E). These data support the notion that Ice2 promotes ER membrane biogenesis by inhibiting Pah1, although we cannot formally exclude that Ice2 acts through additional mechanisms.

### Ice2 cooperates with the PA‐Opi1‐Ino2/4 system and promotes cell homeostasis

**Figure 9:**
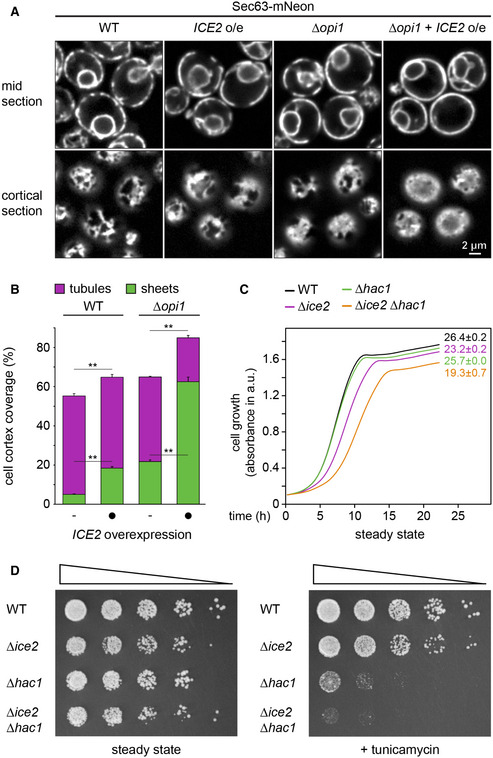
Ice2 cooperates with the PA‐Opi1‐Ino2/4 system and promotes ER homeostasis Sec63‐mNeon images of mid and cortical sections of untreated WT and *∆opi1* cells, overexpressing *ICE2* where indicated (SSY1404, 2588, 2595, 2596).Quantification of peripheral ER structures in the strains shown in panel (A). Bars are the mean percentage of cell cortex covered by tubules (purple) or sheets (green), *n* = 3 biological replicates. Upper error bars are s.e.m. for the sum of tubules and sheets, and lower error bars are s.e.m. for sheets. Asterisks indicate statistical significance compared with control cells not overexpressing *ICE2,* as judged by a two‐tailed Student’s *t*‐test assuming equal variance. **P < 0.01.Growth assays of untreated WT, *∆hac1*, *Δice2*, and *Δhac1 Δice2* cells (SSY1404, 2356, 2805, 2806). Numbers represent areas under the curves and serve as growth indices. Mean + s.e.m., *n* = 3 biological replicates.Growth assays on solid media of WT, *∆hac1*, *Δice2*, and *Δhac1 Δice2* cells (SSY1404, 2356, 2805, 2806) in the absence or presence of 0.2 μg/ml tunicamycin. For each series, cells were diluted fivefold from one step to the next. Source data are available online for this figure.

Given the important role of Opi1 in ER membrane biogenesis (Schuck *et al*, 2009), we asked how Ice2 is related to the PA‐Opi1‐Ino2/4 system. *OPI1* deletion and *ICE2* overexpression both cause ER expansion. These effects could be independent of each other or they could be linked. Combined *OPI1* deletion and *ICE2* overexpression produced an extreme ER expansion, which exceeded that in *∆opi1* mutants or *ICE2*‐overexpressing cells (Fig 9A and B). This hyperexpanded ER covered most of the cell cortex and contained an even greater proportion of sheets than the ER in DTT‐treated wild‐type cells (Fig 9B, also see Fig 4A). Therefore, Ice2 and the PA‐Opi1‐Ino2/4 system make independent contributions to ER membrane biogenesis.

Last, to gain insight into the physiological significance of Ice2, we analyzed the interplay of Ice2 and the UPR. Under standard culture conditions, *∆ice2* mutants show a modest growth defect (Fig 5B; Markgraf *et al*, 2014), and UPR‐deficient *∆hac1* mutants grow like wild‐type cells (Sidrauski *et al*, 1996). Nevertheless, *∆ice2 ∆hac1* double mutants grew slower than *∆ice2* mutants (Fig 9C). This synthetic phenotype was even more pronounced under ER stress. In the presence of the ER stressor tunicamycin, *∆ice2* mutants showed a slight growth defect, *∆hac1* mutants showed a strong growth defect, and *∆ice2 ∆hac1* double mutants showed barely any growth at all (Fig 9D). Hence, Ice2 is particularly important for cell growth when ER stress is not buffered by the UPR. These results emphasize that Ice2 promotes ER homeostasis.

## Discussion

In this study, we have systematically identified factors involved in ER membrane expansion upon enforced lipid synthesis in yeast. We show that Ice2 is critical for proper ER expansion, both upon enforced lipid synthesis and during ER stress. We find that Ice2 inhibits the Nem1‐Spo7 complex, thus opposing activation of the phosphatidic acid phosphatase Pah1 and promoting membrane biogenesis. These results uncover an additional layer of regulation of the Nem1‐Spo7/Pah1 phosphatase cascade. Finally, we provide evidence that Ice2 cooperates with the PA‐Opi1‐Ino2/4 system to regulate ER membrane biogenesis and helps to maintain ER homeostasis.

**Figure 10:**
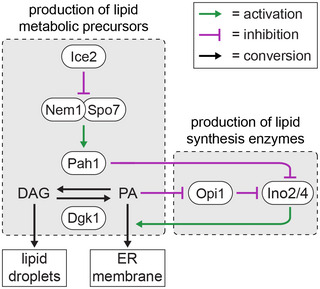
Model for the regulation of ER membrane biogenesis Pah1 converts phosphatidic acid (PA) into diacylglycerol (DAG) for lipid droplet biogenesis. Ice2 inhibits the Nem1‐Spo7 complex and hence Pah1. Ice2 thereby increases PA availability and relieves repression of Ino2/4‐driven lipid synthesis genes, thus promoting ER membrane biogenesis. These mechanisms coordinate the production of lipid metabolic precursors and lipid synthesis enzymes.

Our findings can be integrated into a model of the regulatory network that controls ER membrane biogenesis (Fig 10). At the core of this network is the interconversion of DAG and PA by Dgk1 and Pah1. Ice2 inhibits Pah1 dephosphorylation by the Nem1‐Spo7 complex and thus suppresses conversion of PA into DAG. The resulting increased availability of PA is coordinated with the production of lipid synthesis enzymes that turn PA into other phospholipids. Specifically, inhibition of Pah1 prevents it from repressing Ino2/4‐controlled lipid synthesis genes (Santos‐Rosa *et al*, 2005). In addition, PA sequesters Opi1 and thereby derepresses Ino2/4 target genes (Loewen *et al*, 2004). Hence, inhibition of Pah1 by Ice2 increases the availability of PA and, concomitantly, induces phospholipid synthesis genes.

This model readily explains the effects of *ICE2* deletion and overexpression. First, the increase in LD abundance in *∆ice2* mutants (Markgraf *et al*, 2014) may simply result from high constitutive Pah1 activity. The disruption of ino2*‐driven ER expansion by *ICE2* deletion may reflect the need to coordinate the production of lipid metabolic precursors with the expression of lipid synthesis genes. As we show, ino2* still induces genes encoding lipid synthesis enzymes in *∆ice2* mutants. Nonetheless, ER expansion fails, likely because the supply of substrates for these enzymes is limiting. The same reasoning may explain the additive effects of *OPI1* deletion and *ICE2* overexpression. *ICE2* overexpression increases PA availability and presumably releases some repression of Ino2/4 target genes. However, *OPI1* deletion likely further boosts the levels of lipid synthesis enzymes and hence ER membrane biogenesis.

Our study opens up new avenues for investigation. For instance, the structure of the proposed Ice2‐Spo7‐Nem1 complex needs to be determined and it could contain several molecules of Ice2 and Spo7 per molecule of Nem1. The structure of the complex will be essential for understanding whether Ice2 restrains the Nem1‐Spo7 complex by sequestering it into clusters poorly accessible for Pah1, by directly inhibiting the enzymatic activity of Nem1, or both. Furthermore, it needs to be elucidated whether and how Ice2 itself is regulated. The *ICE2* gene is not induced by ER stress (Pincus *et al*, 2014). A possibility to be explored is that Ice2 activity is controlled by phosphorylation, as is the case not only for Pah1 but also for Nem1 and Dgk1 (Dubots *et al*, 2014; Qiu *et al*, 2016; Su *et al*, 2018).

How could our findings from yeast apply to higher eukaryotes? Bioinformatic analysis suggests mammalian SERINC proteins as distant Ice2 orthologs (Alli‐Balogun & Levine, 2021), but whether SERINC proteins indeed have similar roles as Ice2 remains to be tested. In contrast, Nem1, Spo7, and Pah1 are evolutionarily conserved (Han *et al*, 2012). The mammalian Pah1 orthologs lipin‐1/2/3 are phospho‐regulated in a similar manner as Pah1 and function in lipid storage in mice and humans (Harris & Finck, 2011; Zhang & Reue, 2017). Like in yeast, removal of lipins in protozoa, plants, worms, and flies causes ER expansion (Golden *et al*, 2009; Eastmond *et al*, 2010; Bahmanyar *et al*, 2014; Grillet *et al*, 2016; Pillai *et al*, 2017). Similarly, removal of the Nem1 ortholog CTDNEP1 causes ER expansion in human tissue culture cells (preprint: Merta *et al*, 2021). However, while both yeast and metazoa use the CDP‐DAG pathway to synthesize phosphatidylinositol, metazoa mainly generate phosphatidylcholine and phosphatidylethanolamine through the Kennedy pathway (Vance, 2015). The Kennedy pathway uses DAG as a precursor for phospholipids. Therefore, DAG is a precursor for both LD and membrane biogenesis, seemingly excluding the possibility that the balance between DAG and PA could determine whether LD or ER membrane biogenesis is favored. This incongruence may be resolved by the finding that the rate‐limiting enzyme for phosphatidylcholine synthesis by the Kennedy pathway, CCT, is activated by PA, be it by direct allosteric regulation as in *A. thaliana* or by more indirect means as in mice (Craddock *et al*, 2015; Zhang *et al*, 2019). Thus, accumulation of PA may favor conversion of DAG into phosphatidylcholine, thereby drawing it away from conversion into TAG and deposition in lipid droplets (Jacquemyn *et al*, 2017). This model, while speculative, raises the unifying possibility that lipins inversely govern LD and ER membrane biogenesis in all eukaryotes.

Lipins act at the ER but are also found at LDs, mitochondria, endosomes, and inside the nucleus (Zhang & Reue, 2017). Different organelles may use distinct mechanisms to recruit lipins and possess different regulators of lipin activity. Interestingly, *ICE2* overexpression expands the peripheral ER but does not obviously alter the morphology of the nucleus (Fig 9A). This is in contrast to deletion of *PAH1*, which leads to both peripheral ER expansion and nuclear membrane proliferation (Santos‐Rosa *et al*, 2005). There are distinct intra‐ and extranuclear pools of Pah1 (Romanauska & Köhler, 2018), so it appears possible that the intranuclear pool of Pah1 is responsible for maintaining proper nuclear morphology and is controlled in ways that do not involve Ice2. Furthermore, recent work in flies showed that the AAA‐type ATPase Torsin specifically inhibits nuclear lipin by removing the Nem1 ortholog CTDNEP1 from the nuclear envelope (Jacquemyn *et al*, 2021). Thus, organelle‐specific regulators of lipins may be important determinants of local lipin activity.

The regulation of lipid metabolism determines whether lipids are consumed, used to build membranes, or stored. Elucidating how this decision is made will yield a deeper understanding of differentiation processes, for example, the massive ER expansion during plasma cell development or the huge increase in lipid droplet abundance during adipogenesis. Moreover, it may enable therapeutic intervention in diseases associated with ER overload and aberrant lipid metabolism, such as diabetes and obesity. Besides organelle biogenesis, lipin activity impacts autophagy, axon regeneration, myopathy, dystonia, and neurodegeneration (Zhang *et al*, 2014; Grillet *et al*, 2016; Zhang & Reue, 2017; Fanning *et al*, 2019; Yang *et al*, 2019; Schäfer *et al*, 2020). Further unraveling the regulation of lipin will therefore have implications for a large variety of cellular processes and associated diseases.

## Materials and Methods

### Plasmids

Plasmids used in this study are listed in Appendix Table S1. To generate pNH605‐P_ADH1_‐GEM‐P_GAL1_‐ino2(L119A), the *ino2(L119A)* sequence was subcloned from pRS415_MET25_‐ino2(L119A) into pRS416‐P_GAL1_. The P_GAL1_‐ino2(L119A)‐T_CYC1_ cassette was then transferred into pNH605‐P_ADH1_‐GEM. Similarly, pRS306‐P_ADH1_‐GEM‐P_GAL1_‐ino2(L119A) was generated by transferring the P_GAL1_‐ino2(L119A) cassette from pRS416‐P_GAL1_‐ino2(L119A) into pRS306‐P_ADH1_‐GEM. To generate pFA6a‐mNeon‐HIS3MX6, mNeonGreen (Shaner *et al*, 2013) was amplified from pFA6a‐mNeon‐kanMX4 and inserted into pFA6a‐GFP(S65T)‐HISMX6, replacing GFP(S65T). To generate pFA6a‐mScarlet‐kanMX6, mScarlet (Bindels *et al*, 2017) was amplified from pFA6a‐mScarlet‐kanMX4 and inserted into pFA6a‐GFP(S65T)‐kanMX6, replacing GFP(S65T). To generate pRS415‐P_ADH1_‐Ice2, the *ICE2* coding sequence was amplified from yeast W303 genomic DNA and inserted into pRS415‐P_ADH1_. To generate plasmids encoding HA‐tagged Pah1, YCplac111‐Pah1‐PrtA was linearized by inverse PCR and re‐ligated using the NEBuilder HiFi DNA assembly mix (New England Biolabs, Ipswitch, Massachusetts) so that the Protein A sequence was replaced by a triple HA sequence. The segment of the *pah1(7A)* sequence containing the seven alanine substitutions was amplified from YCPlac111‐pah1(7A)‐PrtA and inserted into YCPlac111‐Pah1‐3HA to yield YCplac111‐pah1(7A)‐3HA.

### Yeast strain generation and growth

Strains used in this study are listed in Appendix Table S2. Strains generated for screening procedures were in the S288C background. All other strains were in the W303 background. Chromosomal modifications were introduced using PCR products or linearized plasmids (Longtine *et al*, 1998; Janke *et al*, 2004). The marker‐free strains SSY2836 and SSY2837 were derived from SSY2809 by transformation with the Pah1‐3HA or the pah1(7A)‐3HA sequence amplified from plasmids pSS1045 or pSS1047 followed by counterselection on 5‐fluoroorotic acid.

Strains were grown at 30°C on YPD, SCD‐MSG, or SCD medium as indicated. YPD medium consisted of 1% yeast extract (Becton Dickinson, Heidelberg, Germany), 2% peptone (Becton Dickinson), and 2% glucose (Merck, Darmstadt, Germany). SCD‐MSG medium consisted of 0.17% yeast nitrogen base without amino acids and ammonium sulfate (Formedium, Norfolk, UK), 0.1% monosodium glutamate, amino acids, and 2% glucose. SCD medium consisted of 0.7% yeast nitrogen base without amino acids (Sigma, Taufkirchen, Germany), amino acids, and 2% glucose.

### Light microscopy

Precultures were grown in liquid SCD medium during the day, diluted into fresh medium, and grown overnight for 16 h so that they reached mid log phase (OD_600_ = 0.5–1). For induction of the ER biogenesis system, overnight cultures were diluted to OD_600_ = 0.05 in fresh medium and treated with the indicated concentrations of ß‐estradiol (Sigma) for up to 6 h. For DTT treatment, overnight cultures were diluted to OD_600_ = 0.1 and incubated with 8 mM DTT (Roche, Mannheim, Germany) for up to 2 h. For staining LDs, cells in mid log phase were harvested from 1 ml of culture, resuspended in 20 μl 50 μM monodansylpentane (Biozol, Munich, Germany), incubated at room temperature for 10 min and washed once with water. Immediately before imaging, cells were harvested by centrifugation, mounted on coverslips, and covered with a 1% (w/v) agarose pad.

Images were acquired with a DMi8 inverted microscope (Leica, Wetzlar, Germany) equipped with a CSU‐X1 spinning‐disk confocal scanning unit (Yokogawa, Musashino, Japan) and an ORCA‐Flash 4.0 LT camera (Hamamatsu, Hamamatsu, Japan). A HC PL APO 63x/1.40‐0.60 or a HC PL APO 100×/1.4 CS2 oil objective lens (Leica) was used. The images shown in Fig EV4D and those used for the quantification in Fig 7F were acquired with an IX81 inverted microscope (Olympus, Hamburg, Germany) equipped with an ORCA‐R2 camera (Hamamatsu) and a PL APO 100x/1.45 oil DIC objective lens (Olympus). For the quantification in Fig 7F, Z‐stacks were acquired consisting of five optical slices spaced 1 μm apart. The percentage of cells with Nem1 or Spo7 puncta was determined from maximum intensity projections, and at least 400 cells were analyzed per condition in each biological replicate.

### UPR assays

UPR activity was measured by flow cytometry as described (Schmidt *et al*, 2019). Cells expressing cytosolic BFP and either the 4xUPRE‐GFP transcriptional UPR reporter or the *HAC1* mRNA splicing reporter (Jonikas *et al*, 2009; Pincus *et al*, 2010) were grown to mid log phase and treated with 800 nM estradiol, 8 mM DTT, or 1 μg/ml tunicamycin (Merck). Fluorescence was measured with a FACS Canto flow cytometer (BD Biosciences, Franklin Lakes, New Jersey) equipped with a high‐throughput sampler. Background autofluorescence was determined with identically grown isogenic control strains not harboring the UPR reporter. Background‐subtracted GFP fluorescence was divided by BFP fluorescence to account for differences in protein translation capacity. GFP/BFP ratios were normalized to untreated wild‐type cells.

### Cell lysis and western blotting

For standard Western blotting, cells were harvested by centrifugation, washed once with water, resuspended in 50 mM HEPES pH 7.5 containing 0.5 mM EDTA, 1 mM PMSF, and protease inhibitors (cOmplete, Roche), and disrupted by bead beating with a FastPrep 24 (MP Biomedicals). SDS was added to 1.5% (w/v), and proteins were solubilized by incubation at 65°C for 5 min. Equal protein amounts, as determined with the BCA assay kit (Pierce, Thermo Fisher Scientific, Waltham, Massachusetts), were resolved by SDS–PAGE and transferred onto nitrocellulose membranes. Membranes were probed with primary and HRP‐coupled secondary antibodies and developed with homemade ECL or SuperSignal West Femto maximum sensitivity substrate (Thermo Fisher). Chemiluminescence was detected with an ImageQuant LAS 4000 imaging system (GE Healthcare, Chalfont St Giles, UK). Primary antibodies were rabbit anti‐Sec63 (Feldheim *et al*, 1992), mouse anti‐mCherry 1C51 (Abcam, Cambridge, UK), rabbit anti‐Sec61 (Schuck *et al*, 2009), mouse anti‐Pgk1 22C5 (Abcam), rat anti‐HA 3F10 (Roche), mouse anti‐Dpm1 5C5A7 (Invitrogen, Thermo Fisher Scientific), and mouse anti‐FLAG M2 (Sigma).

For Phos‐tag PAGE of total cell lysates, cells were lysed by alkaline extraction and incubation in a modified SDS sample buffer at 65°C for 3 min (Kushnirov, 2000). Proteins were resolved on zinc‐containing Phos‐tag gels according to Nagy *et al*, 2018, with minor modifications. Gels contained 8% acrylamide/bisacrylamide (29:1), 25 μM Phos‐tag acrylamide (Fujifilm Wako Chemicals, Neuss, Germany), and 50 μM ZnCl_2_ in 350 mM Bis–Tris–HCl pH 6.8 and were run at 200 V at room temperature. An alternative, but equivalent, protocol was used for samples from *in vitro* assays. Samples were combined with 4× regular SDS sample buffer to adjust sample buffer concentration to 1×, incubated at 65°C for 5 min, and resolved on 8% gels containing 50 μM Phos‐tag acrylamide and 100 μM MnCl_2_ in 400 mM Tris–HCl pH 8.8. These gels were run at 80 V at 4°C for 20 min, followed by running at 15 mA/gel for 5 h. To remove metal ions, gels were washed 3 × 10 min with transfer buffer (25 mM Tris–HCl, 192 mM glycine, 20% ethanol) containing 1 mM EDTA and 2 × 20 min with transfer buffer. Blotting was done at 100 V at 4°C for 3 h, and blots were developed as above.

### Construction of the diploid ER marker knockout library

Using strains SSY2589 and SSY2590, the ER marker proteins Sec63‐mNeon and Rtn1‐mCherry and the GEM‐P_GAL1_‐ino2* cassette were integrated into a yeast knockout collection through modified SGA methodology (Giaever *et al*, 2002; Tong & Boone, 2007). SSY2589 and SSY2590 were independently mated to the knockout collection on YPD medium using a Singer RoToR robot. For each library, diploids were selected on SCD‐MSG lacking uracil and containing G418. Cells were pinned onto enriched sporulation medium (1% potassium acetate, 0.1% yeast extract, 0.05% glucose, 0.01% amino acid supplement consisting of only histidine, leucine, lysine, and uracil) and kept at 23°C for 5 days. Haploids were selected by two rounds of pinning onto SCD‐MSG lacking histidine/arginine/lysine with canavanine and thialysine or SCD‐MSG lacking leucine/arginine/lysine with canavanine and thialysine to select for MATa (Sec63‐mNeon library) and MATα (Rtn1‐mCherry library) cells, respectively. Haploid cells harboring the required markers were selected by sequential pinning onto appropriate media. The two libraries were then mated together, and diploids were selected on YPD containing nourseothricin and hygromycin to generate the final library with the genotype: xxx∆::kan/xxx∆::kan SEC63‐mNeon::HIS3/SEC63 RTN1‐mCherry::nat/RTN1 can1∆::GEM‐P_GAL1_‐ino2*‐URA3/can1∆::P_STE2_‐HIS3 lyp1∆::GEM‐P_GAL1_‐ino2*‐URA3/lyp1∆::P_STE3_‐LEU2 his3∆::P_GPD_‐TagBFP‐hph/his3∆0. This diploid library afforded two benefits compared with a haploid library. The larger size of diploid cells facilitated acquisition of informative images and the fact that cells were heterozygous for the fluorescently tagged ER marker proteins reduced the risk that specious phenotypes arose from impaired Sec63 or Rtn1 function.

### Automated microscopy

Cells were grown to saturation overnight in 100 μl SCD medium in regular 96‐well microtiter plates. Prior to imaging, 7 μl of culture was transferred into 1 ml fresh SCD medium containing 800 nM estradiol and grown for 5 h in 96 deep‐well microtiter plates to reach logarithmic growth phase. One‐hundred microliters of each sample was transferred into 96‐well glass‐bottomed microtiter plates (Brooks Life Sciences, Chelmsford, Massachusetts) coated with concanavalin A and allowed to attach. Medium was refreshed after 1 h to remove non‐attached cells. Samples were imaged with a Nikon Ti‐E wide‐field microscope equipped with a motorized stage, a Nikon perfect focus system, a Flash4 Hamamatsu sCMOS camera, and a 60×/1.49 oil immersion lens. For each sample, two fields of view were acquired consisting of five optical slices spaced 1 μm apart. Untreated wild‐type control strains were included in duplicate on each plate as a reference for unexpanded ER.

### Automated cell segmentation and ER size measurement

Image analysis was done in MATLAB using custom scripts. Initial cell objects were identified based on the cytoplasmic BFP. The best overall mid section image was selected by assessing the standard deviation in the BFP image, which is highest when the image is in focus. Next, fast Fourier transformation and bandpass filter were used to enhance contrast at the cell border. A Frangi filter (based on the implementation by D.J. Kroon, “Hessian based Frangi Vesselness filter”, MATLAB Central File Exchange. Retrieved May 2017) followed by Otsu thresholding was then used to generate a mask of apparent cell borders. Morphological opening followed by a minimum size filter was used to remove false labeling created by yeast vacuoles. The resulting image highlighted the cell borders. Because the borders of many cells touched each other, the internal space was used to identify and separate the individual cell objects. The intensity of these initial cell objects was measured, and only objects brighter than two median absolute deviations below the median were kept. Finally, any remaining touching cells, including connected mother cells and buds, were separated by water shedding. The segmentation of individual cell objects thus obtained was then optimized to generate more accurate cell boundaries and peripheral ER segmentation. For this, cells were cropped and the best mid section was reassessed on a per cell basis using the standard deviation of the Rtn1‐mCherry image. The BFP images were re‐segmented using the above procedure based on the new mid section. To accurately define the cell periphery for image quantification, object borders were expanded but contained within watershed boundaries. The ER was segmented in both the Sec63‐mNeon and Rtn1‐mCherry images using a Frangi tubeness filter. A more accurate cell border was defined by fitting a minimum volume ellipse (based on the implementation by N. Moshtagh, “Minimum Volume Enclosing Ellipsoid”, MATLAB Central File Exchange. Retrieved July 2017) to the combined masks of the segmented ER. Based on this segmentation, cell area, mean Sec63‐mNeon and Rtn1‐mCherry fluorescence, and cell roundness were calculated. An area of five pixels from the border was used to define the cell periphery area. Segmented ER falling within this area was used to define the peripheral ER area. From this, peripheral ER size (peripheral ER area divided by cell periphery area), ER profile size (mean area of ER profiles divided by cell periphery area), and number of ER gaps (number of gaps in the peripheral ER mask per micrometer cell periphery length) were calculated. Finally, to remove false cell objects, poorly segmented and dead cells, all of these measurements were used to limit the cell population to values within 2.5 standard deviations of the population mean. On average, 248 cells were analyzed per mutant, with the minimum being 25.

### Visual ER morphology analysis

Images were assessed visually using a custom image viewer application made in MATLAB. Segmented cells were arrayed in montages displaying 7 × 15 cells at a time. ER morphologies were independently annotated by two individuals with one or more of the following features: underexpanded, overexpanded, extended sheets, disorganized, and clustered. All strains with abnormal ER morphology were re‐imaged to ensure that the phenotype was robust.

### Computational ER expansion analysis

Since most gene deletions did not affect ER expansion, mutants from the same imaging plate served as a plate‐specific background population for comparison to individual deletion strains. Sec63‐mNeon intensity was used to define this background population and exclude the influence of extreme outliers. First, to remove plate effects, mNeon intensities were normalized by subtracting the plate means. Next, values were corrected for cell size (bigger cells being brighter) and cell count (densely crowded areas having an overall higher fluorescence) by local regression. Finally, the background population (BP) was defined for each plate as mutants that were within 1.5 standard deviations of the mean. To normalize the ER expansion measurements, a Z score was calculated as (sample − BP mean)/BP standard deviation, thereby removing plate effects. The time spent imaging each plate (approximately 50 min) was accounted for by correcting for well order by local regression. Similarly, cell density effects were corrected for by local regression against cell count. Scores were calculated separately for each field of view, and the maximum value was taken for each sample. False positives were removed by visual inspection, which was often caused by an out of focus field of view. Strains passing arbitrary thresholds of significance (Z score < −2 for total peripheral ER size and ER profile size, and > 2 for ER gaps) in at least two of the measurements and no overall morphology defects as defined above were re‐imaged in triplicate along with wild‐type control strains under both untreated and estradiol‐treated conditions. Images were inspected visually as a last filter to define the final list of strains with ER expansion defects.

### Semi‐automated cortical ER morphology quantification

For cell segmentation, bright field images were processed in Fiji to enhance the contrast of the cell periphery. For this, a Gaussian blur (sigma = 2) was applied to reduce image noise, followed by a scaling down of the image (*x* = *y* = 0.5) to reduce the effect of small details on cell segmentation. A tubeness filter (sigma = 1) was used to highlight cell borders, and images were scaled back up to the original resolution. Cells were segmented using CellX (Dimopoulos *et al*, 2014), and out of focus cells were removed manually. A user interface in MATLAB was then used to assist ER segmentation. The user inputs images of Sec63‐mNeon and Rtn1‐mCherry from cortical sections (background subtracted in Fiji using the rolling ball method with a radius of 50 pixels) and the cell segmentation file generated in CellX. Adjustable parameters controlled the segmentation of ER tubules and sheets for each image. These parameters were tubule/sheet radius, strength, and background. Manual fine‐tuning of these parameters was important to ensure consistent ER segmentation across images with different signal intensities. These parameters were set independently for Sec63‐mNeon and Rtn1‐mCherry images together with one additional parameter called “trimming factor”, which controls the detection of ER sheets. ER masks were calculated across entire images and assigned to individual cells based on the CellX segmentation. For each channel, the background (BG) levels were automatically calculated using Otsu thresholding and fine‐tuned by multiplying the threshold value by the “tubule BG” (Rtn1 channel) or “total ER BG” (Sec63 channel) adjustment parameters. A 3 × 3 median filter was applied to smoothen the images and reduce noise that is problematic for segmentation. Two rounds of segmentation were passed for each image channel (Sec63 or Rtn1) with one optimized for finding smaller features (tubules) and the other for larger features (sheets). First, convolution kernels were calculated for small and large features, respectively, defined as a ring of radius +1, where the radius is given in the “tubule radius” (small feature) or “sheet radius” (large feature) parameters. These convolution kernels were applied pixel‐wise to determine whether a pixel is brighter than the mean intensity of the surrounding ring of pixels. The strength of this filter was fine‐tuned by adjusting the “tubule strength” or “sheet strength” parameters. Additionally, segmented pixels had to be brighter than the background levels defined above. This procedure generated two segmented images per channel, which were combined to generate the “total ER” mask for that channel. To define which regions in each channel represented sheets or tubules, a morphological opening was applied, the degree of which was controlled by the trimming factor. Features that remained in the Sec63 segmentation mask after morphological opening were provisionally designated sheets, the remainder of the ER mask was designated tubules. Finally, areas that were sheet‐like in the Rtn1 segmentation mask and overlapped with the Sec63 mask were designated tubular clusters. Tubular clusters were subtracted from provisional sheets and added to the tubules to obtain the final designation of sheets and tubules. The median size measurements of each class were taken from the whole cell population.

### Quantitative real‐time PCR

Quantitative real‐time PCR was done exactly as described (Schmidt *et al*, 2019).

### Growth assays

For growth assays in liquid culture, cells were grown to saturation and diluted to OD_600_ = 0.05. For each strain, 500 μl culture was transferred into 48‐well plates in triplicate and absorbance at 600 nm in arbitrary units was measured at room temperature in 5‐min intervals for 24 h using a Tecan Infinite M1000 Pro plate reader. The area under the curve was calculated with the R package Growthcurver (Sprouffske & Wagner, 2016) and used as a measure for cell growth. For the growth assays shown in Fig 9C, cells were grown to mid log phase, diluted as above and absorbance was measured at 30°C in 5‐min intervals for 24 h using a Tecan Spark Cyto plate reader. This experimental regime ensured that *∆hac1* mutants grew as well as wild‐type cells. Growth assays on solid medium were done as described (Schuck *et al*, 2009) using SCD plates with and without 0.2 μg/ml tunicamycin. Plates were imaged after 1.5 days of growth at 30°C.

### Lipidomics

For each sample, approximately 2 × 10^8^ cells (10 ODs) were harvested from cultures grown to mid log phase and snap‐frozen in liquid nitrogen. Cells were disrupted with glass beads as above in 50 mM HEPES pH 7.5 containing 0.5 mM EDTA. Aliquots corresponding to 1,500–2,000 pmol total lipid were subjected to acidic Bligh and Dyer extractions, except for ergosteryl esters which were recovered by neutral extractions. Lipid extractions were performed in the presence of internal lipid standards. Sample amounts were adjusted to ensure that all lipid standard‐to‐lipid species ratios were in a linear range of quantification. Typically, the range of standard‐to‐species ratios were within a range of > 0.1 to < 10. Following this approach, a relative quantification of lipid species was performed. Lipid standard was added from a master mix containing 40 pmol d_7_‐PC mix (15:0/18:1‐d_7_, Avanti Polar Lipids), 25 pmol PI (17:0/20:4, Avanti Polar Lipids), 25 pmol PE and 15 pmol PS (14:1/14:1, 20:1/20:1, 22:1/22:1, semi‐synthesized as described in Özbalci *et al*, 2013), 20 pmol DAG (17:0/17:0, Larodan), 20 pmol TAG (D_7_‐TAG‐Mix, LM‐6000 / D5‐TAG 17:0,17:1,17:1, Avanti Polar Lipids), 20 pmol PA (PA 17:0/20:4, Avanti Polar Lipids), 5 pmol PG (14:1/14:1, 20:1/20:1, 22:1/22:1, semi‐synthesized as described in Özbalci *et al*, 2013), 40 pmol ergosteryl ester (15:0 and 19:0, semi‐synthesized as described in Gruber *et al*, 2018), and 20 pmol t‐Cer (18:0, Avanti Polar Lipids). Lipids recovered in the organic extraction phase were evaporated by a gentle stream of nitrogen. Prior to measurements, lipid extracts were dissolved in 10 mM ammonium acetate in methanol, diluted 1:10, and transferred into Eppendorf twin.tec 96‐well plates. Mass spectrometric measurements were performed in positive ion mode on an AB SCIEX QTRAP 6500+ mass spectrometer equipped with chip‐based (HD‐D ESI Chip, Advion Biosciences) nano‐electrospray infusion and ionization (TriVersa NanoMate, Advion Biosciences) as described (Özbalci *et al*, 2013). The following precursor ion scanning (PREC) and neutral loss scanning (NL) modes were used for the measurement of the various lipid classes: +PREC 184 (PC), +PREC282 (t‐Cer), +NL141 (PE), +NL185 (PS), +NL277 (PI), +NL189 (PG), +NL115 (PA), +NL77 (ergosterol), +PREC379 (ergosteryl ester). Ergosterol was quantified following derivatization to ergosterol acetate in the presence of 200 pmol of the internal standard (22E)‐Stigmasta‐5,7,22‐trien‐3‐beta‐ol (Aldrich, R202967) using 100 μl acetic anhydride/chloroform (1:12 v/v) overnight under argon atmosphere (Ejsing *et al*, 2009). Mass spectrometry settings: resolution: unit, low mass configuration; data accumulation: 400 MCA; curtain gas: 20; Interface heater temperature: 60; CAD: medium. Data evaluation was done using LipidView (Sciex) and ShinyLipids, a software developed in house.

### Pah1 dephosphorylation and phosphorylation assays

Microsomes were prepared from strains lacking *PEP4*, *PRB1*, *PAH1,* and additional genes as indicated. Removal of Pep4 and Prb1 was critical to prevent protein degradation during *in vitro* assays. Removal of Pah1 ensured that all microsome donor strains had the same ER morphology and avoided contamination of microsomes with Pah1. Five‐hundred ODs of cells were washed once with 100 mM Tris pH 9.4 containing 10 mM NaN_3_, incubated in 100 mM Tris pH 9.4 containing 10 mM NaN_3_ and 10 mM DTT at 30°C for 10 min, pelleted, resuspended to 20 OD/ml in spheroplast buffer (50 mM Tris pH 7.5, 1 M sorbitol), and treated with 0.2 U/OD zymolyase 100T (Biomol, Hamburg, Germany) at 30°C for 10 min. The resulting spheroplasts were washed three times with ice‐cold spheroplast buffer and resuspended to 100 OD/ml in hypo‐osmotic lysis buffer (50 mM HEPES pH 7.5, 2 mM EDTA, 200 mM sorbitol, 1 mM PMSF, protease inhibitors). Lysates were homogenized with 40 strokes of a Dounce homogenizer with a clearance of 0.01 ‐ 0.06 mm (Kimble Chase) and cleared twice by centrifugation at 3,000 xg at 4°C for 5 min. Total membranes were pelleted by centrifugation at 16,000 *g* at 4°C for 15 min, washed once with one volume of hypo‐osmotic lysis buffer, and resuspended in 200 μl membrane buffer (20 mM HEPES pH 6.8, 250 mM sorbitol, 150 mM potassium acetate, 5 mM magnesium acetate). To enrich ER‐derived microsomes, 200 μl membranes were loaded onto a 3.8 ml two‐step sucrose gradient (1.2/1.5 M sucrose in 20 mM HEPES pH 7.4, 50 mM potassium acetate, 1 mM DTT, 2 mM EDTA) and centrifuged at 150,000 *g* at 4°C for 1.5 h. Microsomes were collected from the 1.2/1.5 M sucrose interphase, diluted with five volumes membrane buffer, pelleted at 16,000 xg for 15 min, and washed once with one volume membrane buffer and once with one volume reaction buffer (50 mM Tris pH 7.1, 150 mM NaCl, 100 mM sodium acetate, 100 mM MgCl_2_, 1 mM DTT, 0.1 mg/ml BSA and protease inhibitors). Finally, microsomes were resuspended in 50 μl reaction buffer and dispersed by sonication.

To isolate Pah1‐FLAG, 10 ODs of cells were lysed as for standard Western blotting in 20 mM Tris pH 8 containing 150 mM KCl, 5 mM MgCl_2_, 1% Triton X‐100, 1 mM PMSF, protease inhibitors, and phosphatase inhibitors (PhosSTOP, Roche). Lysates were cleared by centrifugation at 16,000 *g* at 4°C for 2 min, and Pah1‐FLAG was immunoprecipitated with anti‐FLAG agarose beads (Sigma) at 4°C for 30 min. Beads were washed three times with the above lysis buffer and once with reaction buffer. Pah1‐FLAG was eluted twice with 50 μl 0.2 mg/ml FLAG peptide in reaction buffer, and eluates were pooled. Fifty μl microsomes from different strains were incubated with 50 μl immunoisolated Pah1‐FLAG from *Δnem1* or *Δice2* cells at 30°C for 30 min, and proteins were resolved by Phos‐tag PAGE and analyzed by Western blotting.

### Co‐immunoprecipitation

One hundred ODs of cells were spheroplasted, lysed, and homogenized as above. Lysates were cleared at 500 *g* at 4°C for 2 min and incubated with 0.5% Triton X‐100 at 4°C for 30 min, and insoluble material was removed at 100,000 *g* for 30 min. The supernatant (approximately 500 μg total protein) was incubated with anti‐FLAG agarose beads for 30 min to precipitate Spo7‐FLAG or Nem1‐FLAG. Beads were washed twice with hypo‐osmotic lysis buffer containing 0.5% Triton X‐100 and once with lysis buffer without detergent. Bound protein was eluted with SDS sample buffer at 65°C for 5 min and analyzed by Western blotting. For comparison, 15 μg cell lysate was analyzed in parallel, which corresponded to 3% of the protein amount used as input for immunoprecipitation.

### Proximity‐dependent biotinylation assay

Cells were harvested and lysed as for standard Western blotting but in SDS/Triton buffer (50 mM Tris pH 7.5, 0.4% SDS, 2% Triton X‐100, 150 mM NaCl, 5 mM EDTA, 1 mM DTT, protease inhibitors). Biotinylated proteins were captured with streptavidin agarose beads (Thermo Fisher) at 4°C for 1 h. Beads were washed twice with SDS/Triton buffer at 4°C for 5 min, twice with 10% SDS at room temperature for 5 min, twice with 50 mM HEPES pH 7.4 containing 500 mM NaCl, 1 mM EDTA, 1% Triton X‐100 and 0.1% sodium deoxycholate, and twice with 50 mM Tris pH 7.5 containing 50 mM NaCl and 0.1% Triton X‐100. Bound protein was eluted with SDS–PAGE sample buffer containing 2 mM biotin at 65°C for 10 min and analyzed by Western blotting.

### Experimental design

At least three biological replicates were done for experiments with quantitative read‐outs. Repetitions were considered biological replicates if they were initiated from independent liquid cultures of yeast strains and performed on different days. Microscopic images were anonymized with the “Blind Analysis Tools” plug‐in in ImageJ (https://imagej.net/plugins/blind‐analysis‐tools) before visual analysis to prevent user bias. For each experiment, the number of biological replicates (*n*), mean and standard error of the mean (s.e.m.) are reported in the figure legends. Statistical significance was tested with a two‐tailed Student's *t*‐test assuming equal variance. Exceptions were the tests against the normalized values for wild‐type cells in Figs 1C and 3D, and 4G, and EV2A–C, for which a two‐tailed Student's *t*‐test with unequal variance was applied.

## Author contributions

PWB, DP, and SS conceptualized the study; PWB involved in formal analysis; PWB, CL, OP, DP, and GR investigated the study; PWB provided software; BB and SS supervised the study; DP and SS wrote—original draft; all authors wrote—review and editing.

## Conflict of interest

The authors declare that they have no conflict of interest.

## Supporting information



Appendix

Expanded View Figures PDF

Table EV1

Dataset EV1

Source Data for Expanded View

Source Data for Figure 1

Source Data for Figure 2

Source Data for Figure 3

Source Data for Figure 4

Source Data for Figure 5

Source Data for Figure 6

Source Data for Figure 7

Source Data for Figure 8

Source Data for Figure 9

## Data Availability

The computer script for cortical ER morphology quantification is available at GitHub (https://github.com/SchuckLab/ClassifiER).
