# Supplementary material for: Ice2 promotes ER membrane biogenesis in yeast by inhibiting the conserved lipin phosphatase complex
Source: EMBO J. 2021 Oct 6;40(22):e107958. doi: 10.15252/embj.2021107958 (PMC8591542; doi:10.15252/embj.2021107958)
Supplement: Supplementary file 1 — Appendix [file EMBJ-40-e107958-s002.docx]

**TABLE OF CONTENTS**

Appendix Table S1 – Plasmids used in this study

Appendix Table S2 – Yeast strains used in this study

**Table S1. Plasmids used in this study.** GEM = GAL4DBD-EstR-Msn2TAD.

| Plasmid | Alias | Source | |
| --- | --- | --- | --- |
| pRS415-P_MET25_-ino2(L119A) | pSS108 | Heyken et al, 2005 | |
| pRS416-P_GAL1_ | pSS031 | Mumberg et al, 1994 | |
| pRS415-P_GAL1_-ino2(L119A) | pSS448 | this study | |
| pNH605-P_ADH1_-GEM | pDEP151 | David Pincus | |
| pNH605-P_ADH1_-GEM-P_GAL1_ | pSS474 | Schmidt et al, 2019 | |
| pNH605-P_ADH1_-GEM-P_GAL1_-ino2(L119A) | pSS475 | this study | |
| pRS416-P_GAL1_-ino2(L119A) | pSS448 | this study | |
| pRS306-P_ADH1_-GEM | pDEP001 | Pincus et al, 2014 | |
| pRS306-P_ADH1_-GEM-P_GAL1_ | pSS476 | Schmidt et al, 2019 | |
| pRS306-P_ADH1_-GEM-P_GAL1_-ino2(L119A) | pSS477 | this study | |
| pRS303H-P_GPD_-TagBFP | pMaM245 | Szoradi et al, 2018 | |
| pFA6a-mNeon-kanMX4 | pMaM375 | Michael Knop | |
| pFA6a-GFP(S65T)-HISMX6 | pSS039 | Longtine et al, 1998 | |
| pFA6a-mNeon-kanMX6 | pSS445 | Schäfer et al, 2020 | |
| pFA6a-mNeon-HIS3MX6 | pSS447 | this study | |
| pFA6a-mScarlet-kanMX4 | pSS913 | Michael Knop | |
| pFA6a-mScarlet-kanMX6 | pSS917 | this study | |
| pRS415-P_ADH1_ | pSS022 | Mumberg et al, 1995 | |
| pRS415-P_ADH1_-Ice2 | pSS761 | this study | |
| pRS304-4xUPRE-GFP | pDEP017 | Pincus et al, 2010 | |
| pRS305-HAC1-splicing-reporter | pDEP005 | Pincus et al, 2010 | |
| pFA6a-3xHA-HISMX6 | pSS042 | Longtine et al, 1998 | |
| pFA6a-3xFLAG-kanMX6 | pSS072 | this study | |
| pFA6a-TurboID-3myc-kanMX6 | pSS1061 | Larochelle et al, 2019 | |
| YCplac111-Pah1-PrtA | pSS1005 | O'Hara et al, 2006 | |
| YCplac111-pah1(7A)-PrtA | pSS1006 | O'Hara et al, 2006 | |
| YCplac111-Pah1-3HA | pSS1045 | this study | |
| YCplac111-pah1(7A)-3HA | pSS1047 | | this study |

**Table S2. Yeast strains used in this study.** mNeon = mNeonGreen. GEM = P_ADH1_-GAL4DBD-EstR-Msn2TAD. SR = splicing reporter.

| Strain | Relevant genotype | Source |
| --- | --- | --- |
| SSY122 | *ADE2 leu2-3,112 trp1-1 ura3-1 his3-11,15 MATa* | Szoradi, 2018 |
| SSY1404 | *Sec63-mNeon::kan Rtn1-mCherry::HIS3* | this study |
| SSY2328 | *Sec63-mNeon::kan Rtn1-mCherry::HIS3 leu2::GEM-LEU2* | this study |
| SSY1405 | *Sec63-mNeon::kan Rtn1-mCherry::HIS3 leu2::GEM-P_GAL1_-ino2(L119A)-LEU2* | this study |
| SSY2306 | *his3::P_GPD_-TagBFP-hph trp1::4xUPRE-GFP-TRP1* | this study |
| SSY2307 | *his3::P_GPD_-TagBFP-hph trp1::4xUPRE-GFP-TRP1 ura3::GEM-P_GAL1_-URA3* | this study |
| SSY2308 | *his3::P_GPD_-TagBFP-hph trp1::4xUPRE-GFP-TRP1 ura3::GEM-P_GAL1_-ino2(L119A)-URA3* | this study |
| SSY1607 | *Sec63-mNeon::kan Rtn1-mCherry::HIS3 leu2::GEM-P_GAL1_-ino2(L119A)-LEU2 opi1∆::hph* | this study |
| Y8205 | *can1∆::P_STE2_-Sp_his5 lyp1∆::P_STE3_-LEU2 his3∆1 leu2∆0 ura3∆0 MATα* | Tong, 2007 |
| SSY2589 | *Y8205 Sec63-mNeon::HIS3 his3::P_GPD_-TagBFP-hph can1::GEM-P_GAL1_-ino2(L119A)-URA3* | this study |
| SSY2590 | *Y8205 Rtn1-mCherry::nat lyp1::GEM-P_GAL1_-ino2(L119A)-URA3* | this study |
| SSY1603 | *Sec63-mNeon::kan Rtn1-mCherry::HIS3 leu2::GEM-P_GAL1_-ino2(L119A)-LEU2 ice2∆::hph* | this study |
| SSY2356 | *Sec63-mNeon::kan Rtn1-mCherry::HIS3 ice2∆::nat* | this study |
| SSY2595 | *Sec63-mNeon::kan Rtn1-mCherry::HIS3 opi1∆::hph* | this study |
| SSY2811 | *Sec63-mNeon::kan Rtn1-mCherry::HIS3 opi1∆::hph ice2∆::nat* | this study |
| SSY2312 | *his3::P_GPD_-TagBFP-hph trp1::4xUPRE-GFP-TRP1 ice2∆::nat* | this study |
| SSY2309 | *his3::P_GPD_-TagBFP-hph leu2::HAC1-SR-LEU2* | this study |
| SSY2313 | *his3::P_GPD_-TagBFP-hph leu2::HAC1-SR-LEU2 ∆ice2∆::nat* | this study |
| YMG1 | *ade2-1 leu2-3,112 trp1-1 ura3-1 his3-11,15 can1-100 MATa* | Velazquez, 2016 |
| SSY2228 | *YMG1 Sec63-mNeon::URA3 Rtn1-mCherry::kan* | this study |
| SSY2331 | *YMG1 Sec63-mNeon::URA3 Rtn1-mCherry::kan hac1∆::nat* | this study |
| SSY2314 | *his3::P_GPD_-TagBFP-hph trp1::4xUPRE-GFP-TRP1 hac1∆::nat* | this study |
| SSY2229 | *YMG1 Sec63-mNeon::URA3 Rtn1-mCherry::kan ice2∆::nat* | this study |
| YMG5 | *YMG1 dga1∆::TRP1 lro1∆::HIS3 are1∆::TRP1 are2∆::HIS3* | Velazquez, 2016 |
| SSY2256 | *YMG5 Sec63-mNeon::URA3 Rtn1-mCherry::kan* | this study |
| SSY2230 | *YMG5 Sec63-mNeon::URA3 Rtn1-mCherry::kan ice2∆::nat* | this study |
| SSY2598 | *YMG1 ADE2 Sec63-mNeon::URA3 Rtn1-mCherry::kan leu2::GEM-P_GAL1_-ino2(L119A)-LEU2* | this study |
| SSY2599 | *SSY2598 ice2∆::nat* | this study |
| SSY2600 | *YMG5 ADE2 Sec63-mNeon::URA3 Rtn1-mCherry::kan leu2::GEM-P_GAL1_-ino2(L119A)-LEU2* | this study |
| SSY2601 | *SSY2600 ice2∆::nat* | this study |
| SSY2482 | *Sec63-mNeon::kan Rtn1-mCherry::HIS3 nem1∆::hph* | this study |
| SSY2484 | *Sec63-mNeon::kan Rtn1-mCherry::HIS3 ice2∆::nat nem1∆::hph* | this study |
| SSY2481 | *Sec63-mNeon::kan Rtn1-mCherry::HIS3 spo7∆::hph* | this study |
| SSY2483 | *Sec63-mNeon::kan Rtn1-mCherry::HIS3 ice2∆::nat spo7∆::hph* | this study |
| SSY2807 | *Sec63-mNeon::kan Rtn1-mCherry::HIS3 pah1∆::hph* | this study |
| SSY2808 | *Sec63-mNeon::kan Rtn1-mCherry::HIS3 ice2∆::nat pah1∆::hph* | this study |
| SSY2592 | *Pah1-3HA::HIS3* | this study |
| SSY2593 | *Pah1-3HA::HIS3 ice2∆::nat* | this study |
| SSY2594 | *Pah1-3HA::HIS3 nem1∆::nat* | this study |
| SSY2718 | *Pah1-3HA::HIS3 ice2∆::nat nem1∆::hph* | this study |
| SSY3065 | *nem1Δ::nat Pah1-3FLAG::kan* | this study |
| SSY3053 | *pep4∆::TRP1 prb1∆::HIS3 pah1Δ::nat* | this study |
| SSY3074 | *pep4∆::TRP1 prb1∆::HIS3 pah1Δ::hph ice2Δ::nat* | this study |
| SSY3075 | *pep4∆::TRP1 prb1∆::HIS3 pah1Δ::hph nem1Δ::nat* | this study |
| SSY3095 | *pep4∆::TRP1 prb1∆::HIS3 pah1Δ::hph nem1Δ::nat ice2Δ::kan* | this study |
| SSY3140 | *Nem1-3HA::HIS3 pah1∆::hph* | this study |
| SSY3141 | *Nem1-3HA::HIS3 pah1∆::hph ice2∆::nat* | this study |
| SSY3096 | *ice2Δ::nat Pah1-3FLAG::kan* | this study |
| SSY2421 | *Ice2-3HA::HIS* | this study |
| SSY3183 | *Spo7-3FLAG::kan* | this study |
| SSY3184 | *Ice2-3HA::HIS3 Spo7-3FLAG::kan* | this study |
| SSY3197 | *Ice2-3HA::HIS3 Spo7-3FLAG::kan nem1∆::hph* | this study |
| SSY2913 | *Nem1-3HA::HIS3* | this study |
| SSY2914 | *Nem1-3HA::HIS3 ice2∆::nat* | this study |
| SSY2915 | *Nem1-3HA::HIS3 ura3::P_ADH1_-Ice2-LEU2* | this study |
| SSY2945 | *Nem1-3HA::HIS3 spo7∆::hph* | this study |
| SSY3195 | *Nem1-3FLAG::kan* | this study |
| SSY3196 | *Ice2-3HA::HIS3 Nem1-3FLAG::kan* | this study |
| SSY2978 | *Ice2-TurboID-3myc::kan* | this study |
| SSY2979 | *Ice2-TurboID-3myc::kan Pah1-3HA::HIS3* | this study |
| SSY3117 | *Ice2-TurboID-3myc::kan Pah1-3HA::HIS3 spo7∆::nat* | this study |
| SSY3118 | *Ice2-TurboID-3myc::kan Pah1-3HA::HIS3 nem1∆::nat* | this study |
| SSY2910 | *Spo7-3HA::HIS3* | this study |
| SSY2911 | *Spo7-3HA::HIS3 ice2∆::nat* | this study |
| SSY2912 | *Spo7-3HA::HIS3 ura3::P_ADH1_-Ice2-LEU2* | this study |
| SSY3244 | *Ice2-mScarlet::kan Spo7-mNeon::nat* | this study |
| SSY3245 | *Ice2-mScarlet::kan Nem1-mNeon::nat* | this study |
| SSY2916 | *ura3::Ptef-cherry-Ubc6-URA3 Spo7-neon::nat* | this study |
| SSY2917 | *ura3::Ptef-cherry-Ubc6-URA3 Nem1-neon::nat* | this study |
| SSY3238 | *ura3::Ptef-cherry-Ubc6-URA3 Spo7-neon::nat ice2Δ::hph* | this study |
| SSY3239 | *ura3::Ptef-cherry-Ubc6-URA3 Nem1-neon::nat ice2Δ::hph* | this study |
| SSY3318 | *Ice2-mScarlet::kan Sei1-neon::HIS* | this study |
| SSY2809 | *pah1∆::URA3* | this study |
| SSY2836 | *Pah1-3HA* | this study |
| SSY2837 | *pah1(7A)-3HA* | this study |
| SSY2841 | *Pah1-3HA Sec63-mNeon::kan Rtn1-mCherry::HIS3* | this study |
| SSY2842 | *pah1(7A)-3HA Sec63-mNeon::kan Rtn1-mCherry::HIS3* | this study |
| SSY2970 | *Pah1-3HA Sec63-mNeon::kan Rtn1-mCherry::HIS3 ice2Δ::nat* | this study |
| SSY2843 | *Pah1-3HA Sec63-mNeon::kan Rtn1-mCherry::HIS3 ura3::P_ADH1_-Ice2-LEU2* | this study |
| SSY2844 | *pah1(7A)-3HA Sec63-mNeon::kan Rtn1-mCherry::HIS3 ura3::P_ADH1_-Ice2-LEU2* | this study |
| SSY2588 | *Sec63-mNeon::kan Rtn1-mCherry::HIS3 ura3::Padh-Ice2-LEU2* | this study |
| SSY2596 | *Sec63-mNeon::kan Rtn1-mCherry::HIS3 ura3::Padh-Ice2-LEU2 opi1∆::hph* | this study |
| SSY2805 | *Sec63-mNeon::kan Rtn1-mCherry::HIS3 hac1∆::hph* | this study |
| SSY2806 | *Sec63-mNeon::kan Rtn1-mCherry::HIS3 ice2∆::nat hac1∆::hph* | this study |

References:

Larochelle M, Bergeron D, Arcand B, Bachand F (2019) Proximity‐dependent biotinylation mediated by TurboID to identify protein‐protein interaction networks in yeast. *J Cell Sci* 132: jcs232249

Mumberg D, Müller R, Funk M (1994) Regulatable promoters of *Saccharomyces cerevisiae*: comparison of transcriptional activity and their use for heterologous expression. *Nucleic Acids Res* 22: 5767–5768

Mumberg D, Müller R, Funk M (1995) Yeast vectors for the controlled expression of heterologous proteins in different genetic backgrounds. *Gene* 156: 119–122

Szoradi T, Schaeff K, Garcia‐Rivera EM, Itzhak DN, Schmidt RM, Bircham PW, Leiss K, Diaz‐Miyar J, Chen VK, Muzzey D *et al* (2018) SHRED is a regulatory cascade that reprograms Ubr1 substrate specificity for enhanced protein quality control during stress. *Mol Cell* 70: 1025–1037

Velázquez AP, Tatsuta T, Ghillebert R, Drescher I, Graef M (2016) Lipid droplet–mediated ER homeostasis regulates autophagy and cell survival during starvation. *J Cell Biol* 212: 621–631
