# Supplementary material for: Ice2 promotes ER membrane biogenesis in yeast by inhibiting the conserved lipin phosphatase complex
Source: EMBO J. 2021 Oct 6;40(22):e107958. doi: 10.15252/embj.2021107958 (PMC8591542; doi:10.15252/embj.2021107958)
Supplement: Supplementary file 2 — Expanded View Figures PDF [file EMBJ-40-e107958-s015.pdf]

Expanded View Figures

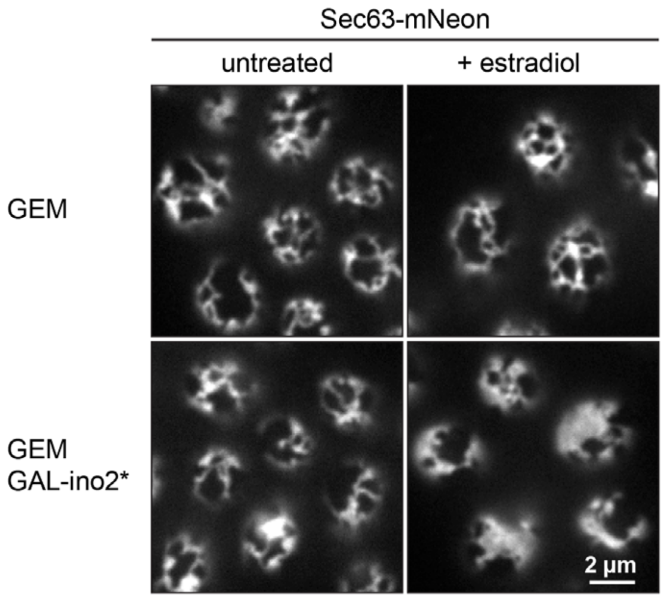

Figure EV1. An inducible system for ER membrane biogenesis.

Sec63-mNeon images of cortical sections of cells containing the estradiol-inducible artificial transcription factor GEM (SSY2328) and cells additionally containing ino2* under the control of the *GAL* promoter (SSY1405). Cells were untreated or treated with 800 nM estradiol for 6 h.

Source data are available online for this figure.

© 2021 The Authors

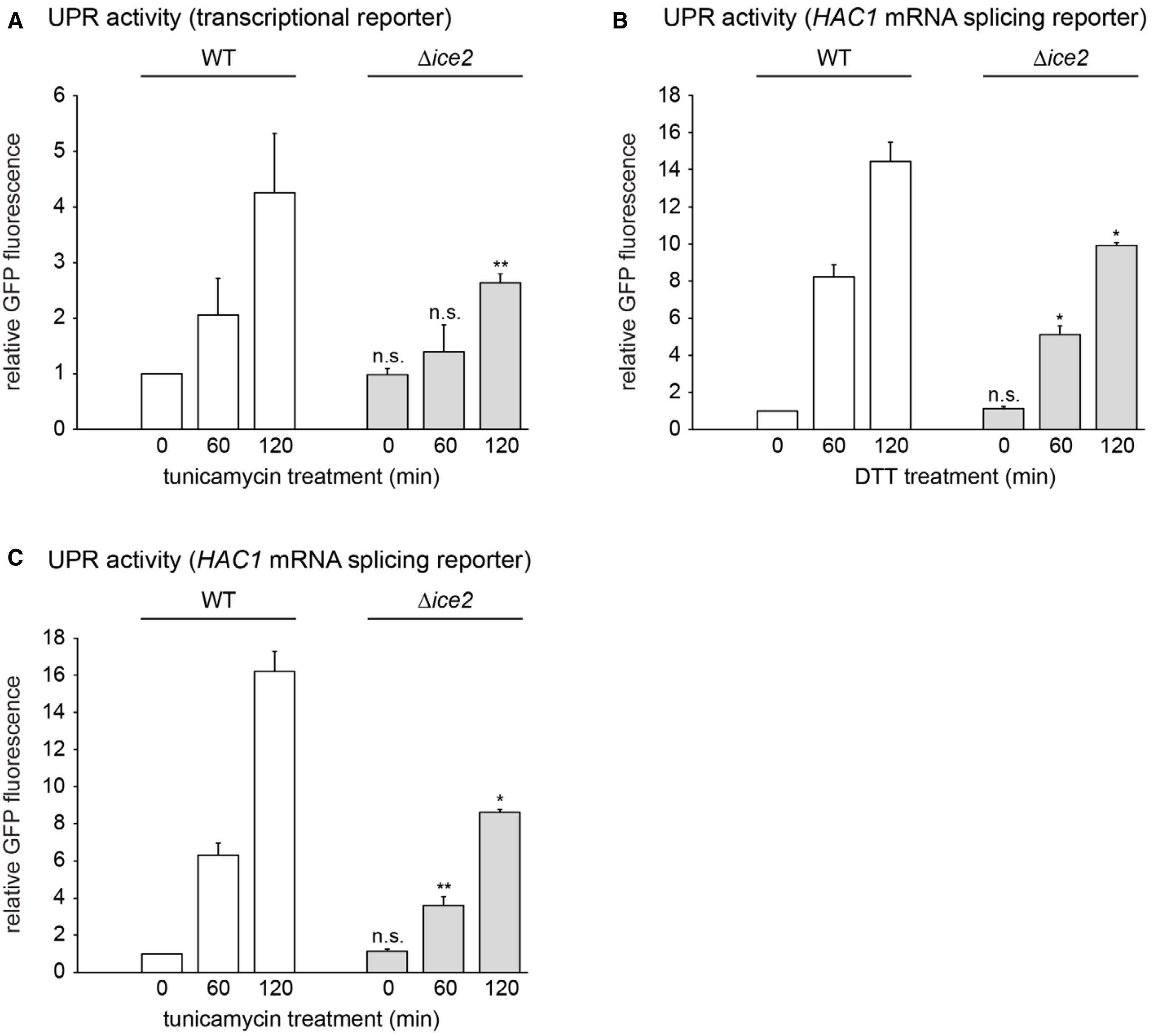

Figure EV2. Deletion of *ICE2* impairs UPR signaling during ER stress.

A Flow cytometric measurements of GFP levels of WT and *Δice2* cells containing the transcriptional UPR reporter (SSY2306, 2312). Cells were treated with 1 μg/ml tunicamycin for the times indicated. Data were normalized to untreated WT cells. Mean + s.e.m., $n = 3$ biological replicates. Asterisks indicate statistical significance compared with the corresponding value in WT cells, as judged by a two-tailed Student's *t*-test assuming equal variance. An exception was the test against the normalized value for WT cells, for which a two-tailed Student's *t*-test with unequal variance was applied. $^{**}P < 0.01$; n.s., not significant.

B, C Flow cytometric measurements of GFP levels of WT and *Δice2* cells containing the *HAC1* mRNA splicing reporter (SSY2309, 2313). Cells were treated with 8 mM DTT (B) or 1 μg/ml tunicamycin (C) for the times indicated. Data were normalized to untreated WT cells. Mean + s.e.m., $n = 3$ biological replicates. Asterisks indicate statistical significance compared with the corresponding value in WT cells, as judged by a two-tailed Student's *t*-test assuming equal variance. Exceptions were the tests against the normalized values for WT cells, for which a two-tailed Student's *t*-test with unequal variance was applied. $^{*}P < 0.05$; $^{**}P < 0.01$; n.s., not significant.

Source data are available online for this figure.

Figure EV3. Absence of lipid droplets has no effect on ER expansion in WT or Δ*ice2* cells.

A Growth assays of untreated WT, *Δice2*, *ΔLD*, and *ΔLD Δice2* cells (SSY2228, 2229, 2230, 2256). Numbers represent areas under the curves and serve as growth indices. Mean + s.e.m., $n = 3$ biological replicates. Δ*LD*, Δ*lipid droplet*.

B, C Quantification of peripheral ER structures in WT, *Δice2*, *ΔLD*, and *ΔLD Δice2* cells harboring the inducible system (SSY2598, 2599, 2600, 2601), which were untreated or treated with either 800 nM estradiol for 6 h (B) or 8 mM DTT for 1 h (C). Bars are the mean percentage of cell cortex covered by tubules (purple) or sheets (green), $n = 3$ biological replicates. Upper error bars are s.e.m. for the sum of tubules and sheets, and lower error bars are s.e.m. for sheets. Asterisks indicate statistical significance, as judged by a two-tailed Student's *t*-test assuming equal variance. *$P < 0.05$; **$P < 0.01$; n.s., not significant.

D Lipidomic analysis of WT, *Δice2*, *Δnem1*, *Δice2 Δnem1*, *Δspo7*, and *Δice2 Δspo7* cells (SSY1404, 2356, 2482, 2484, 2481, 2483). Mean + s.e.m., $n = 4$ biological replicates. Asterisks indicate statistical significance compared with WT cells, as judged by a two-tailed Student's *t*-test assuming equal variance. *$P < 0.05$; **$P < 0.01$. The data are the same as in Fig 5C and D but are shown as lipid-to-protein ratios in μg measured lipid per g total protein.

Source data are available online for this figure.

© 2021 The Authors

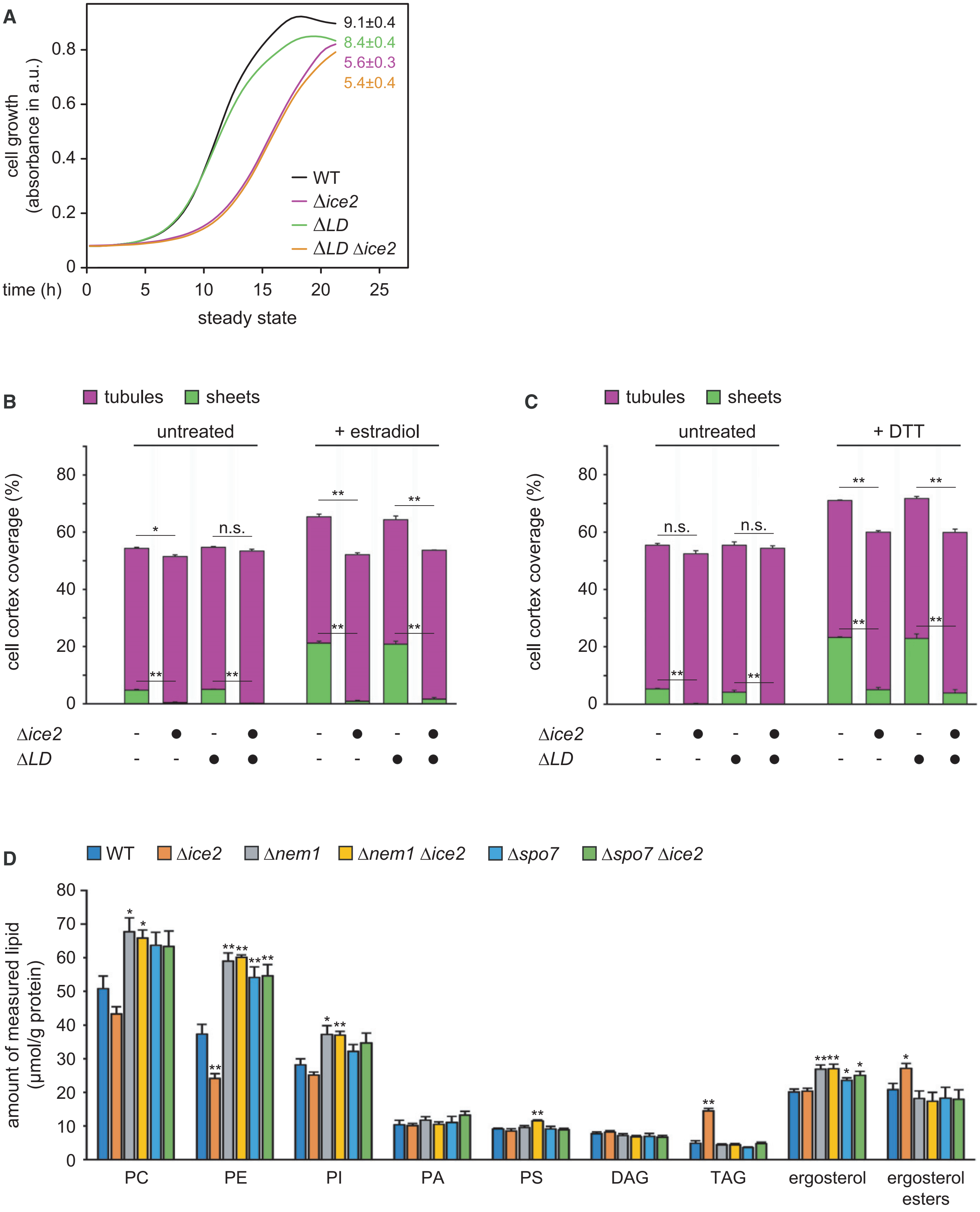
A
cell growth (absorbance in a.u.)
9.1±0.4
8.4±0.4
5.6±0.3
5.4±0.4
WT
Δice2
ΔLD
ΔLD Δice2
time (h)
steady state
B
tubules
sheets
untreated
+ estradiol
cell cortex coverage (%)
n.s.
Δice2
ΔLD
C
+ DTT
D
WT
Δice2
Δnem1
Δnem1 Δice2
Δspo7
Δspo7 Δice2
amount of measured lipid (µmol/g protein)
PC
PE
PI
PA
PS
DAG
TAG
ergosterol
ergosterol esters

Figure EV3.

Figure EV4. Abundance of Ice2, Spo7, and Nem1.

A Western blot of HA from total membranes prepared from WT, Δ*ice2*, and *ICE2*-overexpressing and Δ*spo7* cells containing Nem1-HA (SSY2913, 2914, 2915, 2945). An unspecific band served as a loading control.

B Western blot of HA from total cell membranes from WT cells (SSY122) and cells expressing Ice2-HA, Spo7-HA, or Nem1-HA (SSY2421, 2910, 2913). Dpm1 served as a loading control.

C Western blot of HA from total membranes prepared from WT cells (SSY122), WT, Δ*ice2*, and *ICE2*-overexpressing cells containing Spo7-HA (SSY2910, 2911, 2912), and WT, Δ*ice2*, and *ICE2*-overexpressing cells containing Nem1-HA (SSY2913, 2914, 2915). Sec61 served as a loading control. o/e, overexpression.

D Images of cells expressing endogenously tagged Ice2-mScarlet and Sei1-mNeon (SSY3318) and stained with monodansylpentane to highlight lipid droplets. Arrows indicate foci containing Ice2.

Source data are available online for this figure.

 © 2021 The Authors

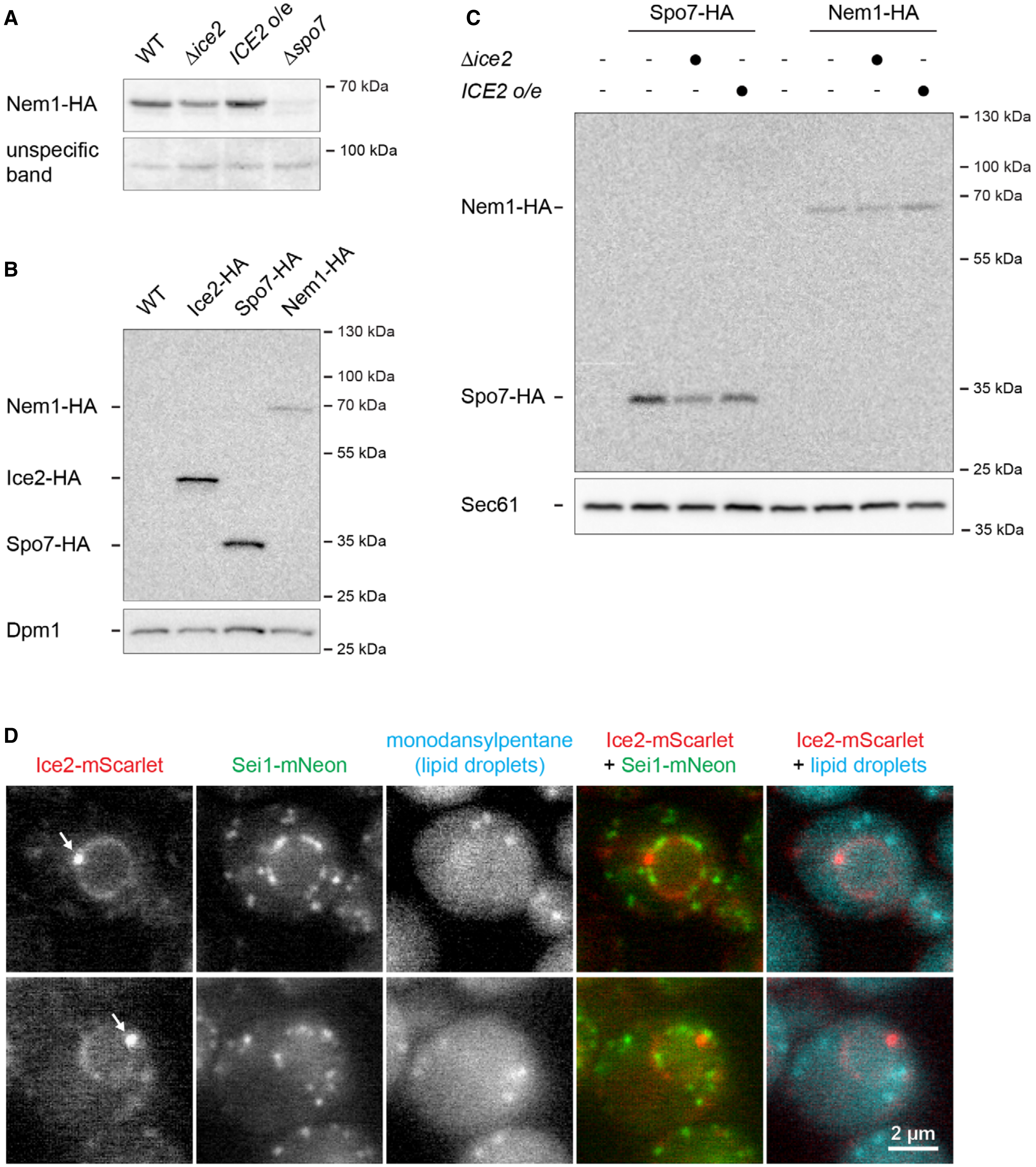
A
WT
Δice2
ICE2 o/e
Δspo7
Nem1-HA
unspecific band
70 kDa
100 kDa
B
WT
Ice2-HA
Spo7-HA
Nem1-HA
Nem1-HA
Ice2-HA
Spo7-HA
Dpm1
130 kDa
100 kDa
70 kDa
55 kDa
35 kDa
25 kDa
25 kDa
C
Spo7-HA
Nem1-HA
Δice2
ICE2 o/e
Nem1-HA
Spo7-HA
Sec61
130 kDa
100 kDa
70 kDa
55 kDa
35 kDa
25 kDa
35 kDa
D
Ice2-mScarlet
Sei1-mNeon
monodansylpentane (lipid droplets)
Ice2-mScarlet + Sei1-mNeon
Ice2-mScarlet + lipid droplets
2 µm

Figure EV4.

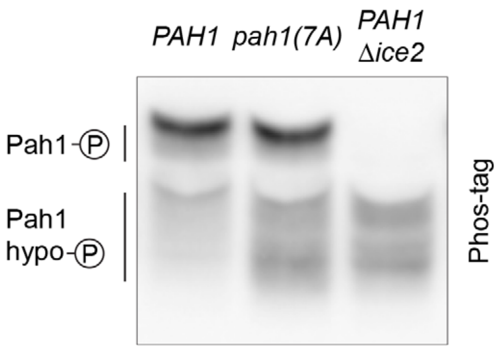

Figure EV5. Phosphorylation status of pah1(7A).

Western blot of HA from WT and Δ*ice2* cells in which *PAH1* was replaced with *PAH1-HA* or *pah1(7A)-HA* as indicated (SSY2841, SSY2842, SSY2970).

Source data are available online for this figure.

 © 2021 The Authors
