# Supplementary material for: Ice2 promotes ER membrane biogenesis in yeast by inhibiting the conserved lipin phosphatase complex
Source: EMBO J. 2021 Oct 6;40(22):e107958. doi: 10.15252/embj.2021107958 (PMC8591542; doi:10.15252/embj.2021107958)
Supplement: Supplementary file 5 — Source Data for Expanded View [file EMBJ-40-e107958-s001.zip › SourceData_EVFigs/EV4/EV4A.pdf]

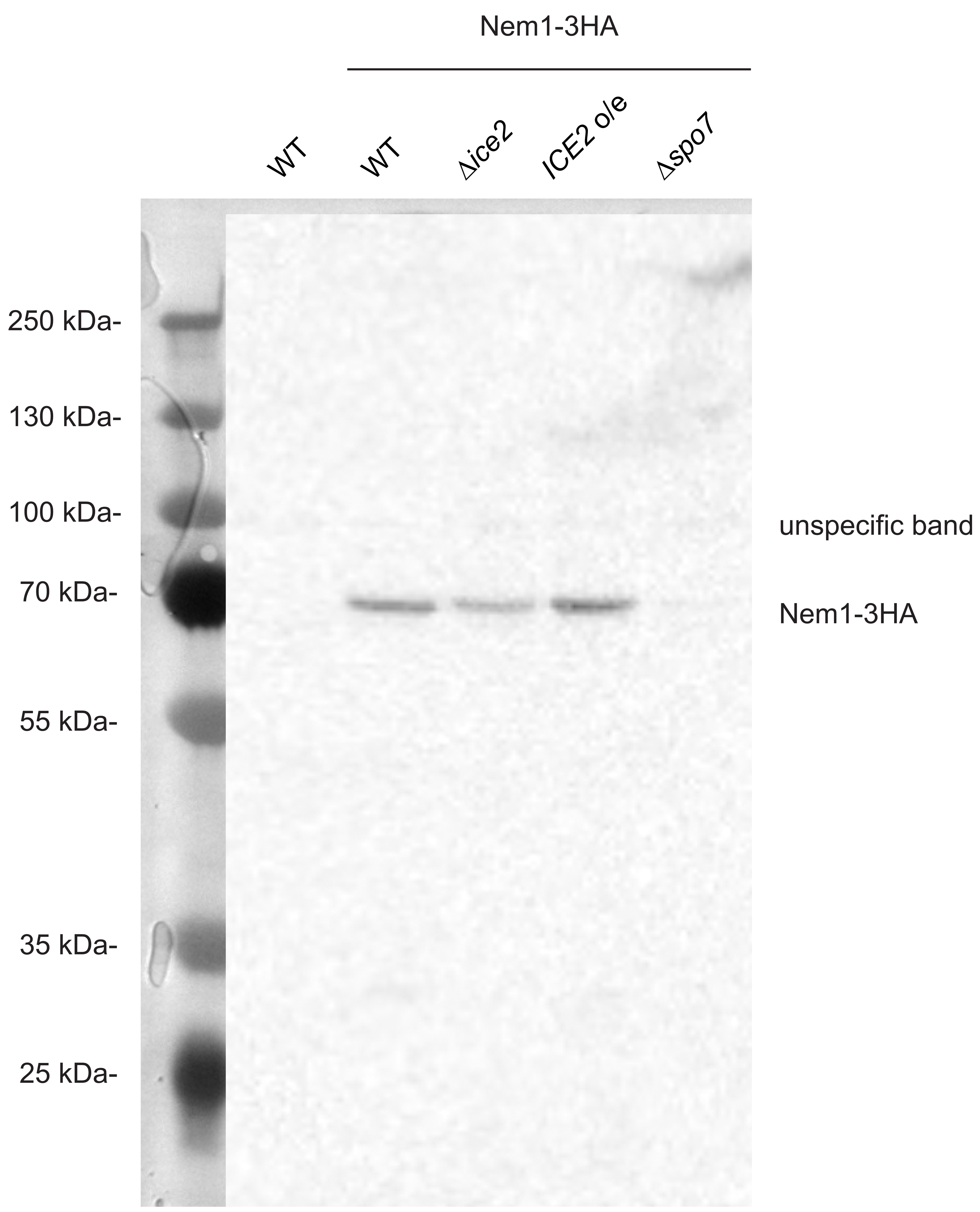

Note: lanes 2-5 were used for Figure EV4A. The unspecific band at around 100 kDa was used as a loading control. This band is barely visible without adjusting the intensity levels, see Figure EV4A.
