# Supplementary figures and images for: Ice2 promotes ER membrane biogenesis in yeast by inhibiting the conserved lipin phosphatase complex
Source: EMBO J. 2021 Oct 6;40(22):e107958. doi: 10.15252/embj.2021107958 (PMC8591542; doi:10.15252/embj.2021107958)

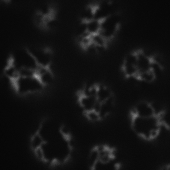

Supplement: Supplementary file 5 — Source Data for Expanded View [file EMBJ-40-e107958-s001.zip › SourceData_EVFigs/EV1/GEM untreated.tif]

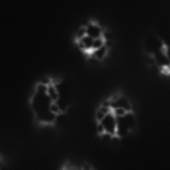

Supplement: Supplementary file 5 — Source Data for Expanded View [file EMBJ-40-e107958-s001.zip › SourceData_EVFigs/EV1/GEM estradiol.tif]

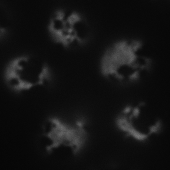

Supplement: Supplementary file 5 — Source Data for Expanded View [file EMBJ-40-e107958-s001.zip › SourceData_EVFigs/EV1/GEM GAL-ino2* estradiol.tif]

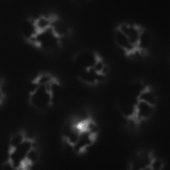

Supplement: Supplementary file 5 — Source Data for Expanded View [file EMBJ-40-e107958-s001.zip › SourceData_EVFigs/EV1/GEM GAL-ino2* untreated.tif]

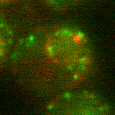

Supplement: Supplementary file 5 — Source Data for Expanded View [file EMBJ-40-e107958-s001.zip › SourceData_EVFigs/EV4/EV4D/bottom Ice2scarlet Sei1neon LDs merge red and green.tif]

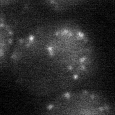

Supplement: Supplementary file 5 — Source Data for Expanded View [file EMBJ-40-e107958-s001.zip › SourceData_EVFigs/EV4/EV4D/bottom Ice2scarlet Sei1neon LDs green channel.tif]

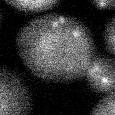

Supplement: Supplementary file 5 — Source Data for Expanded View [file EMBJ-40-e107958-s001.zip › SourceData_EVFigs/EV4/EV4D/top Ice2scarlet Sei1neon LDs blue channel.tif]

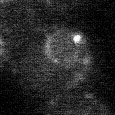

Supplement: Supplementary file 5 — Source Data for Expanded View [file EMBJ-40-e107958-s001.zip › SourceData_EVFigs/EV4/EV4D/bottom Ice2scarlet Sei1neon LDs red channel.tif]

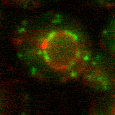

Supplement: Supplementary file 5 — Source Data for Expanded View [file EMBJ-40-e107958-s001.zip › SourceData_EVFigs/EV4/EV4D/top Ice2scarlet Sei1neon LDs merge red and green.tif]

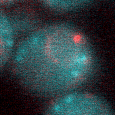

Supplement: Supplementary file 5 — Source Data for Expanded View [file EMBJ-40-e107958-s001.zip › SourceData_EVFigs/EV4/EV4D/bottom Ice2scarlet Sei1neon LDs merge red and blue.tif]

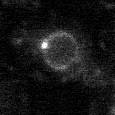

Supplement: Supplementary file 5 — Source Data for Expanded View [file EMBJ-40-e107958-s001.zip › SourceData_EVFigs/EV4/EV4D/top Ice2scarlet Sei1neon LDs red channel.tif]

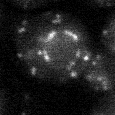

Supplement: Supplementary file 5 — Source Data for Expanded View [file EMBJ-40-e107958-s001.zip › SourceData_EVFigs/EV4/EV4D/top Ice2scarlet Sei1neon LDs green channel.tif]

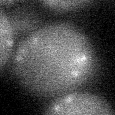

Supplement: Supplementary file 5 — Source Data for Expanded View [file EMBJ-40-e107958-s001.zip › SourceData_EVFigs/EV4/EV4D/bottom Ice2scarlet Sei1neon LDs blue channel.tif]

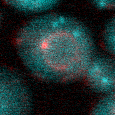

Supplement: Supplementary file 5 — Source Data for Expanded View [file EMBJ-40-e107958-s001.zip › SourceData_EVFigs/EV4/EV4D/top Ice2scarlet Sei1neon LDs merge red and blue.tif]

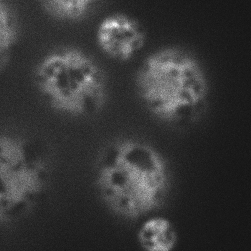

Supplement: Supplementary file 6 — Source Data for Figure 1 [file EMBJ-40-e107958-s010.zip › 1B/1B_estradiol_cortical.tif]

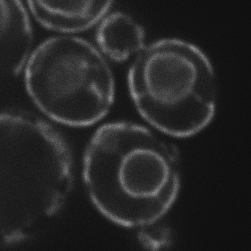

Supplement: Supplementary file 6 — Source Data for Figure 1 [file EMBJ-40-e107958-s010.zip › 1B/1B_estradiol_mid.tif]

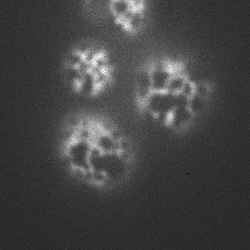

Supplement: Supplementary file 6 — Source Data for Figure 1 [file EMBJ-40-e107958-s010.zip › 1B/1B_untreated_cortical.tif]

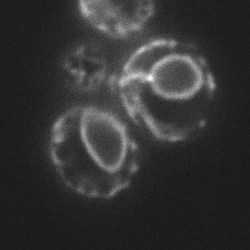

Supplement: Supplementary file 6 — Source Data for Figure 1 [file EMBJ-40-e107958-s010.zip › 1B/1B_untreated_mid.tif]

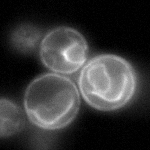

Supplement: Supplementary file 7 — Source Data for Figure 2 [file EMBJ-40-e107958-s007.zip › 2A/arv1.tif]

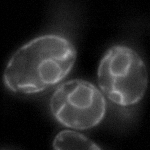

Supplement: Supplementary file 7 — Source Data for Figure 2 [file EMBJ-40-e107958-s007.zip › 2A/bre1.tif]

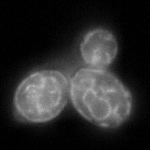

Supplement: Supplementary file 7 — Source Data for Figure 2 [file EMBJ-40-e107958-s007.zip › 2A/cog1.tif]

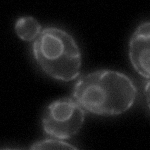

Supplement: Supplementary file 7 — Source Data for Figure 2 [file EMBJ-40-e107958-s007.zip › 2A/dgk1.tif]

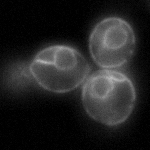

Supplement: Supplementary file 7 — Source Data for Figure 2 [file EMBJ-40-e107958-s007.zip › 2A/erd1.tif]

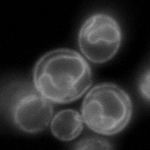

Supplement: Supplementary file 7 — Source Data for Figure 2 [file EMBJ-40-e107958-s007.zip › 2A/fyv6.tif]

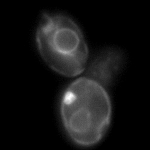

Supplement: Supplementary file 7 — Source Data for Figure 2 [file EMBJ-40-e107958-s007.zip › 2A/gga1.tif]

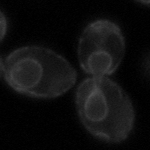

Supplement: Supplementary file 7 — Source Data for Figure 2 [file EMBJ-40-e107958-s007.zip › 2A/ice2.tif]

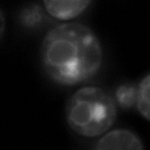

Supplement: Supplementary file 7 — Source Data for Figure 2 [file EMBJ-40-e107958-s007.zip › 2A/opi3.tif]

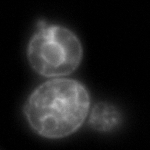

Supplement: Supplementary file 7 — Source Data for Figure 2 [file EMBJ-40-e107958-s007.zip › 2A/psd1.tif]

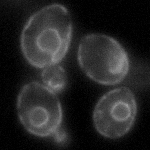

Supplement: Supplementary file 7 — Source Data for Figure 2 [file EMBJ-40-e107958-s007.zip › 2A/ura7.tif]

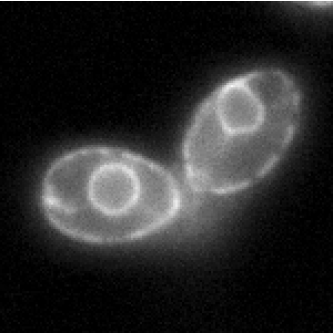

Supplement: Supplementary file 7 — Source Data for Figure 2 [file EMBJ-40-e107958-s007.zip › 2A/WT.tif]

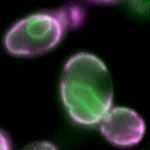

Supplement: Supplementary file 7 — Source Data for Figure 2 [file EMBJ-40-e107958-s007.zip › 2C/lnp1 merge.tif]

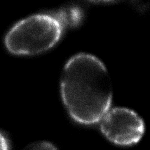

Supplement: Supplementary file 7 — Source Data for Figure 2 [file EMBJ-40-e107958-s007.zip › 2C/lnp1 Rtn1.tif]

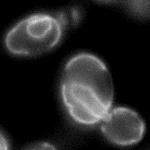

Supplement: Supplementary file 7 — Source Data for Figure 2 [file EMBJ-40-e107958-s007.zip › 2C/lnp1 Sec63.tif]

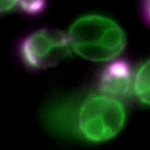

Supplement: Supplementary file 7 — Source Data for Figure 2 [file EMBJ-40-e107958-s007.zip › 2C/sey1 merge.tif]

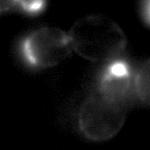

Supplement: Supplementary file 7 — Source Data for Figure 2 [file EMBJ-40-e107958-s007.zip › 2C/sey1 Rtn1.tif]

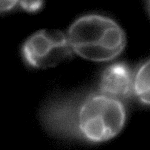

Supplement: Supplementary file 7 — Source Data for Figure 2 [file EMBJ-40-e107958-s007.zip › 2C/sey1 Sec63.tif]

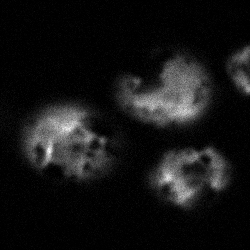

Supplement: Supplementary file 8 — Source Data for Figure 3 [file EMBJ-40-e107958-s014.zip › SourceData_Fig3/3A/WT estradiol.tif]

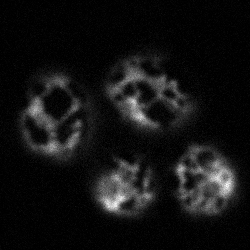

Supplement: Supplementary file 8 — Source Data for Figure 3 [file EMBJ-40-e107958-s014.zip › SourceData_Fig3/3A/WT untreated.tif]

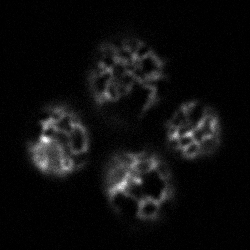

Supplement: Supplementary file 8 — Source Data for Figure 3 [file EMBJ-40-e107958-s014.zip › SourceData_Fig3/3A/ice2 untreated.tif]

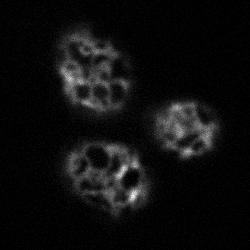

Supplement: Supplementary file 8 — Source Data for Figure 3 [file EMBJ-40-e107958-s014.zip › SourceData_Fig3/3A/ice2 estradiol.tif]

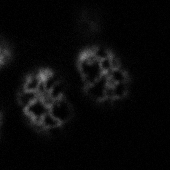

Supplement: Supplementary file 9 — Source Data for Figure 4 [file EMBJ-40-e107958-s011.zip › SourceData_Fig4/4C-D/ice2 untreated Rtn1cherry.tif]

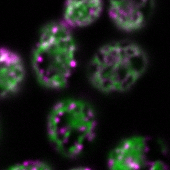

Supplement: Supplementary file 9 — Source Data for Figure 4 [file EMBJ-40-e107958-s011.zip › SourceData_Fig4/4C-D/ice2 DTT merge.tif]

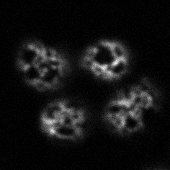

Supplement: Supplementary file 9 — Source Data for Figure 4 [file EMBJ-40-e107958-s011.zip › SourceData_Fig4/4C-D/WT untreated Rtn1cherry.tif]

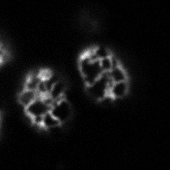

Supplement: Supplementary file 9 — Source Data for Figure 4 [file EMBJ-40-e107958-s011.zip › SourceData_Fig4/4C-D/ice2 untreated Sec63neon.tif]

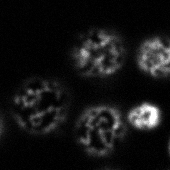

Supplement: Supplementary file 9 — Source Data for Figure 4 [file EMBJ-40-e107958-s011.zip › SourceData_Fig4/4C-D/WT DTT Rtn1cherry.tif]

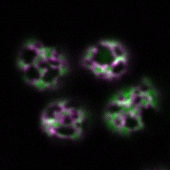

Supplement: Supplementary file 9 — Source Data for Figure 4 [file EMBJ-40-e107958-s011.zip › SourceData_Fig4/4C-D/WT untreated merge.tif]

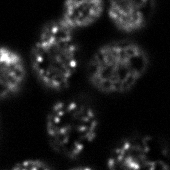

Supplement: Supplementary file 9 — Source Data for Figure 4 [file EMBJ-40-e107958-s011.zip › SourceData_Fig4/4C-D/ice2 DTT Rtn1cherry.tif]

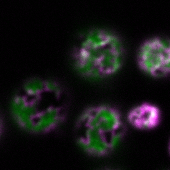

Supplement: Supplementary file 9 — Source Data for Figure 4 [file EMBJ-40-e107958-s011.zip › SourceData_Fig4/4C-D/WT DTT merge.tif]

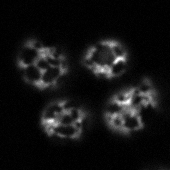

Supplement: Supplementary file 9 — Source Data for Figure 4 [file EMBJ-40-e107958-s011.zip › SourceData_Fig4/4C-D/WT untreated Sec63neon.tif]

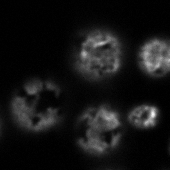

Supplement: Supplementary file 9 — Source Data for Figure 4 [file EMBJ-40-e107958-s011.zip › SourceData_Fig4/4C-D/WT DTT Sec63neon.tif]

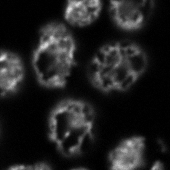

Supplement: Supplementary file 9 — Source Data for Figure 4 [file EMBJ-40-e107958-s011.zip › SourceData_Fig4/4C-D/ice2 DTT Sec63neon.tif]

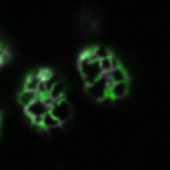

Supplement: Supplementary file 9 — Source Data for Figure 4 [file EMBJ-40-e107958-s011.zip › SourceData_Fig4/4C-D/ice2 untreated merge.tif]

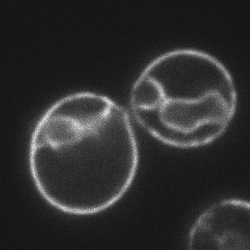

Supplement: Supplementary file 10 — Source Data for Figure 5 [file EMBJ-40-e107958-s004.zip › 5E/nem1 ice2.tif]

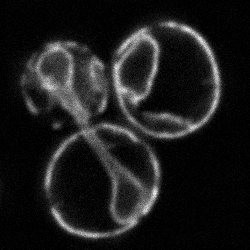

Supplement: Supplementary file 10 — Source Data for Figure 5 [file EMBJ-40-e107958-s004.zip › 5E/nem1.tif]

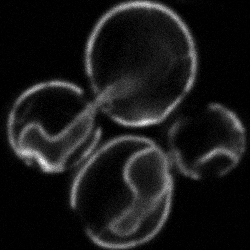

Supplement: Supplementary file 10 — Source Data for Figure 5 [file EMBJ-40-e107958-s004.zip › 5E/spo7 ice2.tif]

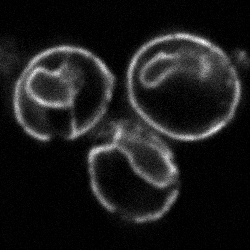

Supplement: Supplementary file 10 — Source Data for Figure 5 [file EMBJ-40-e107958-s004.zip › 5E/spo7.tif]

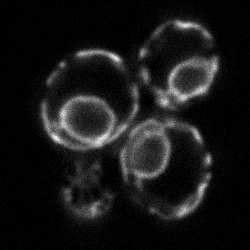

Supplement: Supplementary file 10 — Source Data for Figure 5 [file EMBJ-40-e107958-s004.zip › 5E/WT.tif]

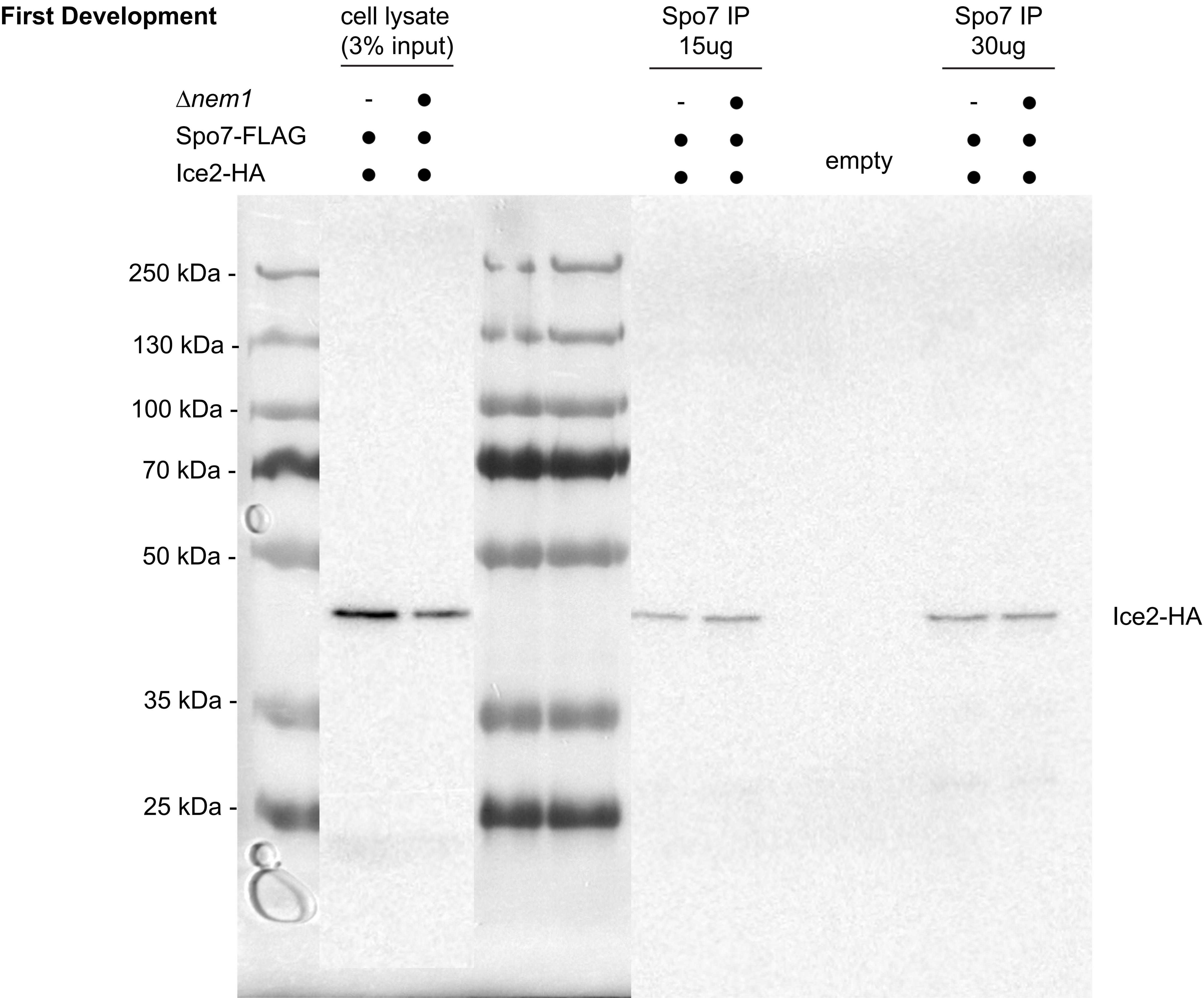

Supplement: Supplementary file 12 — Source Data for Figure 7 [file EMBJ-40-e107958-s006.zip › 7C.pdf]

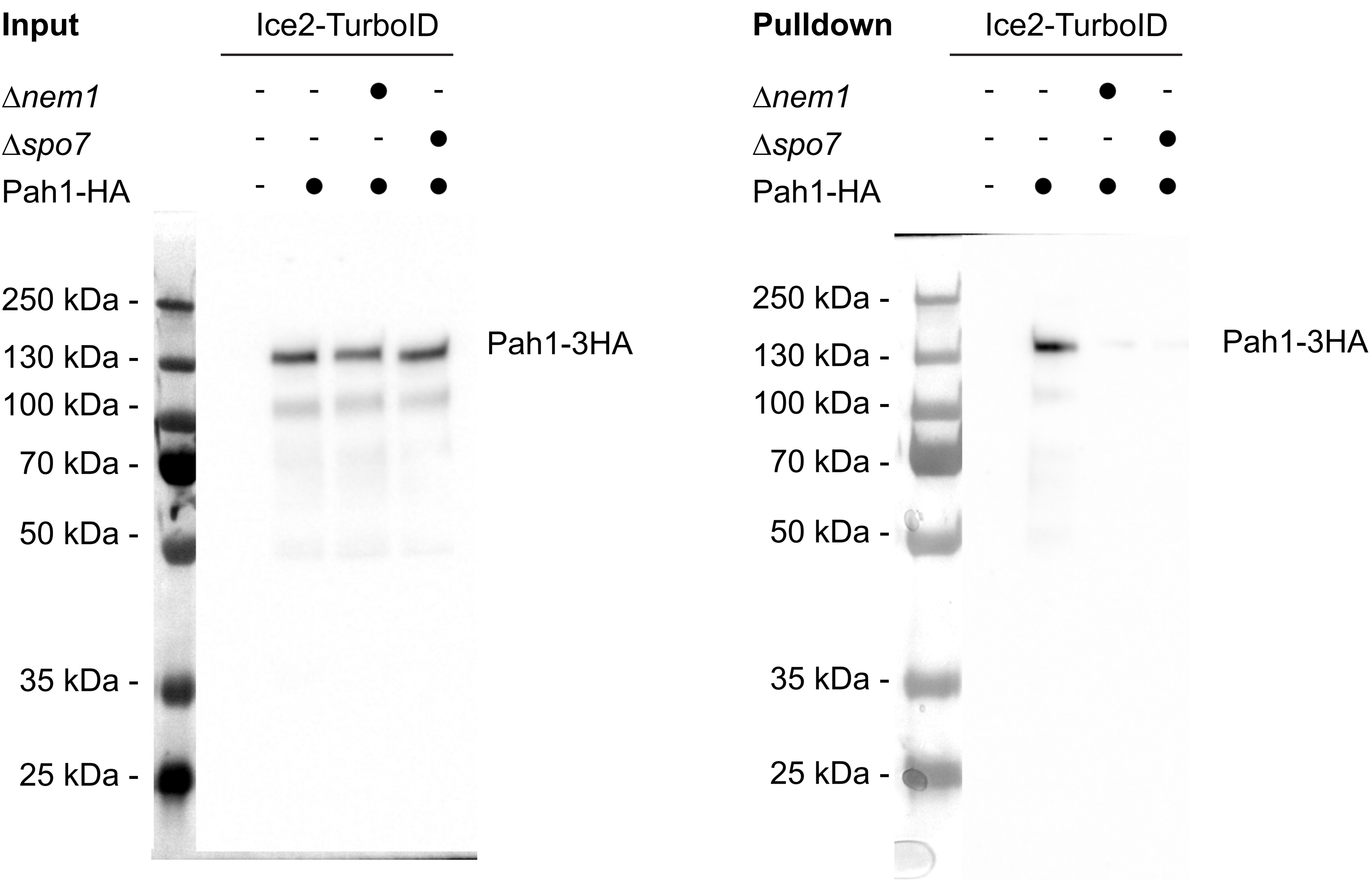

Note: input and pulldown samples were run on separate gels.

Supplement: Supplementary file 12 — Source Data for Figure 7 [file EMBJ-40-e107958-s006.zip › 7D.pdf]

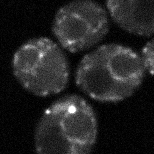

Supplement: Supplementary file 12 — Source Data for Figure 7 [file EMBJ-40-e107958-s006.zip › 7E/Ice2scarlet Nem1neon green channel.tif]

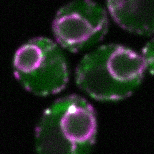

Supplement: Supplementary file 12 — Source Data for Figure 7 [file EMBJ-40-e107958-s006.zip › 7E/Ice2scarlet Nem1neon merge.tif]

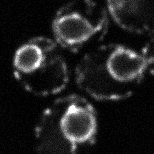

Supplement: Supplementary file 12 — Source Data for Figure 7 [file EMBJ-40-e107958-s006.zip › 7E/Ice2scarlet Nem1neon red channel.tif]

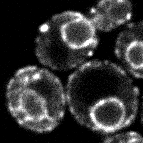

Supplement: Supplementary file 12 — Source Data for Figure 7 [file EMBJ-40-e107958-s006.zip › 7E/Ice2scarlet Spo7neon green channel.tif]

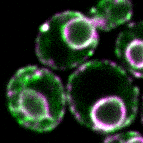

Supplement: Supplementary file 12 — Source Data for Figure 7 [file EMBJ-40-e107958-s006.zip › 7E/Ice2scarlet Spo7neon merge.tif]

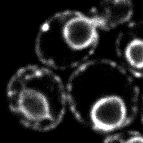

Supplement: Supplementary file 12 — Source Data for Figure 7 [file EMBJ-40-e107958-s006.zip › 7E/Ice2scarlet Spo7neon red channel.tif]

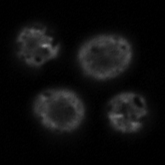

Supplement: Supplementary file 14 — Source Data for Figure 9 [file EMBJ-40-e107958-s005.zip › SourceData_Fig9/9A/opi1 pICE2 cortical.tif]

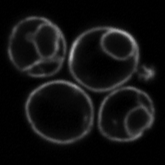

Supplement: Supplementary file 14 — Source Data for Figure 9 [file EMBJ-40-e107958-s005.zip › SourceData_Fig9/9A/opi1 pICE2 mid.tif]

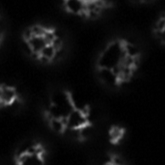

Supplement: Supplementary file 14 — Source Data for Figure 9 [file EMBJ-40-e107958-s005.zip › SourceData_Fig9/9A/WT cortical.tif]

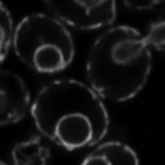

Supplement: Supplementary file 14 — Source Data for Figure 9 [file EMBJ-40-e107958-s005.zip › SourceData_Fig9/9A/WT mid.tif]

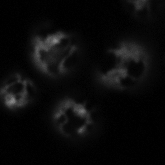

Supplement: Supplementary file 14 — Source Data for Figure 9 [file EMBJ-40-e107958-s005.zip › SourceData_Fig9/9A/pICE2 cortical.tif]

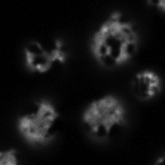

Supplement: Supplementary file 14 — Source Data for Figure 9 [file EMBJ-40-e107958-s005.zip › SourceData_Fig9/9A/opi1 cortical.tif]

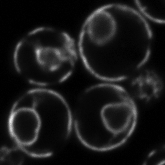

Supplement: Supplementary file 14 — Source Data for Figure 9 [file EMBJ-40-e107958-s005.zip › SourceData_Fig9/9A/opi1 mid.tif]

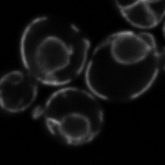

Supplement: Supplementary file 14 — Source Data for Figure 9 [file EMBJ-40-e107958-s005.zip › SourceData_Fig9/9A/pICE2 mid.tif]
