# Supplementary material for: Ice2 promotes ER membrane biogenesis in yeast by inhibiting the conserved lipin phosphatase complex
Source: EMBO J. 2021 Oct 6;40(22):e107958. doi: 10.15252/embj.2021107958 (PMC8591542; doi:10.15252/embj.2021107958)
Supplement: Supplementary file 6 — Source Data for Figure 1 [file EMBJ-40-e107958-s010.zip › 1D.pdf]

First development

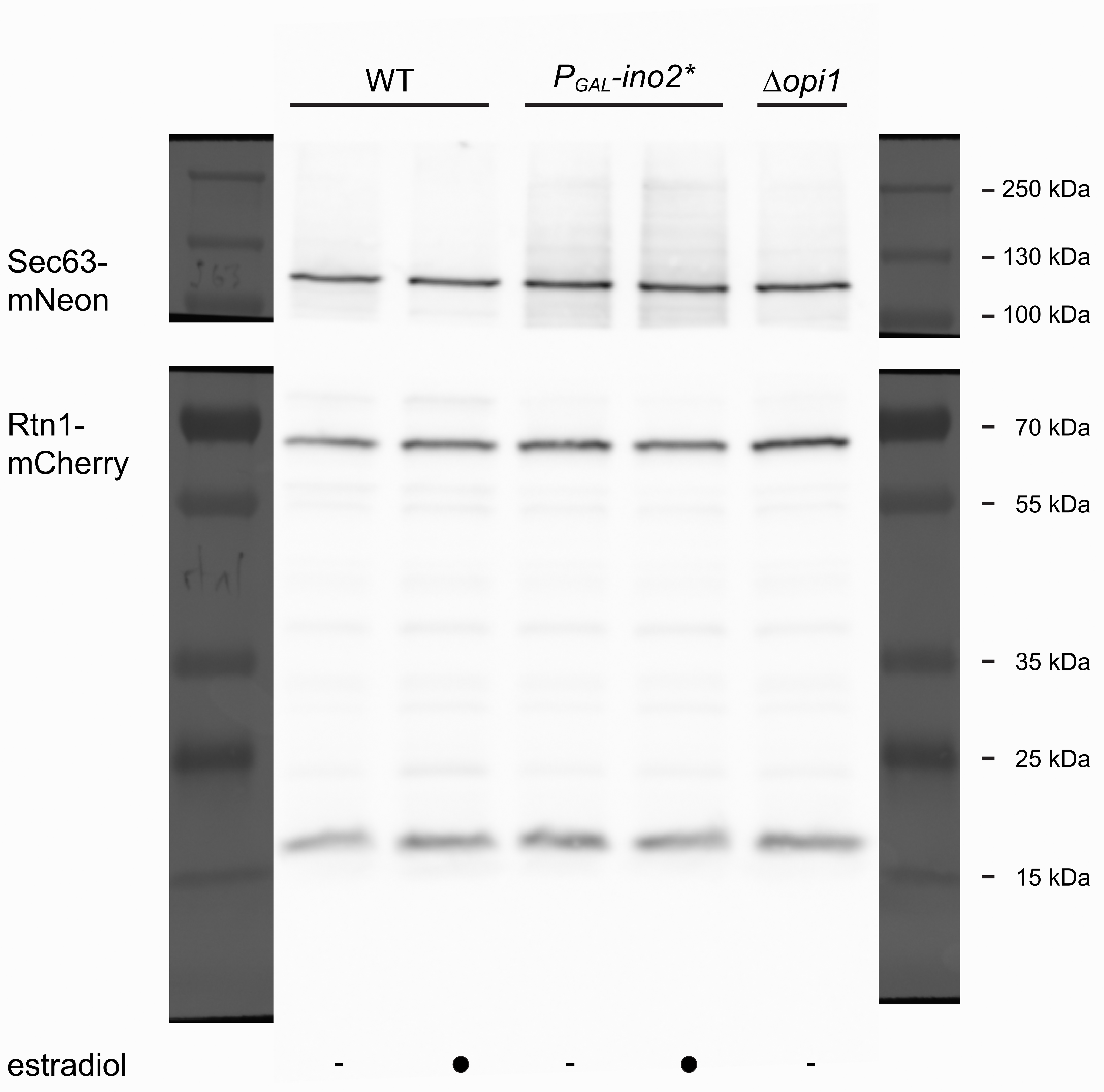

Note: the membrane was cut between the 70 kDa and 100 kDa marker bands and the upper part was developed with the anti-Sec63 and the lower part with the anti-mCherry antibody.

Second development

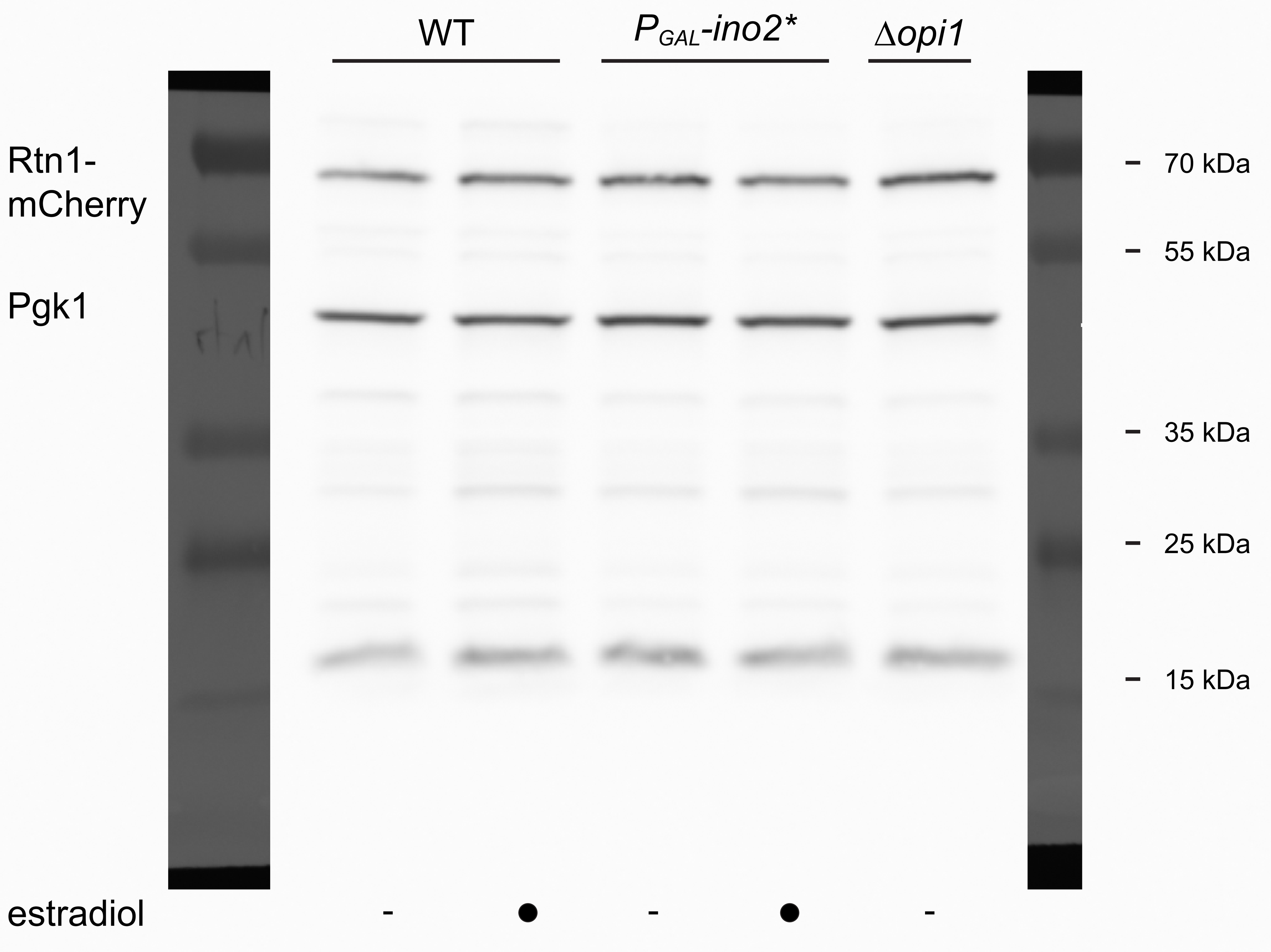

Note: the lower part of the membrane was re-developed with the anti-Pgk1 antibody.
