# Supplementary material for: Ice2 promotes ER membrane biogenesis in yeast by inhibiting the conserved lipin phosphatase complex
Source: EMBO J. 2021 Oct 6;40(22):e107958. doi: 10.15252/embj.2021107958 (PMC8591542; doi:10.15252/embj.2021107958)
Supplement: Supplementary file 11 — Source Data for Figure 6 [file EMBJ-40-e107958-s013.zip › 6B.pdf]

SDS-PAGE gel

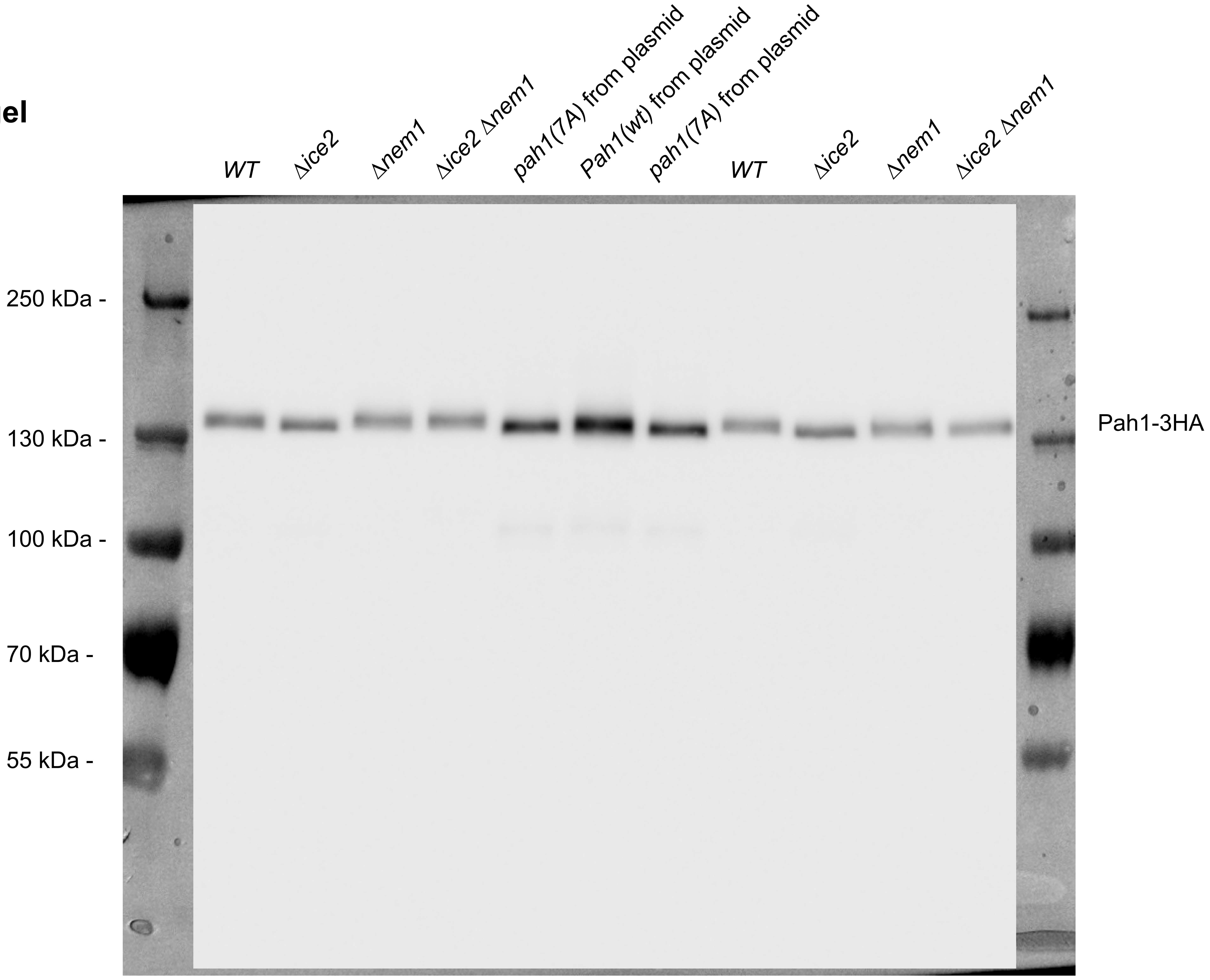

Note: the first four lanes used for Figure 6B.

Phos-tag PAGE gel

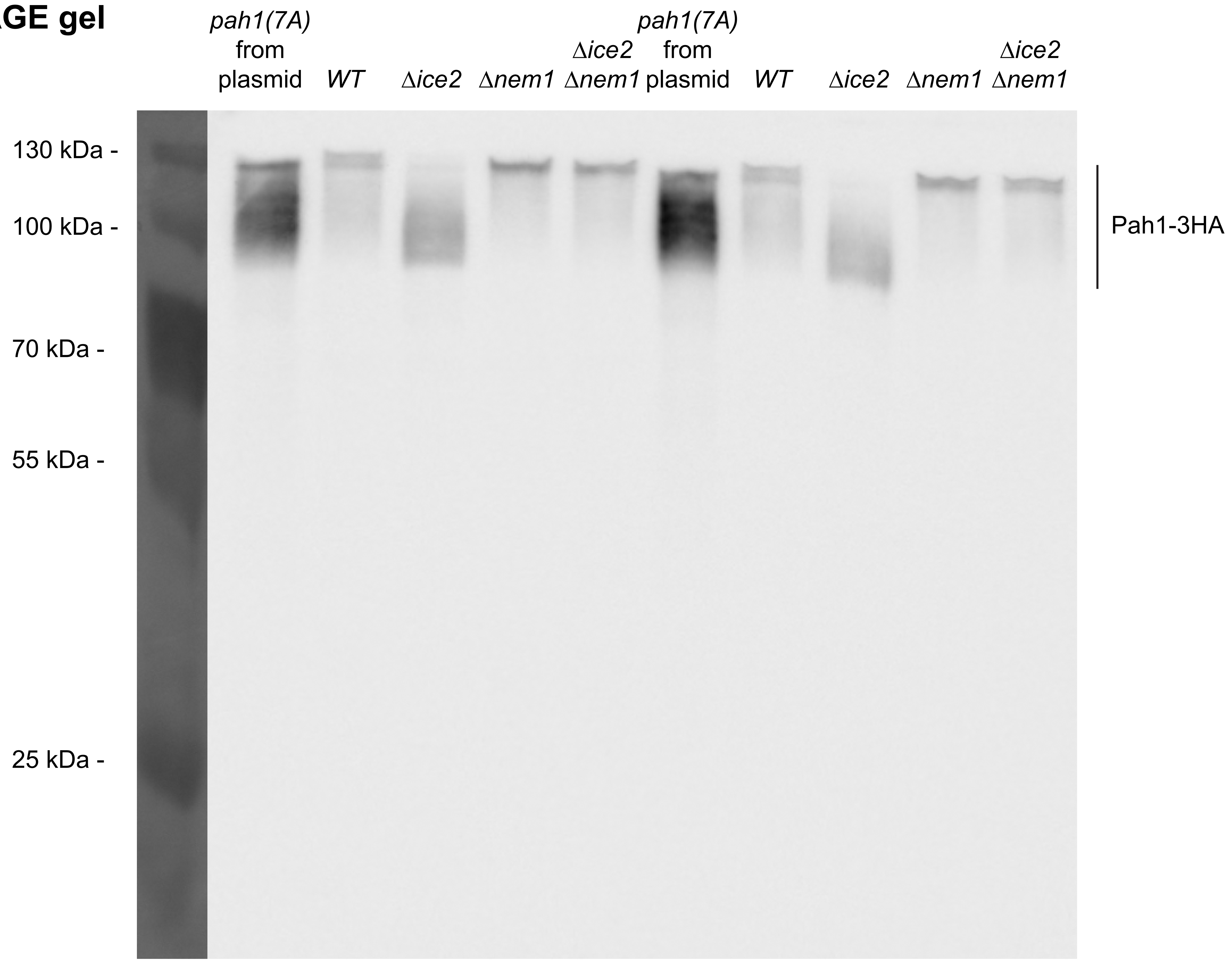

Note: lanes 2-5 were used for Figure 6B. The molecular weight marker runs abnormally on Phos-tag gels.
