# Supplementary material for: Ice2 promotes ER membrane biogenesis in yeast by inhibiting the conserved lipin phosphatase complex
Source: EMBO J. 2021 Oct 6;40(22):e107958. doi: 10.15252/embj.2021107958 (PMC8591542; doi:10.15252/embj.2021107958)
Supplement: Supplementary file 11 — Source Data for Figure 6 [file EMBJ-40-e107958-s013.zip › 6D_F.pdf]

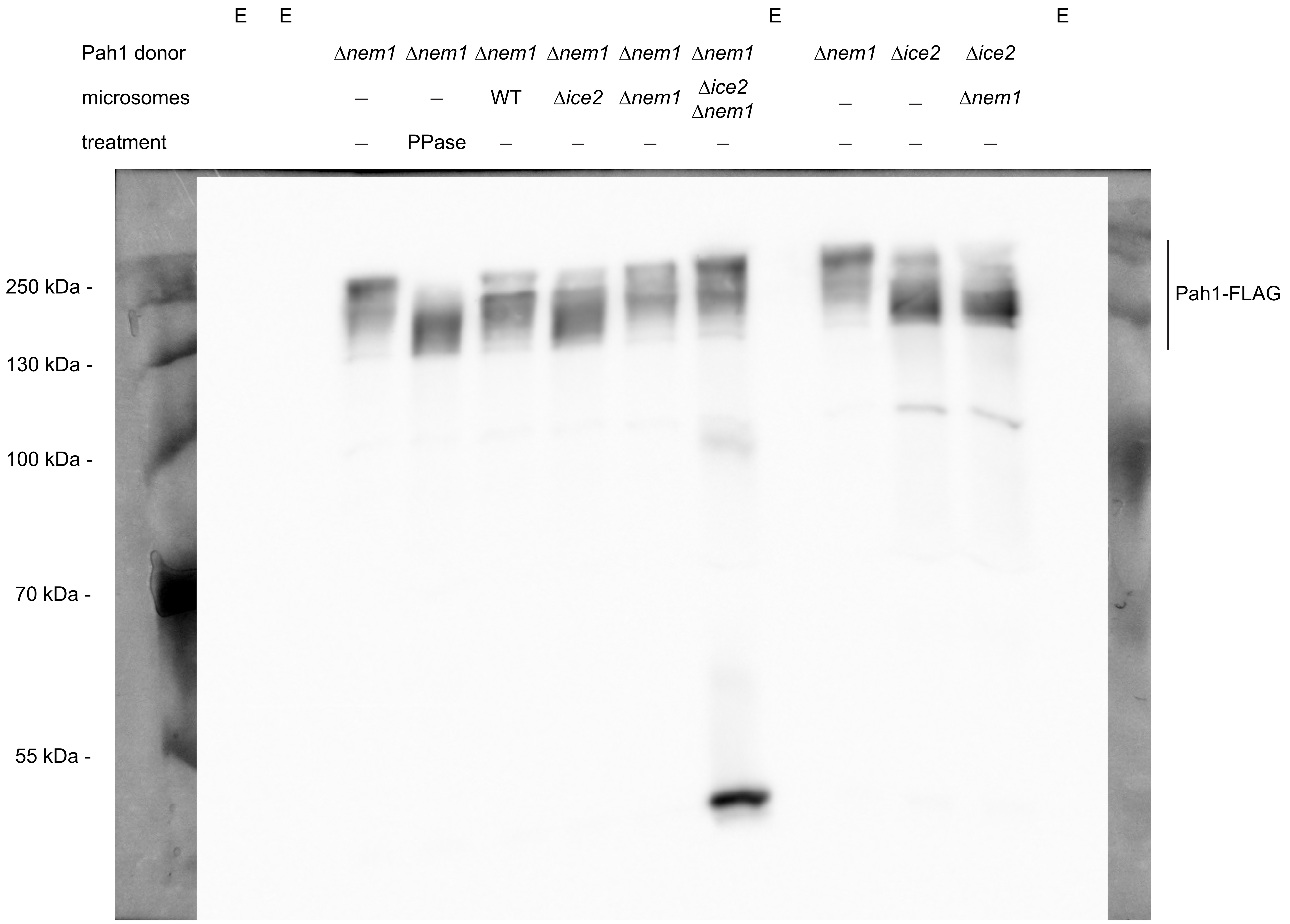

Note: lanes 3-8 were used for Figure 6D, lanes 10-12 were used for Figure 6F. The identity of the fast-migrating band in lane 8 is unclear. The molecular weight marker runs abnormally on Phos-tag gels. E: empty lane.
