# Supplementary material for: Ice2 promotes ER membrane biogenesis in yeast by inhibiting the conserved lipin phosphatase complex
Source: EMBO J. 2021 Oct 6;40(22):e107958. doi: 10.15252/embj.2021107958 (PMC8591542; doi:10.15252/embj.2021107958)

First development

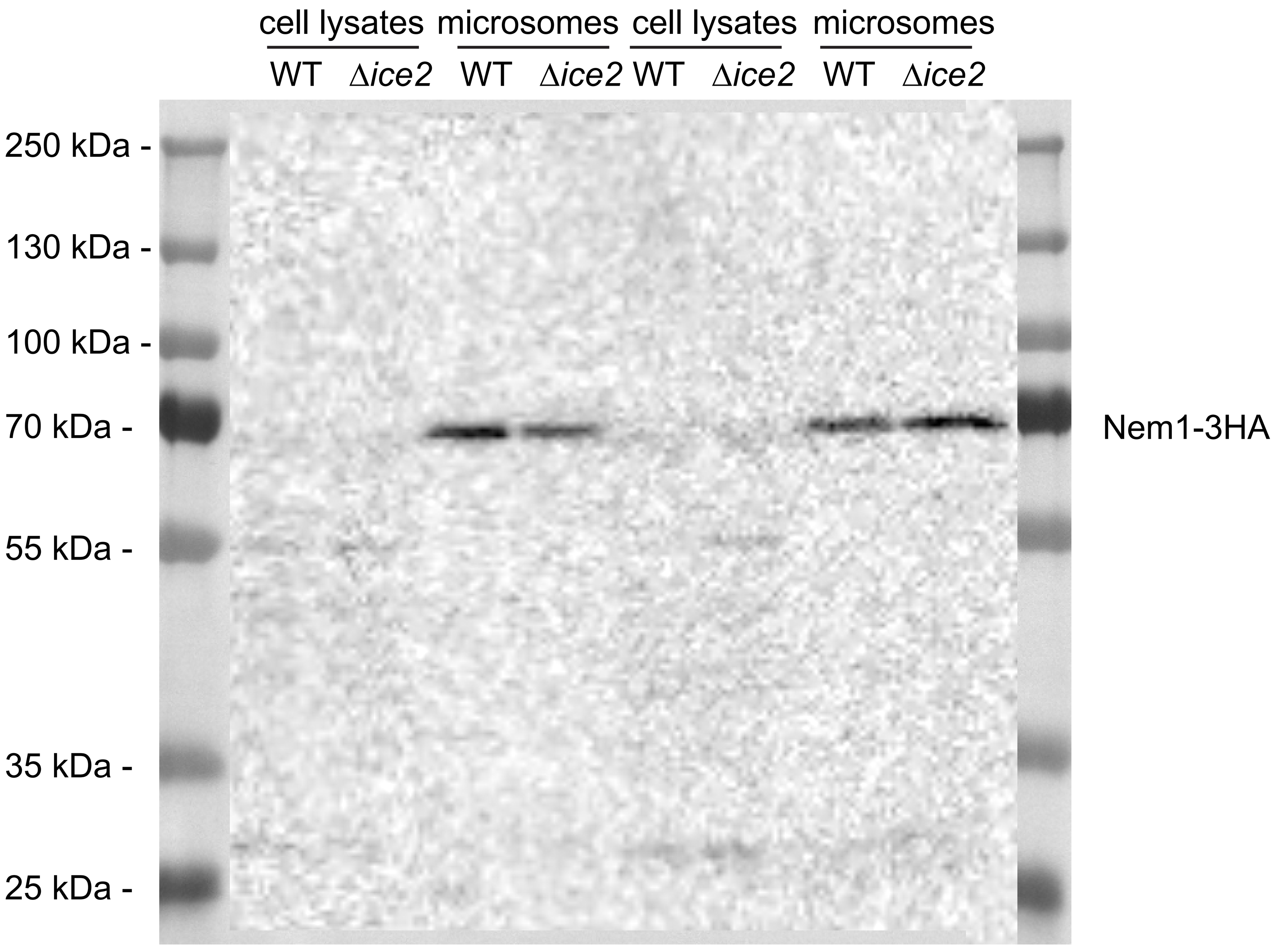

Note: the membrane was fist developed with the anti-HA antibody, then with the anti-Sec61 antibody and finally with the anti-Pgk1 antibody. Lanes 5-8 were used for Figure 6E.

Second development

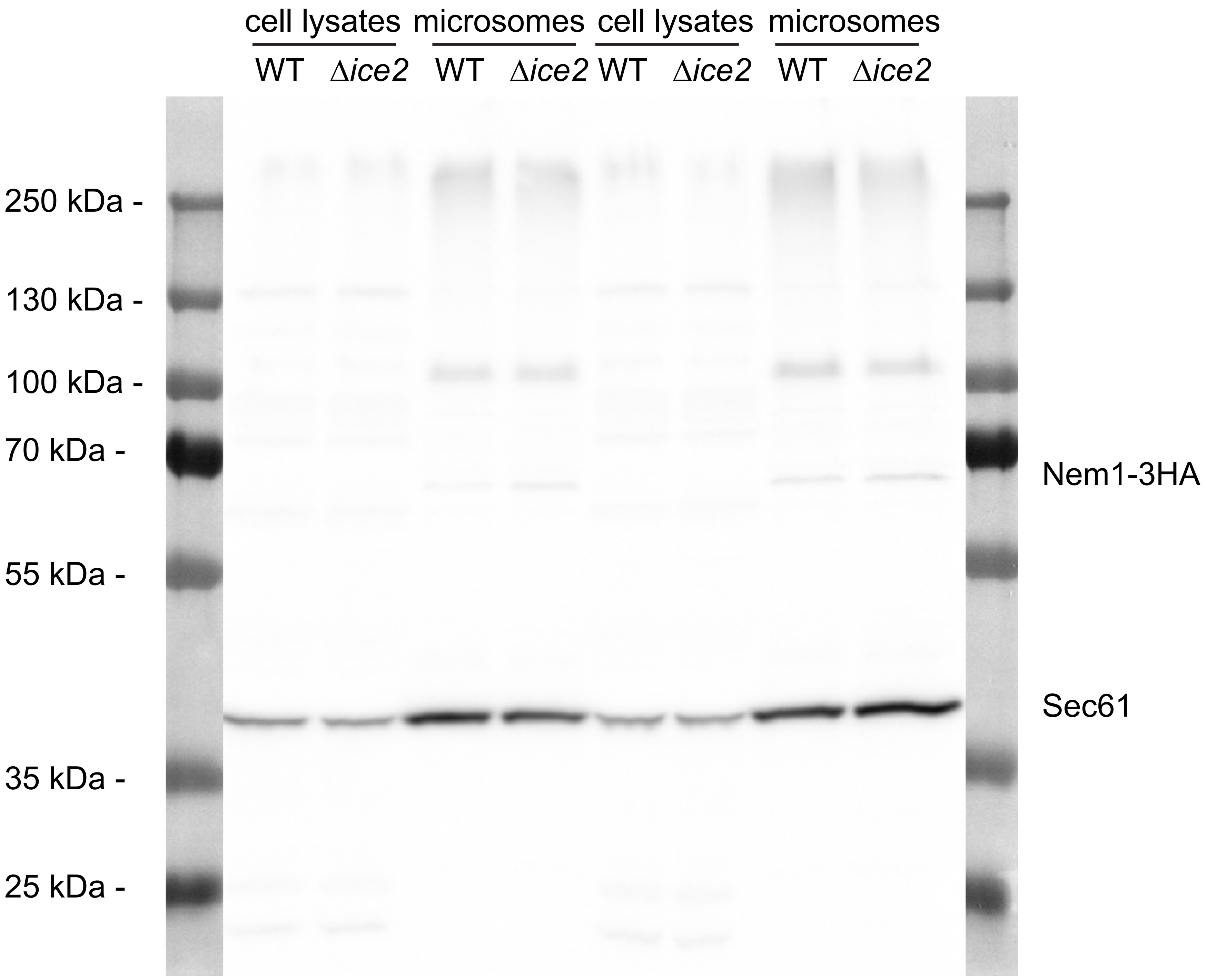

Third development

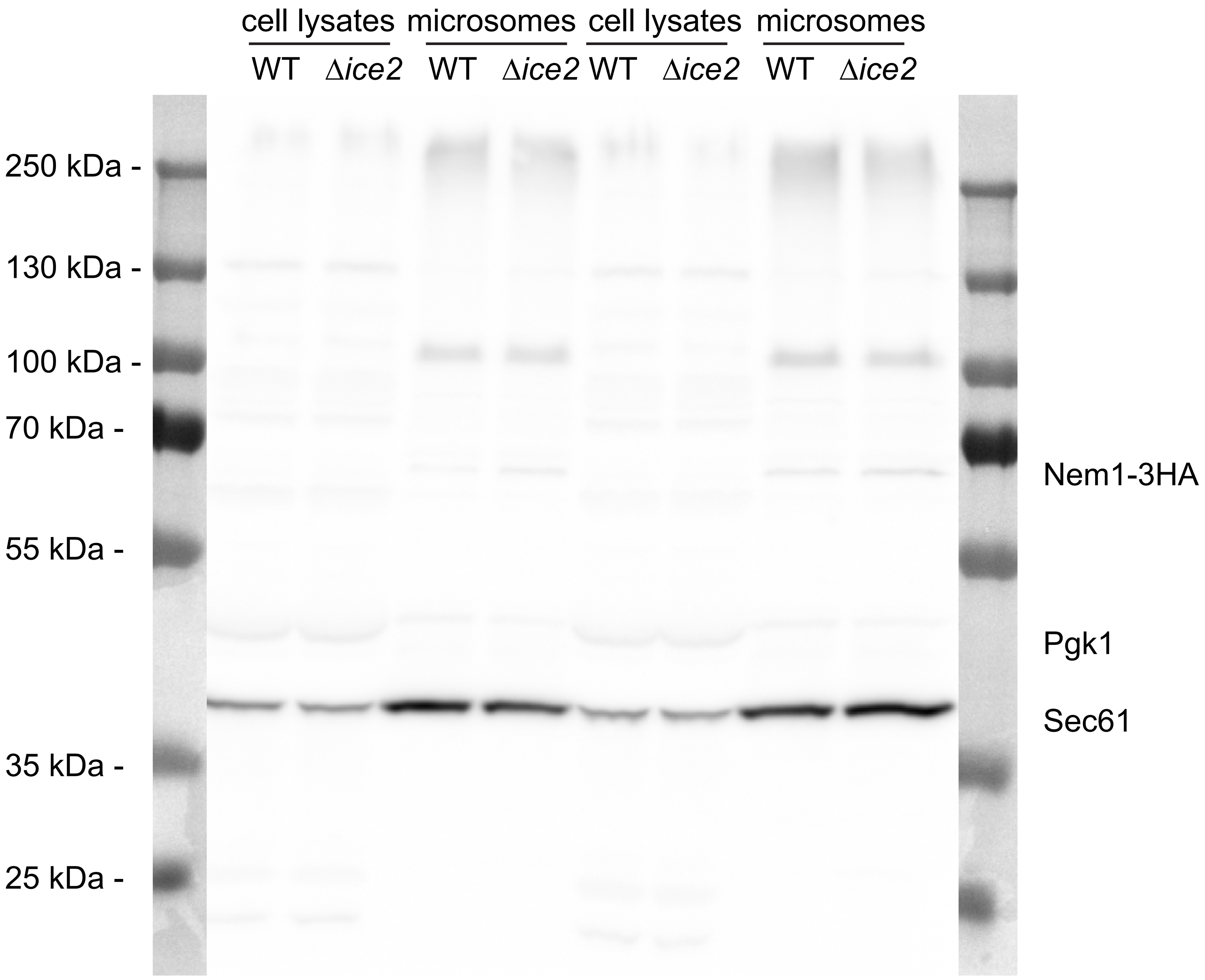

Supplement: Supplementary file 11 — Source Data for Figure 6 [file EMBJ-40-e107958-s013.zip › 6E.pdf]
