# Supplementary material for: Ice2 promotes ER membrane biogenesis in yeast by inhibiting the conserved lipin phosphatase complex
Source: EMBO J. 2021 Oct 6;40(22):e107958. doi: 10.15252/embj.2021107958 (PMC8591542; doi:10.15252/embj.2021107958)

First development

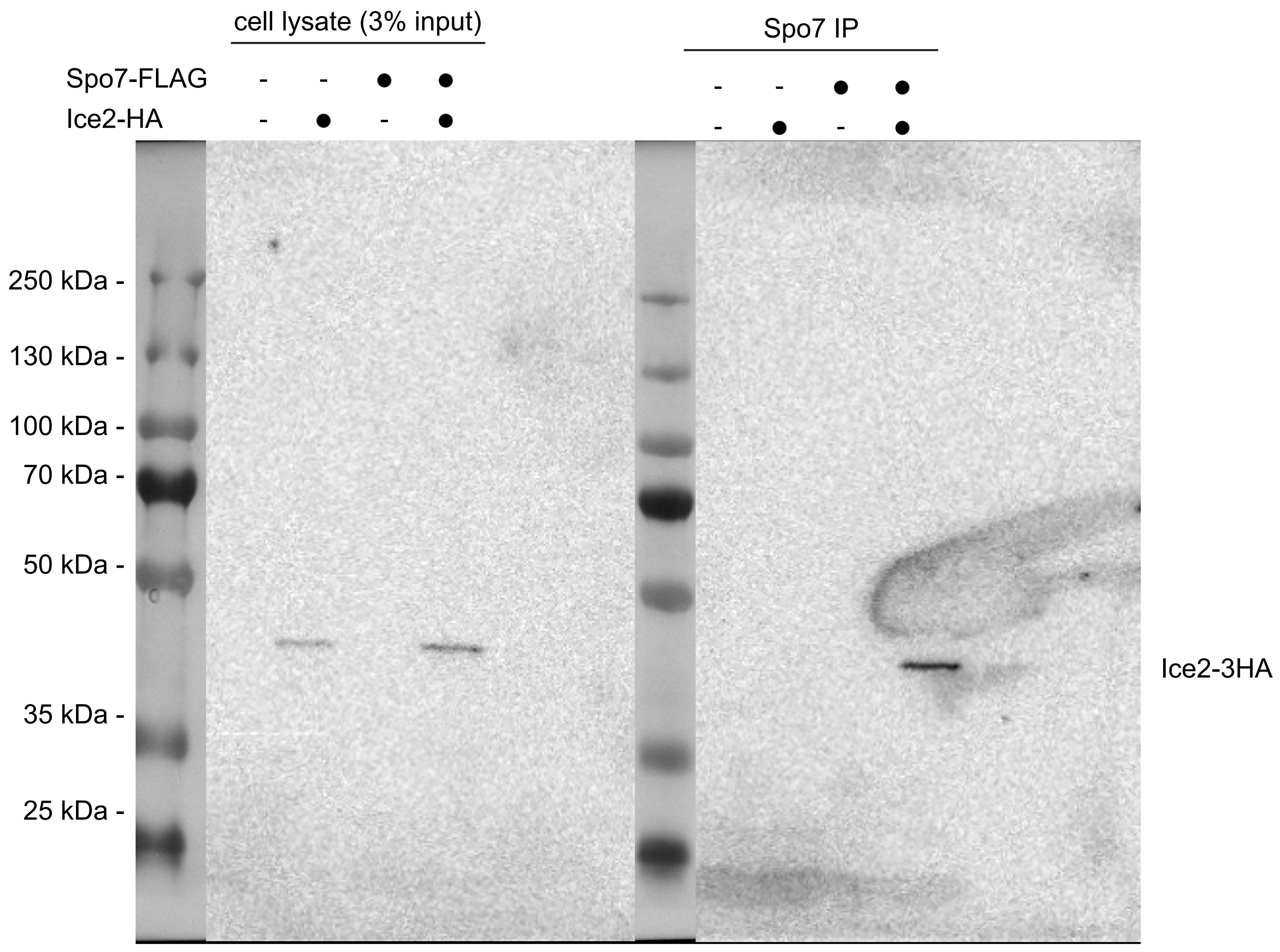

Note: The membrane was first developed with the anti-HA antibody, then with the anti-FLAG antibody and finally with the anti-Dpm1 antibody. The membrane was cut before the third development.

Second development (exposure chosen so that Spo7-FLAG visible)

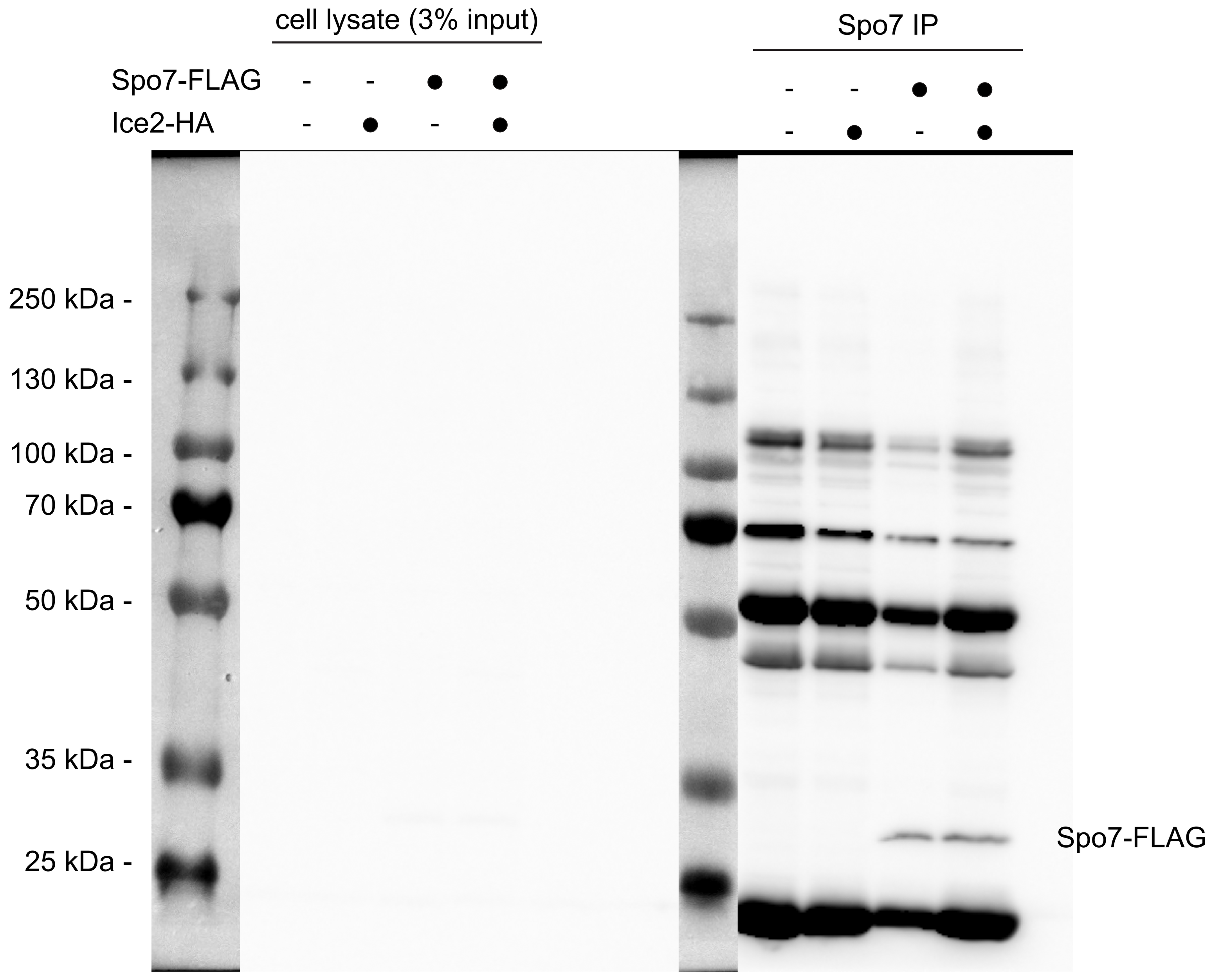

Second development (levels adjusted so that Spo7-FLAG in cell lysate visible)

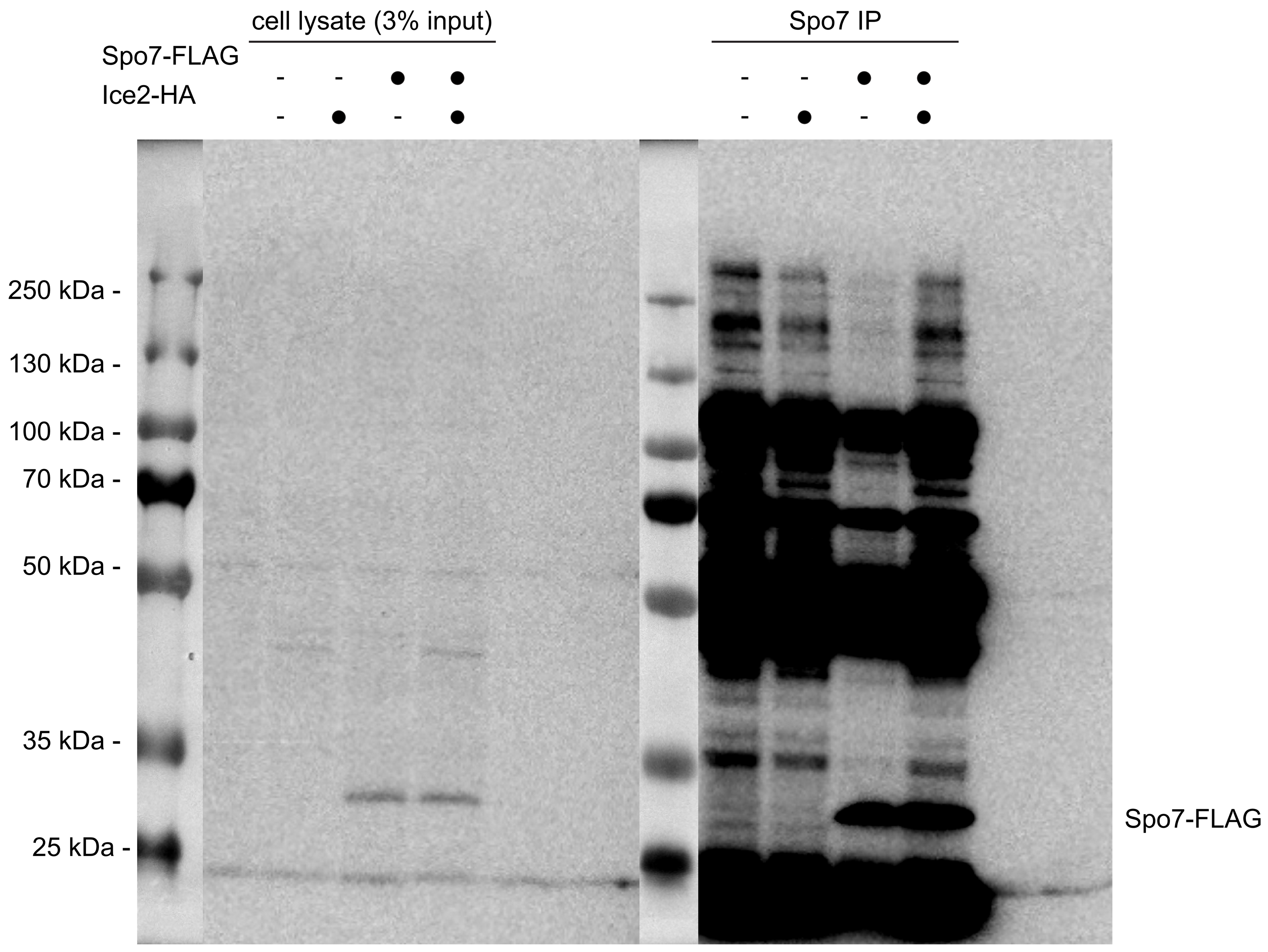

Third development (exposure chosen so that Dpm1 in cell lysate visible)

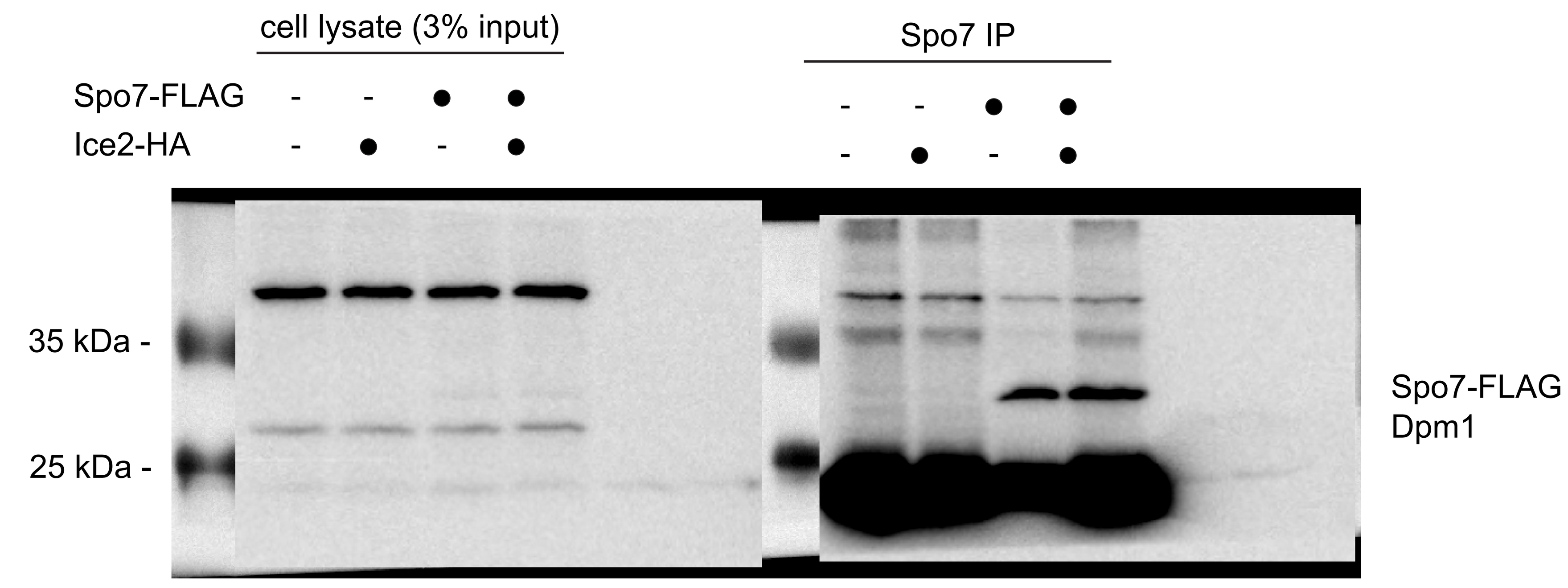

Supplement: Supplementary file 12 — Source Data for Figure 7 [file EMBJ-40-e107958-s006.zip › 7A.pdf]
