# Supplementary material for: Ice2 promotes ER membrane biogenesis in yeast by inhibiting the conserved lipin phosphatase complex
Source: EMBO J. 2021 Oct 6;40(22):e107958. doi: 10.15252/embj.2021107958 (PMC8591542; doi:10.15252/embj.2021107958)
Supplement: Supplementary file 12 — Source Data for Figure 7 [file EMBJ-40-e107958-s006.zip › 7B.pdf]

First development

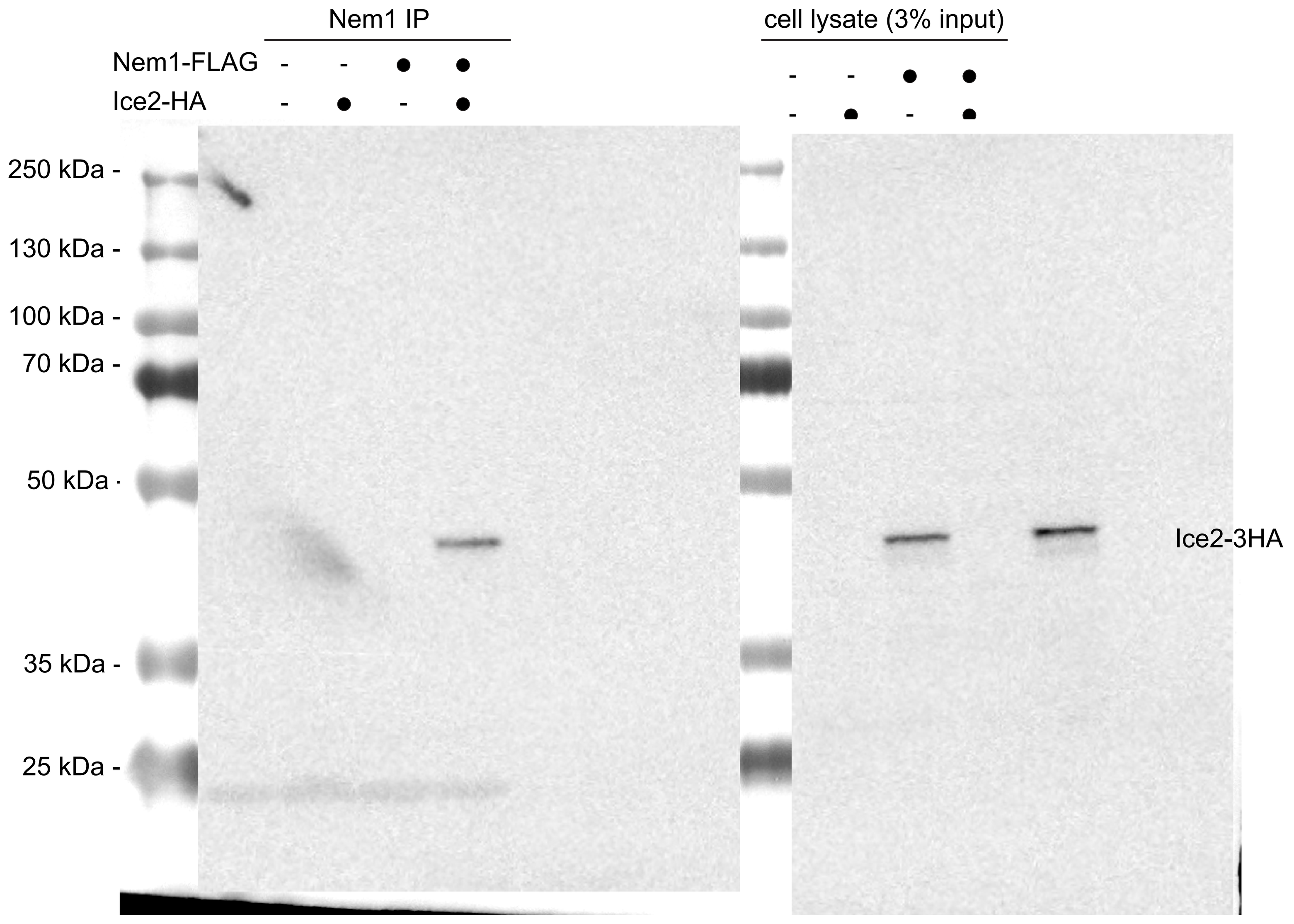

Second development (not used)

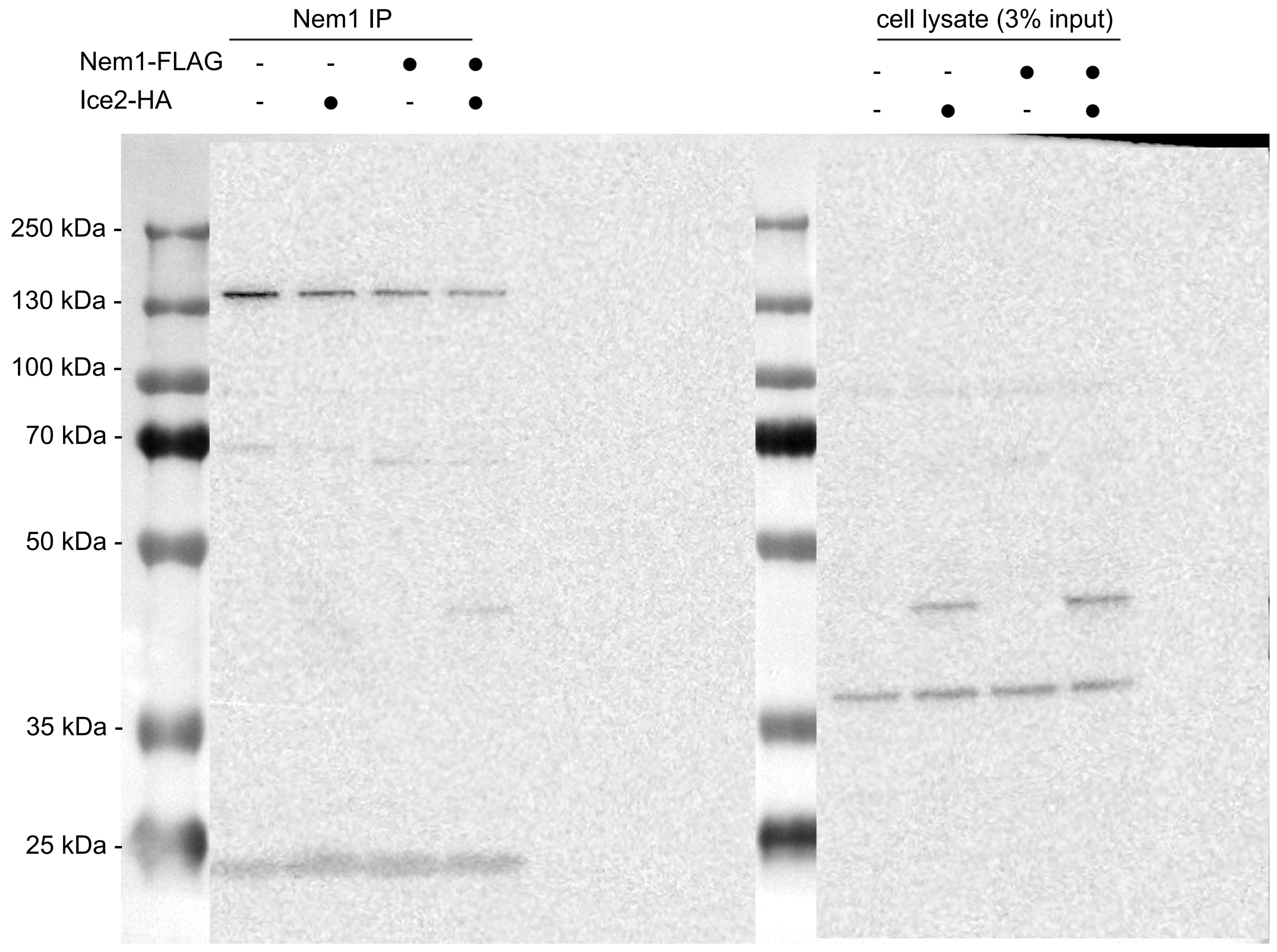

Third development

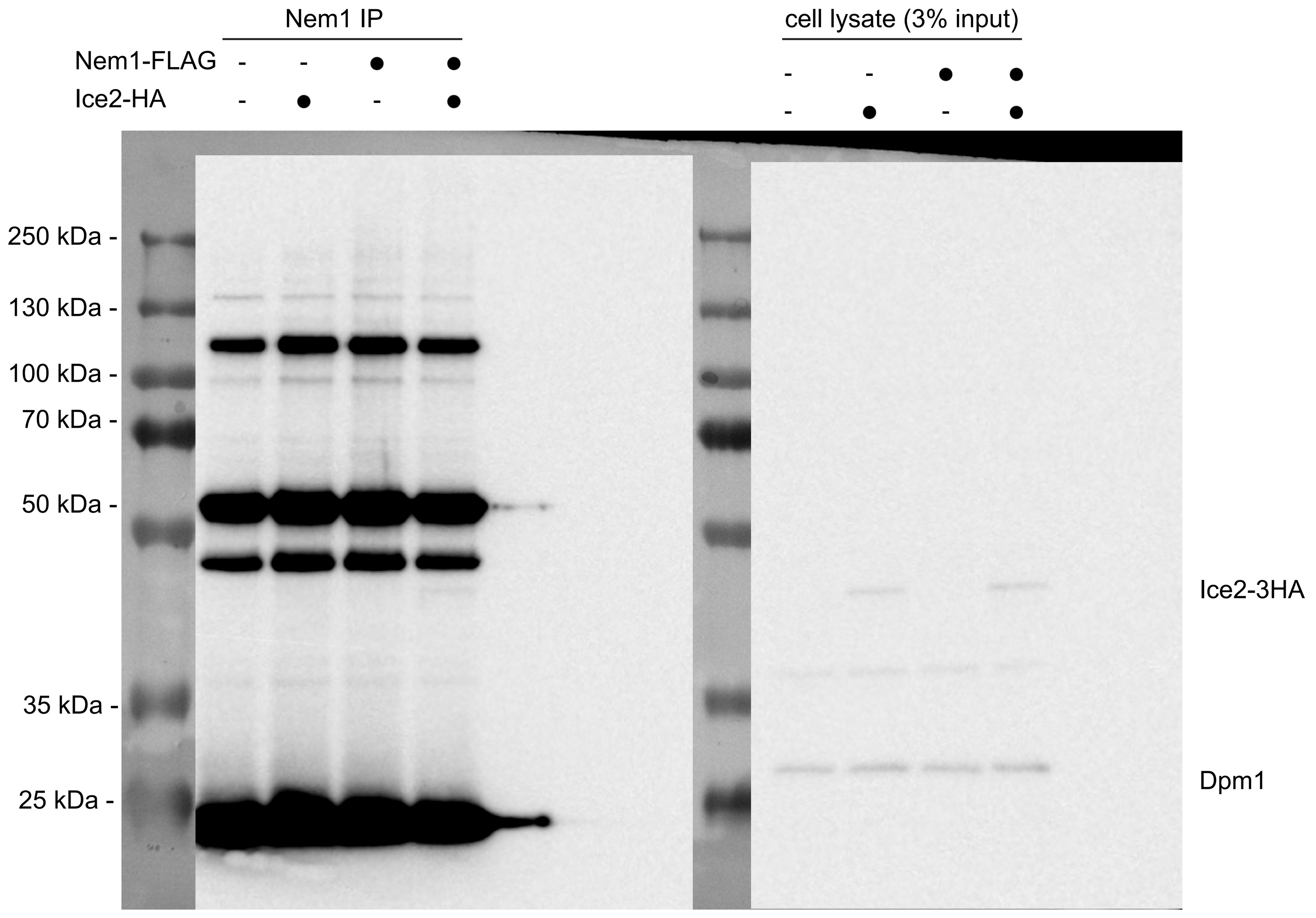

Fourth development

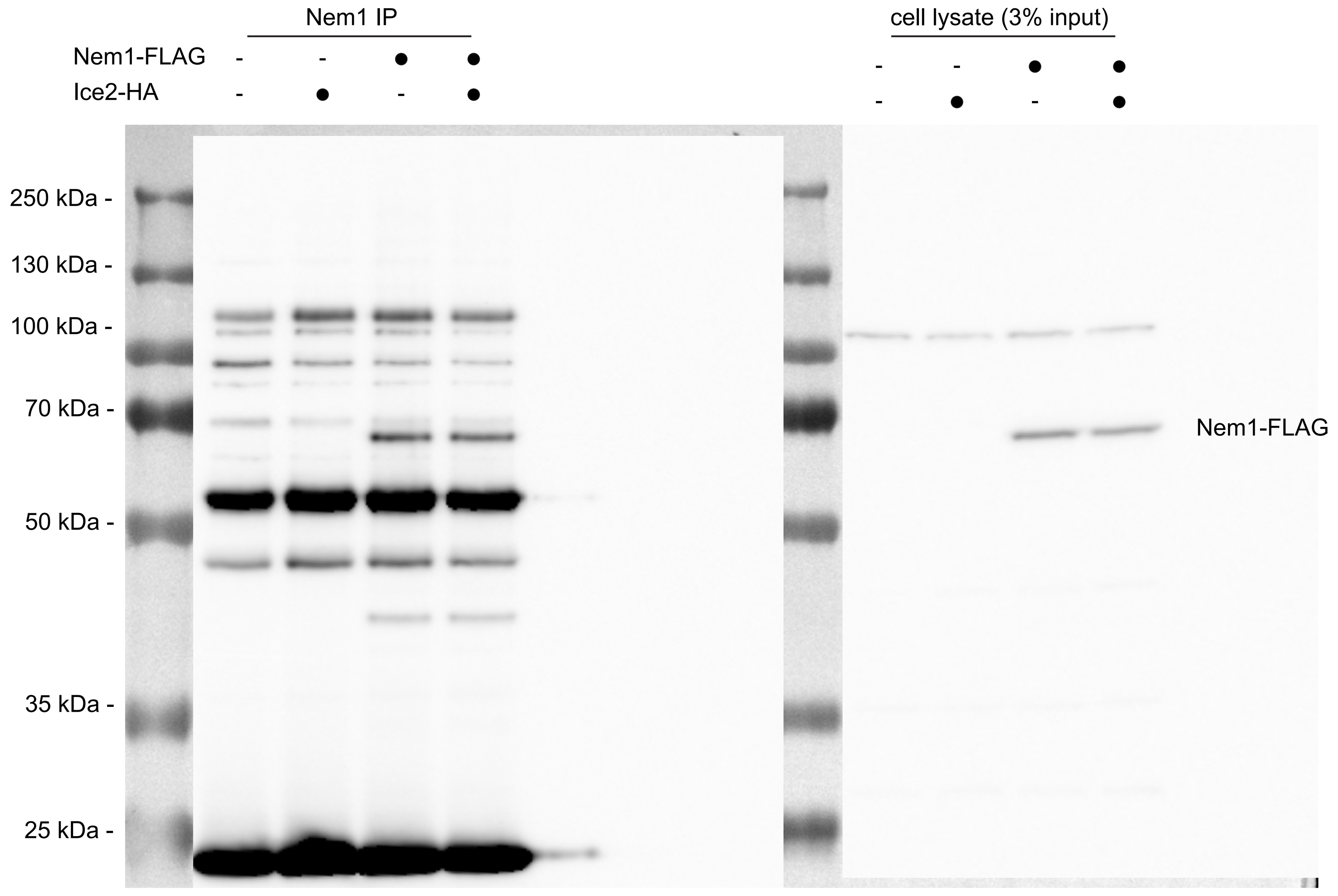

Note: The membrane was first developed with the anti-HA antibody and then with the anti-FLAG antibody F7425 (Sigma). The second development was not used for Figure 7B.
Then followed development with the anti-Dpm1 antibody and finally with the anti-FLAG antibody M2 (Sigma).
