# Supplementary material for: Ice2 promotes ER membrane biogenesis in yeast by inhibiting the conserved lipin phosphatase complex
Source: EMBO J. 2021 Oct 6;40(22):e107958. doi: 10.15252/embj.2021107958 (PMC8591542; doi:10.15252/embj.2021107958)
Supplement: Supplementary file 15 [file EMBJ-40-e107958-s008.pdf]

THE EMBO JOURNAL

Ice2 promotes ER membrane biogenesis in yeast by inhibiting the conserved lipin phosphatase complex

Dimitrios Papagiannidis, Peter Bircham, Christian Lüchtenborg, Oliver Pajonk, Giulia Ruffini, Britta Brügger, and Sebastian Schuck
DOI: 10.15252/embj.2021107958

Corresponding author: Sebastian Schuck (s.schuck@zmbh.uni-heidelberg.de)

Review Timeline:

Submission Date:	7th Feb 21
Editorial Decision:	10th Feb 21
Appeal Received:	15th Feb 21
Editor Correspondence:	22nd Feb 21
Editorial Decision:	18th Mar 21
Revision Received:	15th Jul 21
Editorial Decision:	23rd Aug 21
Revision Received:	1st Sep 21
Accepted:	3rd Sep 21

Editors: Elisabetta Argenzio / Stefanie Boehm

Transaction Report:

(Note: With the exception of the correction of typographical or spelling errors that could be a source of ambiguity, letters and reports are not edited. Depending on transfer agreements, referee reports obtained elsewhere may or may not be included in this compilation. Referee reports are anonymous unless the Referee chooses to sign their reports.)

1st Editorial Decision 10th Feb 2021

Dear Dr. Schuck,

Thank you for submitting your manuscript (EMBOJ-2021-107958) to The EMBO Journal. I have now carefully read your study and discussed the work with the other members of the editorial team. I regret to inform you that we have decided not to pursue publication of this manuscript.

We appreciate that you identify ER transmembrane protein Ice2 as a regulator of ER membrane expansion in Saccharomyces cerevisiae. You report that Ice2 promotes ER membrane biogenesis by inhibiting the Nem1-Spo7 phosphatase complex and preventing the activation of phosphatidic acid phosphatase Pah1. Furthermore, you show that Ice2 cooperates with the transcriptional regulation of genes involved in lipid synthesis and maintains ER homeostasis during ER stress.

We recognize that you are the first to link Ice2 to Nem1-Spo7-Pah1 for ER membrane biogenesis. However, we notice that Ice2 has been previously implicated in the formation and maintenance of the peripheral ER network, as well as in lipid metabolism. Consequently, in our view, the advance provided is not sufficiently striking for publication in The EMBO Journal and we have decided not to move forward with it.

I thank you for giving The EMBO Journal the opportunity to consider this work and wish you success with the rapid publication of your manuscript at another venue.

Yours sincerely,

Elisabetta Argenzio, PhD
Editor
The EMBO Journal

Dear Dr. Argenzio,

Thank you for getting back to us so quickly. Your response made us realize that our cover letter, and perhaps our manuscript as well, should have contained a more comprehensive portrayal of previous studies of Ice2. Please let us provide this information here.

As you pointed out, Ice2 has been linked to ER morphogenesis and lipid metabolism before. A disparate collection of papers has implicated Ice2 in ER morphogenesis (1), nuclear protein targeting (2), ER inheritance (3), ER zinc homeostasis (4), phospholipid metabolism (5), lipid mobilization from lipid droplets (6), and ER-to-plasma membrane tethering (7). All of these studies deal with various aspects of the complex Ice2 knockout phenotype, but not one of them contains any information about the molecular interactions and functions of Ice2. Thus, they show that Ice2 is an important molecule, but it has remained enigmatic what Ice2 really does in molecular terms. Our work finally resolves this long-standing question. We therefore believe that our results are a substantial advance and that they would be of great interest to researchers in the field or organelle biogenesis.

Moreover, our paper goes beyond defining the molecular function of Ice2. Through Ice2, we connect ER membrane biogenesis to the regulation of Pah1/lipin and thereby extend the functional scope of lipin, which is already known as a (patho)physiologically important player in lipid metabolism. This makes our findings broadly relevant and, as we believe, an excellent fit for EMBO Journal.

We would therefore greatly appreciate if you could take the time to reconsider our manuscript.

Sincerely,
Sebastian Schuck

1. Estrada de Martin et al., J Cell Sci 2005
2. Murthi and Hopper, Genetics 2005
3. Loewen et al., J Cell Biol 2007
4. North et al., PLoS Genet 2012
5. Tavassoli et al., EMBO Rep 2013
6. Markgraf et al., Cell Rep 2014
7. Quon et al., PLoS Biol 2018

--

22nd Feb 21

Dear Dr. Schuck,

Thank you for contacting me regarding our recent decision on your manuscript and my apologies for the delay in reaching out.

I have now carefully re-assessed the manuscript and discussed the points in your rebuttal with the editorial team.

Although we feel that some of our initial concerns still holds, particularly the lack of mechanistic insight, I am happy to inform you that we have decided to send your manuscript out for peer-review.

Best regards,

Elisabetta

Elisabetta Argenzio, PhD
Editor
The EMBO Journal
--

Editorial Decision **18th Mar 2021**

Dear Dr. Schuck,

Thank you for submitting your manuscript entitled "Ice2 promotes ER membrane biogenesis in yeast by inhibiting the conserved lipin phosphatase complex" [EMBOJ-2021-107958R-Q] to The EMBO Journal. Your study has now been assessed by three reviewers, whose reports are enclosed below.

As you can see, the referees find the work potentially interesting. While referee #2 and #3 mainly raise minor points, referee #1 states that substantially more mechanistic insight into Ice2 function is necessary for publication in The EMBO Journal. We concur with referee #1 that this is an essential point for publication here.

Given the overall interest of your study, we have decided to invite you to submit a new version of the manuscript revised according to the referees' requests and, in particular, addressing point 1-3 from referee #1. I should add that it is The EMBO Journal policy to allow only a single round of revision, and acceptance of your manuscript will therefore depend on the completeness of your responses in the revised version, as well as unanimous strong support by the reviewers.

We generally grant three months as standard revision time. As we are aware that many laboratories cannot function at full capacity owing to the COVID-19 pandemic, we may relax this deadline. Also, we have decided to apply our 'scooping protection policy' to the time span required for you to fully revise your manuscript and address the experimental issues highlighted herein. Nevertheless, please inform us as soon as a paper with related content is published elsewhere.

When preparing your letter of response to the referees' comments, please bear in mind that this will form part of the Review Process File and will therefore be made available online. For more details on our Transparent Editorial Process, please visit our website:
http://emboj.embopress.org/about#Transparent_Process

Before submitting your revised manuscript, deposit any primary datasets and computer code produced in this study in an appropriate public database (see http://msb.embopress.org/authorguide#dataavailability). Please remember to provide a reviewer password, in case such datasets are not yet public. The accession numbers and database names should be listed in a formal "Data Availability" section (placed after Materials & Method). Provide a "Data availability" section even if there are no primary datasets produced in the study.

I thank you again for the opportunity to consider this work for publication and look forward to your revision.

Yours sincerely,

Elisabetta Argenzio, PhD
Editor
The EMBO Journal

Referee #1:

The yeast protein Ice2 has previously been implicated in determining ER structure, ER-plasma membrane contact site maintenance, ER inheritance, and lipid metabolism or trafficking at contact sites between the ER and lipid droplets. How it does all this and whether it is directly involved is not known. This study suggests that Ice2 regulates the Nem1-Spo7 phosphatase complex, which has previously been shown to regulate the PA phosphatase Pah1. Since Pah1 activity is an important determinant of phospholipid levels in cells, it is possible that regulation of Pah1 activity by Ice2 could

explain the various phenotypes of cells lacking Ice2. The study is well-done and convincing but for it to be appropriate for EMBO J or a similar journal substantially more mechanistic insight into Ice2 function is necessary.

1. The study shows that Ice2 interacts with Nem1-Spo7, but there is no evidence that this alters the phosphatase activity of the complex or that the interaction, in turn, affects the activity of Pah1.

2. How can the authors be sure that the effects ICE2 knock out on lipid metabolism are entirely (or mostly) explained by Ice2 regulation of Nem1-Spo7? Does a version of Pah1 that lacks the phosphorylation sites affected by Nem1-Spo7 reverse the effects of ICE2 knockout on lipid metabolism?

3. The Nem1-Spo7 complex localizes to regions of the ER near lipid droplet biogenesis sites (e.g, PMID: 21422231), while Ice2 is all over the ER in growing cells (PMID: 15585575; PMID: 24373967). How would Ice2 encounter the Nem1-Spo7 complex and is there sufficient Ice2 to bind a significant fraction of Nem1-Spo7?

Referee #2:

This paper presents interesting work regarding ER membrane biogenesis in yeast. The ER undergoes significant expansion under different conditions, but the mechanisms by which cells regulate the size of the organelle remain largely unknown. The authors first establish an inducible system for ER membrane biogenesis, based on a dominant negative interactor of Opi1. They then use this system in conjunction with quantitative parameters that describe ER morphology, such as the amounts of tubules and sheets, to perform a genetic screen for proteins that promote or prevent ER expansion. They focus on a major hit that prevents ER expansion, the Ice2 protein. In further studies they demonstrate that Ice2 inhibits Pah1, the major phosphatase that converts phosphatidic acid (PA) into diacylglycerol (DAG). Specifically, Ice2 associates with and inhibits the phosphatase complex Nem1-Spo7, which is required for the activation of Pah1. Overall, this study provides novel and significant insight in an important process. The experiments were well designed and performed. The data are convincing and the paper is well written. It was a pleasure to review this paper. It is clearly acceptable for publication in the EMBO J.

Minor points:

1. Please add the citation of Hu et al 2008 at Line 181.

2. The authors should be more cautious in drawing the conclusion that deletion of ice2 prevents ER expansion upon DTT treatment (Line 190-200), because apparently the ER is rather abnormal after drug treatment. The punctae seen with Rtn1-GFP remain unexplained.

3. It seems that Δnem1 microsomes can dephosphorylat e Pah1 in Figure 6D. Given t hat the source of Pah1-P came from Δnem1 microsomes, t his is unexpect ed. Can the authors please comment on that?

4. The Discussion (lanes 438-441) needs some clarification: Even if PA accumulates and increases the flux through the Kennedy pathway, how does this channel DAG into phospholipid synthesis?

5. How much of the input for the immunoprecipitation was loaded in Figs. 7C and D?

Referee #3:

In this manuscript, Papagiannidis et al. uncovers Ice2p as a regulator of the yeast lipin Pah1 via the Nem1/Spo7 axis. Conditional expression of Ino2-mutant (ino2*, that cannot be inhibited by Opi1p, is used to trigger ER membrane expansion in the yeast KO collection. An elegant microscopy based screening approach was used to identify mutants defective in formation of cortical ER, including ice2Δ. Ice2p has previously been indicated to have a function in lipid metabolism (Tavassoli 2013, Markgraf 2014, but its function remained unclear.

Papagiannidis and colleagues show that the increase membrane biogenesis either upon induction of lipid biosynthesis or by activation of the UPR is severely decreased in ice2Δ, strongly indicating that Ice2 plays a role in promoting membrane biogenesis. Using genetic, biochemical and lipidomic evidence, it is shown that Ice2p stimulates membrane biogenesis by regulating the yeast lipin Pah1, which channels the lipid precursor PA to storage lipid instead of membrane lipids. The authors show that by inhibiting the Nem1/Spo7 phosphatase, Ice2p reduces the activating dephosphorylation of Pah1p, leaving Pah1p in it's inactive hyperphosphorylated state and increasing phospholipid biosynthesis. As the function of Ice2p remained elusive, these findings are of high interest to researchers interested in membrane biogenesis and lipid metabolism. In addition, this proposed role of Ice2p reveals another layer of complexity around the regulation of Lipid/Pah1.

Overall, this manuscript was a pleasure to read. The data is generally of high quality and convincing, well organized and beautifully written. I have only a few minor points that may help improving this nice story.

• The induction of lipid biosynthetic enzymes via the Henry regulatory circuit depends on ino2*-induction via estradiol. A control experiment in which ER morphology is investigated in cells lacking ino2* treated with estradiol should be included.

• In Figure 4B, the authors show reduced expression of the UPR reporter in ice2Δ cells. As the reduction is only significant at a single timepoint, this evidence is quite weak compared to the rest of the manuscript. This could be solved by including additional time points. It would be important to clarify whether ice2Δ are defective in UPR activation. If ER expansion is an adaptive response to UPR, intuitively one may expect that a defect in membrane expansion should result in increased UPR. How do the authors explain this?

• In Figure 5D/E, the authors present lipidomics data showing an interesting increase in triacylglycerol as well as ergosterol esters in ice2Δ. As EE biosynthesis rates are not regulated (directly) by Pah1p, the increase in mol% EE is unexpected. Could this be due to an absolute decrease in PLs, leading to the increase in TAG and EE, instead of an absolute increase in TAG and SE? This issue could be addressed by presenting the absolute values of each lipid.

• Growth data in Figure 5 is presented as the Log of the measured OD, which is unusual. Preferably, this data is provided as OD values without transformation (if need be on a logarithmic axis).

• Ice2p is typically observed throughout the ER by fluorescence microscopy, whereas Nem1p is

observed in discrete puncta. How do the authors explain the huge excess of Ice2p compared to Nem1p? Could Ice2p have additional functions?

• Is Nem1/Spo7 localization altered in ice2Δ mutants?

• In Figure 7 (C, D), it would be nice to show the amount of IPed FLAG-tagged proteins. In addition, a specificity control should be included (a membrane protein that does not co-precipitate with Nem1 and Spo7 under the IP conditions used).

• All data is obtained by growing cells on synthetic defined media (containing 11 uM inositol). The presence of inositol and choline is well established to regulate lipid biosynthesis (Henry, Kohlwein, Carman - 2012). How do the authors think that Ice2p activity/function would be affected by the presence of inositol and/or choline?

• As the authors have Ice2p listed as part of the title, they may want to include it in the introduction.

• Previous work by Tavassoli et al (EMBO rep 2013) has shown interaction between Ice2 and Scs2, the latter of which interacts with Opi1p. This should be discussed.

Point-by-point response

Referee 1

(...) The study is well-done and convincing but for it to be appropriate for EMBO J or a similar journal substantially more mechanistic insight into Ice2 function is necessary.

1. The study shows that Ice2 interacts with Nem1-Spo7, but there is no evidence that this alters the phosphatase activity of the complex or that the interaction, in turn, affects the activity of Pah1.

Subsequent clarification by the referee: ***The results in Fig. 6D are a good first step but they do not demonstrate that Ice2 directly modulates the activity of Nem1/Spo7 and, if it does, how much it affects activity. I was hoping to see them purify the Nem1/Spo7 complex and directly measure its activity on purified Pah1 (or some other substrate). They could then quantitatively determine whether microsomes that either do or do not contain Ice2 affect Nem1/Spo7 activity and by how much. This should be done with a range of Nem1/Spo7 levels. This is a lot to ask since Nem1 and Spo7 have transmembrane domains. If they cannot do it, they should at least discuss the various ways Ice2 might affect Nem1/Spo7 and Pah1 phosphorylation levels. The fact that the effects of NEM1 and ICE2 deletion on Pah1 phosphorylation are additive (last lane of Fig. 6E), suggests that Ice2 may modulate Pah1 phosphorylation in a way that is independent of Nem1/Spo7. Directly measuring Nem1/Spo7 activity should get at this and determine whether the effect of Ice2 on Nem1/Spo7 activity is sufficient to explain changes in Pah1 phosphorylation. It also possible that the effect of ICE2 deletion on Pah1 phosphorylation is a result of a reduction in the amounts of Nem1 and Spo7 in this strain (suggested by Fig. 7A,B) and not because Ice2 directly affects Nem1/Spo7 activity.***

This comment raises three issues:

(1) A defined system to directly measure the effect of Ice2 on Nem1-Spo7 activity would be very useful. However, to purify the transmembrane proteins Nem1 and Spo7 and combine them with Ice2-containing microsomes for an in vitro phosphatase assay is a tall order and out of reach in the context of this revision. As an alternative approach, we modified our existing in vitro assay by replacing microsomes with immunoprecipitated Nem1-Spo7 and Ice2-Nem1-Spo7 complexes. Unfortunately, we could not observe any phosphatase activity towards Pah1 or the alternative substrate p-nitrophenolphosphate. Taking up the referee's suggestion, we therefore discuss how Ice2 might affect Nem1-Spo7 and Pah1, and we propose two mutually non-exclusive possibilities. First, Ice2 could directly inhibit the enzymatic activity of the Nem1-Spo7 complex. Second, we now show that Ice2 clusters Nem1-Spo7 into foci (new Figure 7E, F; also see response to comment 3 below) and Ice2 could therefore sequester Nem1-Spo7 into larger assemblies in which Nem1 is poorly accessible for Pah1 (lines 351-352 and 438-441).

(2) It was unexpected that microsomes from *Δnem1* cells showed residual phosphatase activity towards Pah1 because there is no evidence for another Pah1 phosphatase besides

Nem1. We believe that this Nem1-independent activity may be an artifact of the in vitro assay. For instance, the microsome preparation could contain a phosphatase that can dephosphorylate Pah1 but never encounters Pah1 in cells (lines 306-309). However, the effects of *NEM1* and *ICE2* deletion on Pah1 phosphorylation are not additive. Deletion of *ICE2* increases Pah1 dephosphorylation, deletion of *NEM1* reduces Pah1 phosphorylation, and deletion of *ICE2* does not increase Pah1 dephosphorylation in *Δnem1* cells. Therefore, *ICE2* deletion only affects the Nem1-dependent activity present in this assay, which is consistent with Ice2 modulating Pah1 phosphorylation exclusively in a Nem1-Spo7-dependent manner.

(3) Deletion of *ICE2* indeed causes a small decrease in the abundance of Nem1 and Spo7 (now Figure EV4C). However, this decrease cannot explain the effect of *ICE2* deletion on Pah1 phosphorylation. As Figure 6B shows, *ICE2* deletion causes dephosphorylation of Pah1 in a Nem1-dependent manner, i.e. it *increases* Nem1-Spo7 activity, despite the decreased abundance of the complex. Therefore, loss of Ice2 must enhance the specific activity of Nem1-Spo7. We added a sentence pointing this out (lines 338-341).

2. How can the authors be sure that the effects ICE2 knock out on lipid metabolism are entirely (or mostly) explained by Ice2 regulation of Nem1-Spo7? Does a version of Pah1 that lacks the phosphorylation sites affected by Nem1-Spo7 reverse the effects of ICE2 knockout on lipid metabolism?

There are two ways to ask whether Ice2 affects lipid metabolism by controlling Nem1-Spo7-mediated dephosphorylation of Pah1. The first is to use a phosphomimetic version of Pah1, which cannot be activated by dephosphorylation (see Figure 6A). Such a phosphomimetic would remain inactive in the absence of Ice2 and thus reverse the effects of *ICE2* knockout. We suspect that this is the experiment the referee had in mind. We replaced wild-type Pah1 with the phosphomimetic pah1(7D/E) variant (Hsieh 2016, PMID 27044741) but found that pah1(7D/E) supports normal lipidome composition and ER morphology. We have to conclude that pah1(7D/E) has similar activity as wild-type Pah1 and is not a useful tool, which has been noted also by others (Soste 2019, PMID 31521608). The second approach is to use a phosphorylation-deficient version of Pah1, which lacks phosphorylation sites regulated by Nem1-Spo7, cannot be inactivated and should reverse the effect of *ICE2* overexpression. We replaced wild-type Pah1 with pah1(7A) and confirmed that it is less phosphorylated than wild-type Pah1 and causes lipidomic changes that are qualitatively identical to those in *Δice2* cells, (new Figures EV5A and 8A, lines 360-369). Moreover, pah1(7A) blocked ER expansion upon *ICE2* overexpression (Figure 8B), arguing that Ice2 needs to inactivate Pah1 to cause ER expansion. Nonetheless, to indicate that this issue is not fully resolved, we phrased our conclusion more cautiously and now state: "These data support the notion that Ice2 promotes ER membrane biogenesis by inhibiting Pah1, although we cannot formally exclude that Ice2 acts by additional mechanisms." (lines 376-378).

3. The Nem1-Spo7 complex localizes to regions of the ER near lipid droplet biogenesis sites (e.g, PMID: 21422231), while Ice2 is all over the ER in growing cells (PMID: 15585575; PMID: 24373967). How would Ice2 encounter the Nem1-Spo7 complex and is there sufficient Ice2 to bind a significant fraction of Nem1-Spo7?

We now show that Ice2 and Spo7 are similarly abundant, whereas the abundance of Nem1 is much lower (new Figure EV4B, lines 336-338). All three proteins co-localize throughout the ER and in discrete foci (new Figure 7E). Furthermore, the formation of these foci requires the presence of Ice2 (new Figure 7F). Hence, there is sufficient Ice2 to bind a significant fraction of the Nem1-Spo7 complex and there is extensive co-localization. Whether the discrete foci are sites of lipid droplet biogenesis is unclear because we observed no obvious co-localization of the Ice2-containing foci with Seipin or lipid droplets (new Figure EV4D, lines 346-349).

Referee 2

(...) Overall, this study provides novel and significant insight in an important process. The experiments were well designed and performed. The data are convincing and the paper is well written. It was a pleasure to review this paper. It is clearly acceptable for publication in the EMBO J.

Minor points:

1. Please add the citation of Hu et al 2008 at Line 181.

Done.

2. The authors should be more cautious in drawing the conclusion that deletion of ice2 prevents ER expansion upon DTT treatment (Line 190-200), because apparently the ER is rather abnormal after drug treatment. The puncta seen with Rtn1-GFP remain unexplained.

We did not mean to suggest that deletion of *ICE2* prevents DTT-induced ER expansion but wanted to convey that ER expansion is aberrant. To indicate this more clearly we rephrased the text and now state that "Image quantification suggested that ER expansion was retarded in *Δice2* cells. (...) However, closer inspection of images of wild-type and *Δice2* cells revealed that ER expansion in *Δice2* mutants was not simply retarded but aberrant." (lines 191-198). The intiguing Rtn1-GFP puncta indeed are unexplained, as we point out (lines 200-202), and we are currently investigating them as part of a separate study.

3. It seems that Δnem1 microsomes can dephosphorylate Pah1 in Figure 6D. Given that the source of Pah1-P came from Δnem1 microsomes, this is unexpected. Can the authors please comment on that?

We agree that the residual Pah1 phosphatase activity in *Δnem1* microscomes is unexpected. There is no evidence for another genuine Pah1 phosphatase besides Nem1 and we suspect that the activity is an artifact of the in vitro assay. For instance, the microsome preparation could contain a phosphatase that can dephosphorylate Pah1 but never encounters Pah1 in cells. We have added a comment to this effect (lines 306-309). Importantly, in our assay Ice2 affects only the Nem1-dependent and not the Nem1-independent Pah1 phosphatase activity.

4. The Discussion (lines 438-441) needs some clarification: Even if PA accumulates and increases the flux through the Kennedy pathway, how does this channel DAG into phospholipid synthesis?

The idea, first articulated by Craddock 2015 (PMID 25862304) and Jacquemyn 2017 (PMID 29074503), is that an accumulation of PA may allosterically activate CCT and thereby increase the incorporation of DAG into PC. We rephrased our explanation in the discussion, which now reads: "This incongruence may be resolved by the finding that the rate-limiting enzyme for phosphatidylcholine synthesis by the Kennedy pathway, CCT, is activated by PA, be it by direct allosteric regulation as in A. thaliana or by more indirect means as in mice (Craddock et al, 2015; Zhang et al, 2019). Thus, accumulation of PA may favor conversion of DAG into phosphatidylcholine, thereby drawing it away from conversion into TAG and deposition in lipid droplets (Jacquemyn et al, 2017)." (lines 463-468). We hope this edit clarifies the issue.

5. How much of the input for the immunoprecipitation was loaded in Figs. 7C and D?

Three percent of the input was loaded and we added this information on the figures (now Figure 7A-C) and in the methods section (lines 851-853).

<u>Referee 3</u>

(...) Overall, this manuscript was a pleasure to read. The data is generally of high quality and convincing, well organized and beautifully written. I have only a few minor points that may help improving this nice story.

1. The induction of lipid biosynthetic enzymes via the Henry regulatory circuit depends on ino2*-induction via estradiol. A control experiment in which ER morphology is investigated in cells lacking ino2* treated with estradiol should be included.

We now show that estradiol does not change ER morphology in cells lacking ino2* (new Figure EV1).

2. In Figure 4B, the authors show reduced expression of the UPR reporter in ice2Δ cells. As the reduction is only significant at a single timepoint, this evidence is quite weak compared to the rest of the manuscript. This could be solved by including additional time points. It would be important to clarify whether ice2Δ are defective in UPR activation. If ER expansion is an adaptive response to UPR, intuitively one may expect that a defect in membrane expansion should result in increased UPR. How do the authors explain this?

To clarify this issue, we measured UPR induction with the transcriptional reporter and also an alternative reporter based on *HAC1* mRNA splicing, and we used tunicamycin as an additional ER stressor besides DTT. All combinations of UPR reporter and ER stressor show that deletion of *ICE2* impairs UPR induction (Fig 4B and new Figure EV2). This impairment of UPR signaling may stem from defective clustering of the UPR signal transducer Ire1 in the absence of Ice2 (lines 195-196). Hence, the defect in membrane expansion may not result in an increased UPR because intact membrane expansion itself is a prerequisite for proper UPR signaling.

3. In Figure 5D/E, the authors present lipidomics data showing an interesting increase in triacylglycerol as well as ergosterol esters in ice2Δ. As EE biosynthesis rates are not regulated (directly) by Pah1p, the increase in mol% EE is unexpected. Could this be due to an absolute decrease in PLs, leading to the increase in TAG and EE, instead of an absolute increase in TAG and SE? This issue could be addressed by presenting the absolute values of each lipid.

The increase in ergosterol esters in *Δice2* cells is indeed surprising, although it has been reported before (Markgraf 2014, PMID 24373967, Figure S2). We replotted the data as lipid/protein ratios in µmole lipid per gram total protein, but the increases in TAG and ergosterol esters remain (new Figure EV3D). In addition, we found an increase in ergosterol esters not only upon *ICE2* deletion but also upon expression of the constitutively active pah1(7A), suggesting a yet undefined link between Pah1 activity and ergosterol ester synthesis or perhaps between TAG levels and ergosterol ester synthesis (new Figure 8A).

4. Growth data in Figure 5 is presented as the Log of the measured OD, which is unusual. Preferably, this data is provided as OD values without transformation (if need be on a logarithmic axis).

The reviewer is right and we now present the data without log transformation.

5. Ice2p is typically observed throughout the ER by fluorescence microscopy, whereas Nem1p is observed in discrete puncta. How do the authors explain the huge excess of Ice2p compared to Nem1p? Could Ice2p have additional functions?

We now show that Ice2 and Spo7 are similarly abundant, whereas the abundance of Nem1 is much lower (new Figure EV4B, lines 336-338). All three proteins co-localize throughout the ER and in discrete foci (new Figure 7E). Given the physical association of Ice2 with Nem1 and Spo7 (Figure 7A, B) and the known interaction between Nem1 and Spo7 (Siniossoglou 1998, PMID 9822591), we suggest that the proteins form a ternary complex. We now point out in the discussion that this ternary complex could contain several molecules of Ice2 and Spo7 per molecule of Nem1 (lines 436-438). Nonetheless, additional functions of Ice2 remain possible.

6. Is Nem1/Spo7 localization altered in ice2Δ mutants?

We thank the reviewer for the suggestion to test this. Indeed, Nem1 and Spo7 no longer form foci in the absence of Ice2 (new Figure 7F). Based on these data we propose that Ice2 could restrain the activity of the Nem1-Spo7 complex at least in part by sequestration into clusters in which Nem1 may be poorly accessible for Pah1 (lines 351-352 and 438-441).

7. In Figure 7 (C, D), it would be nice to show the amount of IPed FLAG-tagged proteins. In addition, a specificity control should be included (a membrane protein that does not co-precipitate with Nem1 and Spo7 under the IP conditions used).

We added panels showing the amount of IPed FLAG-tagged proteins and show the abundant ER membrane protein Dpm1 as a specificity control (Figure 7A, B).

8. All data is obtained by growing cells on synthetic defined media (containing 11 uM inositol). The presence of inositol and choline is well established to regulate lipid biosynthesis (Henry, Kohlwein, Carman - 2012). How do the authors think that Ice2p activity/function would be affected by the presence of inositol and/or choline?

Currently, we do not know how the activity of Ice2 is controlled and if it is sensitive to inositol or choline. The regulation of Ice2 certainly is an important topic for future research (lines 441-445).

9. As the authors have Ice2p listed as part of the title, they may want to include it in the introduction.

We considered this but opted to introduce Ice2 only after describing the genetic screen that identified Ice2. We felt that including Ice2 already in the introduction would confuse the reader because it would be unclear why we did not investigate Ice2 straightaway.

10. Previous work by Tavassoli et al (EMBO rep 2013) has shown interaction between Ice2 and Scs2, the latter of which interacts with Opi1p. This should be discussed.

Scs2 indeed binds to Opi1, but the Scs2-Ice2 interaction that Tavassoli et al reported is a genetic, not a physical one. The negative genetic interaction between Scs2 and Ice2 (i.e. the synthetic growth defect of *Δscs2 Δice2* double mutants) could be explained in various ways, including in ways unrelated to Opi1. For example, Scs2 functions in ER-PM tethering, disruption of which causes ER stress (Manford 2012, PMID 23237950). We show that *ICE2* deletion impairs UPR signaling and thus likely sensitizes cells to ER stress, which may explain why combined deletion of *SCS2* and *ICE2* yields a synthetic growth defect. Such ideas are speculative at this point and we would prefer to keep them out of the manuscript.

omplex

Dear Dr. Schuck,

Thank you for submitting your revised manuscript. Please excuse the delay in communicating this decision to you, which was due to delayed referee responses over the summer holiday period, as well as absences from the office. We have now received the reports from the three initial referees (see comments below) and I am pleased to say that they overall find that their comments have been satisfactorily addressed and now support publication. Therefore, I would now ask you to address a number of final editorial issues that are listed in detail below. Once these remaining issues are resolved, we will be happy to formally accept the manuscript for publication.

Thank you again for giving us the chance to consider your manuscript for The EMBO Journal. I look forward to receiving your final revision. Please feel free to contact me if you have further questions.

Kind regards,

Stefanie Boehm

Stefanie Boehm
Editor
The EMBO Journal

-

Referee #1:

My concerns have been addressed and favor publication of this important and well done study.

Referee #2:

The authors have satisfactorily answered the points raised by us. From our persepective, the paper is acceptable for publication in the EMBO-J.

Referee #3:

I liked this manuscript the first time I reviewed it and only had a few minor comments that have been addressed in full. Moreover the finding that Ice2 is necessary for Nem1/Spo7 clusters is an interesting new addition. Clearly, there are mechanistic questions that remain open and that will certainly be the focus of future research. However, as it stands, this manuscript is a major step forward in understanding the regulation of a critical step in lipid synthesis and how it impinges on organelle size control. The data is of of high quality and presented in a clear and compelling manner.In summary, this will become an influential study in the field of organelle homeostasis and I see it as of great value for EMBO journal readers.

2nd Authors' Response to Reviewers **1st Sep 2021**

The authors performed the requested editorial changes.

2nd Revision - Final Decision 3rd Sep 2021

Thank you again for submitting the final revised version of your manuscript for our consideration. I am pleased to inform you that we have now accepted it for publication in The EMBO Journal.

EMBO PRESS

YOU MUST COMPLETE ALL CELLS WITH A PINK BACKGROUND

PLEASE NOTE THAT THIS CHECKLIST WILL BE PUBLISHED ALONGSIDE YOUR PAPER

Corresponding Author Name: Sebastian Schuck

Journal Submitted to: The EMBO Journal
Manuscript Number: EMBOJ-2021-107958

USEFUL LINKS FOR COMPLETING THIS FORM

http://www.antibodypedia.com
http://1degreebio.org
http://www.equator-network.org/reporting-guidelines/improving-bioscience-research-repor
http://grants.nih.gov/grants/olaw/olaw.htm
http://www.mrc.ac.uk/Ourresearch/Ethicsresearchguidance/Useofanimals/index.htm
http://ClinicalTrials.gov
http://www.consort-statement.org
http://www.consort-statement.org/checklists/view/32-consort/66-title
http://www.equator-network.org/reporting-guidelines/reporting-recommendations-for-tumo
http://datadryad.org
http://figshare.com
http://www.ncbi.nlm.nih.gov/gap
http://www.ebi.ac.uk/ega
http://biomodels.net/
http://biomodels.net/miriam/
http://jjj.biochem.sun.ac.za
https://osp.od.nih.gov/biosafety-biosecurity-and-emerging-biotechnology/
http://www.selectagents.gov/

Reporting Checklist For Life Sciences Articles (Rev. June 2017)

This checklist is used to ensure good reporting standards and to improve the reproducibility of published results. These guidelines are consistent with the Principles and Guidelines for Reporting Preclinical Research issued by the NIH in 2014. Please follow the journal's authorship guidelines in preparing your manuscript.

A- Figures
1. Data
The data shown in figures should satisfy the following conditions:

- the data were obtained and processed according to the field's best practice and are presented to reflect the results of the experiments in an accurate and unbiased manner.
- figure panels include only data points, measurements or observations that can be compared to each other in a scientifically meaningful way.
- graphs include clearly labeled error bars for independent experiments and sample sizes. Unless justified, error bars should not be shown for technical replicates.
- if n< 5, the individual data points from each experiment should be plotted and any statistical test employed should be justified
- Source Data should be included to report the data underlying graphs. Please follow the guidelines set out in the author ship guidelines on Data Presentation.

2. Captions

Each figure caption should contain the following information, for each panel where they are relevant:

- a specification of the experimental system investigated (eg cell line, species name).
- the assay(s) and method(s) used to carry out the reported observations and measurements
- an explicit mention of the biological and chemical entity(ies) that are being measured.
- an explicit mention of the biological and chemical entity(ies) that are altered/varied/perturbed in a controlled manner.

- the exact sample size (n) for each experimental group/condition, given as a number, not a range;
- a description of the sample collection allowing the reader to understand whether the samples represent technical or biological replicates (including how many animals, litters, cultures, etc.).
- a statement of how many times the experiment shown was independently replicated in the laboratory.
- definitions of statistical methods and measures:
  - common tests, such as t-test (please specify whether paired vs. unpaired), simple χ2 tests, Wilcoxon and Mann-Whitney tests, can be unambiguously identified by name only, but more complex techniques should be described in the methods section;
  - are tests one-sided or two-sided?
  - are there adjustments for multiple comparisons?
  - exact statistical test results, e.g., P values = x but not P values < x;
  - definition of 'center values' as median or average;
  - definition of error bars as s.d. or s.e.m.

Any descriptions too long for the figure legend should be included in the methods section and/or with the source data.

In the pink boxes below, please ensure that the answers to the following questions are reported in the manuscript itself. Every question should be answered. If the question is not relevant to your research, please write NA (non applicable). We encourage you to include a specific subsection in the methods section for statistics, reagents, animal models and human subjects.

B- Statistics and general methods

Please fill out these boxes (Do not worry if you cannot see all your text once you press return)

1.a. How was the sample size chosen to ensure adequate power to detect a pre-specified effect size?	NA
1.b. For animal studies, include a statement about sample size estimate even if no statistical methods were used.	NA
2. Describe inclusion/exclusion criteria if samples or animals were excluded from the analysis. Were the criteria pre-established?	NA
3. Were any steps taken to minimize the effects of subjective bias when allocating animals/samples to treatment (e.g. randomization procedure)? If yes, please describe.	NA
For animal studies, include a statement about randomization even if no randomization was used.	NA
4.a. Were any steps taken to minimize the effects of subjective bias during group allocation or/and when assessing results (e.g. blinding of the investigator)? If yes please describe.	Image were anonymized prior to analysis using the "blind analysis tools" plugin in ImageJ.
4.b. For animal studies, include a statement about blinding even if no blinding was done	NA
5. For every figure, are statistical tests justified as appropriate?	Yes (see section "experimental design" in Materials and Methods).
Do the data meet the assumptions of the tests (e.g., normal distribution)? Describe any methods used to assess it.	Sampling was random and samples were independent in all cases (see section "experimental design" in Materials and Methods).
Is there an estimate of variation within each group of data?	Yes, standard error of the mean.

Is the variance similar between the groups that are being statistically compared?	Yes, except in cases of data normalization (see section "experimental design" in Materials and Methods).

C- Reagents

6. To show that antibodies were profiled for use in the system under study (assay and species), provide a citation, catalog number and/or clone number, supplementary information or reference to an antibody validation profile. e.g., Antibodypedia (see link list at top right), 1DegreeBio (see link list at top right).	Clone numbers provided in methods section.
7. Identify the source of cell lines and report if they were recently authenticated (e.g., by STR profiling) and tested for mycoplasma contamination.	NA

* for all hyperlinks, please see the table at the top right of the document

D- Animal Models

8. Report species, strain, gender, age of animals and genetic modification status where applicable. Please detail housing and husbandry conditions and the source of animals.	NA
9. For experiments involving live vertebrates, include a statement of compliance with ethical regulations and identify the committee(s) approving the experiments.	NA
10. We recommend consulting the ARRIVE guidelines (see link list at top right) (PLoS Biol. 8(6), e1000412, 2010) to ensure that other relevant aspects of animal studies are adequately reported. See author guidelines, under 'Reporting Guidelines'. See also: NIH (see link list at top right) and MRC (see link list at top right) recommendations. Please confirm compliance.	NA

E- Human Subjects

11. Identify the committee(s) approving the study protocol.	NA
12. Include a statement confirming that informed consent was obtained from all subjects and that the experiments conformed to the principles set out in the WMA Declaration of Helsinki and the Department of Health and Human Services Belmont Report.	NA
13. For publication of patient photos, include a statement confirming that consent to publish was obtained.	NA
14. Report any restrictions on the availability (and/or on the use) of human data or samples.	NA
15. Report the clinical trial registration number (at ClinicalTrials.gov or equivalent), where applicable.	NA
16. For phase II and III randomized controlled trials, please refer to the CONSORT flow diagram (see link list at top right) and submit the CONSORT checklist (see link list at top right) with your submission. See author guidelines, under 'Reporting Guidelines'. Please confirm you have submitted this list.	NA
17. For tumor marker prognostic studies, we recommend that you follow the REMARK reporting guidelines (see link list at top right). See author guidelines, under 'Reporting Guidelines'. Please confirm you have followed these guidelines.	NA

F- Data Accessibility

18: Provide a "Data Availability" section at the end of the Materials & Methods, listing the accession codes for data generated in this study and deposited in a public database (e.g. RNA-Seq data: Gene Expression Omnibus GSE39462, Proteomics data: PRIDE PXD000208 etc.) Please refer to our author guidelines for 'Data Deposition'.<br><br>Data deposition in a public repository is mandatory for:<br>a. Protein, DNA and RNA sequences<br>b. Macromolecular structures<br>c. Crystallographic data for small molecules<br>d. Functional genomics data<br>e. Proteomics and molecular interactions	NA
19. Deposition is strongly recommended for any datasets that are central and integral to the study; please consider the journal's data policy. If no structured public repository exists for a given data type, we encourage the provision of datasets in the manuscript as a Supplementary Document (see author guidelines under 'Expanded View' or in unstructured repositories such as Dryad (see link list at top right) or Figshare (see link list at top right).	NA
20. Access to human clinical and genomic datasets should be provided with as few restrictions as possible while respecting ethical obligations to the patients and relevant medical and legal issues. If practically possible and compatible with the individual consent agreement used in the study, such data should be deposited in one of the major public access-controlled repositories such as dbGAP (see link list at top right) or EGA (see link list at top right).	NA
21. Computational models that are central and integral to a study should be shared without restrictions and provided in a machine-readable form. The relevant accession numbers or links should be provided. When possible, standardized format (SBML, CellML) should be used instead of scripts (e.g. MATLAB). Authors are strongly encouraged to follow the MIRIAM guidelines (see link list at top right) and deposit their model in a public database such as Biomodels (see link list at top right) or JWS Online (see link list at top right). If computer source code is provided with the paper, it should be deposited in a public repository or included in supplementary information.	Computer source code available under https://github.com/SchuckLab/ClassifiER om Github

G- Dual use research of concern

22. Could your study fall under dual use research restrictions? Please check biosecurity documents (see link list at top right) and list of select agents and toxins (APHIS/CDC) (see link list at top right). According to our biosecurity guidelines, provide a statement only if it could.	NA
